# Allele-Specific Gene Expression Is Widespread Across the Genome and Biological Processes

**DOI:** 10.1371/journal.pone.0004150

**Published:** 2009-01-07

**Authors:** Ricardo Palacios, Elodie Gazave, Joaquín Goñi, Gabriel Piedrafita, Olga Fernando, Arcadi Navarro, Pablo Villoslada

**Affiliations:** 1 Neuroimmunology Laboratory, Center for Applied Medical Research (CIMA), University of Navarra, Pamplona, Spain; 2 Unitat de Biologia Evolutiva. Universitat Pompeu Fabra, Barcelona, Spain; 3 Department of Physics and Applied Mathemaics, University of Navarra, Pamplona, Spain; 4 Instituto de Tecnologia Química e Biológica (ITQB), Universidade Nova de Lisboa, Lisboa, Portugal; 5 Institucio Catalana de recerca i Estudis Avançats (ICREA), Barcelona, Spain; 6 CIBER Epidemiología y Salud Pública (CIBERESP), Barcelona, Spain; 7 Department of Neurology, Hospital Clinic – IDIBAPS, Barcelona, Spain; Ecole Normale Supérieure de Lyon, France

## Abstract

Allelic specific gene expression (ASGE) appears to be an important factor in human phenotypic variability and as a consequence, for the development of complex traits and diseases. In order to study ASGE across the human genome, we have performed a study in which genotyping was coupled with an analysis of ASGE by screening 11,500 SNPs using the Mapping 10 K Array to identify differential allelic expression. We found that from the 5,133 SNPs that were suitable for analysis (heterozygous in our sample and expressed in peripheral blood mononuclear cells), 2,934 (57%) SNPs had differential allelic expression. Such SNPs were equally distributed along human chromosomes and biological processes. We validated the presence or absence of ASGE in 18 out 20 SNPs (90%) randomly selected by real time PCR in 48 human subjects. In addition, we observed that SNPs close to -but not included in- segmental duplications had increased levels of ASGE. Finally, we found that transcripts of unknown function or non-coding RNAs, also display ASGE: from a total of 2,308 intronic SNPs, 1510 (65%) SNPs underwent differential allelic expression. In summary, ASGE is a widespread mechanism in the human genome whose regulation seems to be far more complex than expected.

## Introduction

Allelic-specific gene expression (ASGE) or allelic imbalance appears to be an important factor for human phenotypic variability and as a consequence, for the development of common diseases [1]. Traditionally, ASGE has been associated with the phenomena of X-chromosome inactivation and genomic imprinting [2]. However, several recent studies have emphasized the extent to which gene expression varies within and between populations [3], [4], [5], [6], and it is now clear that ASGE is relatively common among non-imprinted autosomal genes [7], [8], [9]. Furthermore, certain genes display allelic variation in gene expression that is transmitted by Mendelian inheritance and this variation may be linked to common human disorders [8], [10], [11].

Variation in gene expression may result from changes in the sequence of regulatory elements, such as single nucleotide polymorphisms (SNPs), and recent surveys indicates that this phenomenon is widespread through the genome and tissues [3], [12], [13], [14]. Such changes may explain up to 25 to 35% of the interindividual differences in allelic gene expression [15], [16]. Hence, identification and characterization of ASGE will help us to appreciate the extent of functionally important regulatory variation. In turn, this will enable us and to focus on candidate haplotypes whose allelic differences in expression may provide an important link between individual genetic variation and complex traits or common diseases.

In order to study ASGE across the human genome, we have performed a study in which genotyping was coupled with an analysis of allele-specific gene expression by screening 11,560 SNPs using the Mapping 10 K Array (Affymetrix) to identify differential allelic expression. We found that ASGE is very common in the human genome and that it is widespread in many biological processes. We validated our findings using a new cohort of 48 subjects. In addition, we observed that SNPs close to -but not included in- segmental duplications had increased levels of ASGE and we assessed the effect of copy number variation (CNV) using a 44 K Agilent probe array in the same individuals. Finally, we found that transcripts of non-coding RNA (ncRNA) also display allelic imbalance.

## Results

### Allele-specific expression screening

Because we screened for ASGE in peripheral blood mononuclear cells (PBMCs), SNPs suitable for analysis had to meet the following criteria: (1) at least one individual was heterozygous for the SNP; and (2) the transcript containing the SNP is expressed in PBMCs. We screened 20 individuals founding at least one heterozygous individual for 10,837 SNPs out of the 11,560 SNPs in the 10 K Mapping Array. Of these SNPs, 5,133 corresponded to transcripts expressed in PBMCs (see Methods). Thus, these 5,133 SNPs constitute the analyzed in this study. After the significance was established, the ASGE ratio was considered significant if it was bigger than 1.37 or smaller than 0.81. We found that 2,934 out of 5,133 (57%) SNPs were subject to allelic imbalance: 2,235 SNPs (76%) had a ratio between the expression intensity of the two alleles lower than two, 476 SNPs (16%) displayed a ratio between two- and threefold, and for 223 SNPs (8%) the ratio was greater than a threefold excess for at least one individual. In contrast, 2,199 SNPs (43%) did not display significantly different levels of expression between the two alleles. A complete list of all the SNPs studied and their characteristics can be found in the Supplementary Table S1.

As predicted, in female, the percentage of SNPs with differentially expressed alleles on the X chromosome was significantly higher (Chi-square test, p<0.001) than on autosomic chromosomes since genes subject to X-chromosome inactivation are expected to display skewed allelic expression [17]. Indeed, we were able to identify five known imprinted genes that met the criteria established for the analysis of allelic expression: KCNQ1, MEG3, PPP1R9A, SLC22A3 and SLC22A23. We confirmed ASGE for the first four (Table 1).

**Table 1:** Differential allele expression ratios for imprinted genes.

Imprinted gene	dbSNP RS ID	Alleles	Location	ASGE (p<0.01)	ASGE ratio	No. of individuals studied	Percent of individuals studied with ASGE
**KCNQ1**	rs63934	C+/T	11p15.5	Yes	1,76±1,06	8	63%
**MEG3**	rs721910	A/C	14q32.2	No	1,23	1	—
**MEG3**	rs721909	A+/G	14q32.2	Yes	1,38±0,41	2	50%
**PPP1R9A**	rs2374983	A+/G	7q21.3	Yes	1,49	1	100%
**SLC22A23**	rs4128536	A/G	6p25.2	No	1,00	1	—
**SLC22A23**	rs4128535	A/G	6p25.2	No	1,05±0,07	2	—
**SLC22A3**	rs2174914	C/G+	6q25.3	Yes	1,29±0,44	7	29%

Results are presented as the mean±SD. Values are the ratios (Allele 1/Allele 2) between the two alleles. The values were inverted if less than one (Allele 2/Allele 1, when Allele 2 was preferentially expressed). The preferentially expressed allele is labelled with “+”.

In order to validate the results of the Mapping 10 K Array experiments, we performed allele-specific quantitative PCR for 20 SNPs randomly selected in forty-eight new subjects. The results of the real-time quantitative PCR validated the results of the screening since we confirmed the allele-specific or non allele-specific expression in 18 of these 20 SNPs (90%; Table 2), suggesting a low false positive discovery rate. These results validate our experimental method as well as our sample handling and processing.

**Table 2:** Validation of differential allele expression ratios for 20 SNPs randomly selected in forty-eight new subjects by real-time quantitative PCR (rtPCR).

dbSNP ID	Consequence type (Variation Feature)	Gene	Alleles	Location	ASGE by rtPCR (p<0.01)	ASGE ratio by rtPCR	Percent of individuals studied with ASGE by rtPCR	ASGE in 10 K array (p<0.01)	ASGE ratio in 10 K array	No of individuals studied in 10 K array	Percent of individuals studied with ASGE in 10 K array
**rs1353761**	Intergenic	—	A+/T	12q23.2	Yes	>8	100%	Yes	1,75±1,00	9	44%
**rs1372788**	Intergenic	—	A/C	4q21.1	No	2.86±3.69	—	No	1,14	1	—
**rs1408814**	Intergenic	—	A/G+	10q11.21	Yes	1.39±0.18	75%	Yes	1,49±0,38	10	40%
**rs1442696**	Intergenic	—	C+/T	4q21.1	Yes	2.10±0.22	88%	Yes	2,70±1,31	4	100%
**rs1443753**	Intronic	AP4M1	C/T	7q31.32	Yes	>8	100%	No	1,20±0,23	2	—
**rs1512981**	Intergenic	—	C/T+	12q15	Yes	1.44±1.08	63%	Yes	2,19±1,98	3	33%
**rs1986518**	Intergenic	—	C/T	1p32.3	Yes	5.31±0.07	63%	Yes	1,41±0,23	4	50%
**rs1992116**	Intronic	MMP2	C/T	16q12.2	Yes	6.75±7.34	88%	Yes	2,78±1,06	4	100%
**rs231799**	Intergenic	—	C/T	2q33.2	Yes	1.21±0.50	38%	Yes	1,40±0,31	3	67%
**rs2367737**	Intergenic	—	C/G	7q21.11	No	1.81±0.85	—	No	1,16	1	—
**rs2394075**	Intergenic	—	A/T	10q21.3	No	1.06±0.10	—	No	1,07	1	—
**rs2870951**	Intergenic	—	C/T	12q15	Yes	1.80±0.78	88%	Yes	1,71±0,97	7	43%
**rs34385187**	Intergenic	—	A+/G	1p13.2	Yes	1.27±0.04	75%	Yes	1,71±0,85	2	50%
**rs722290**	Intronic	STYX	C/G	14q22.1	Yes	1.92±3.12	100%	Yes	1,27±0,19	6	17%
**rs722749**	Intergenic	—	C/G	12q15	Yes	3.32±1.95	100%	Yes	1,96±1,10	6	67%
**rs723765**	Intronic	ROBO1	A/G+	3p12.3	Yes	1.64±0.15	75%	Yes	1,28±0,43	8	25%
**rs727363**	Intronic	FAM113B	A/C+	12q13.11	Yes	2.27±0.54	38%	Yes	1,16±0,12	15	7%
**rs950000**	Intergenic	—	A/G+	10p11.23	No	1.01±0.11	—	Yes	1,43±0,68	4	25%
**rs959727**	Intronic	FSTL4	C+/T	5q31.1	Yes	4.35±0.09	100%	Yes	3,51	1	100%
**rs985933**	Intronic	HTR2A	A+/G	13q14.2	Yes	5.00±0.15	88%	Yes	1,56±0,76	2	50%

Results are presented as the mean±SD. Values are the ratios (Allele 1/Allele 2) between the two alleles. The values were inverted if less than one (Allele 2/Allele 1, when Allele 2 was preferentially expressed). The preferentially expressed allele is labelled with “+”.

### Allele-specific expression is widespread across the human genome and in different biological processes

**Figure 1:**
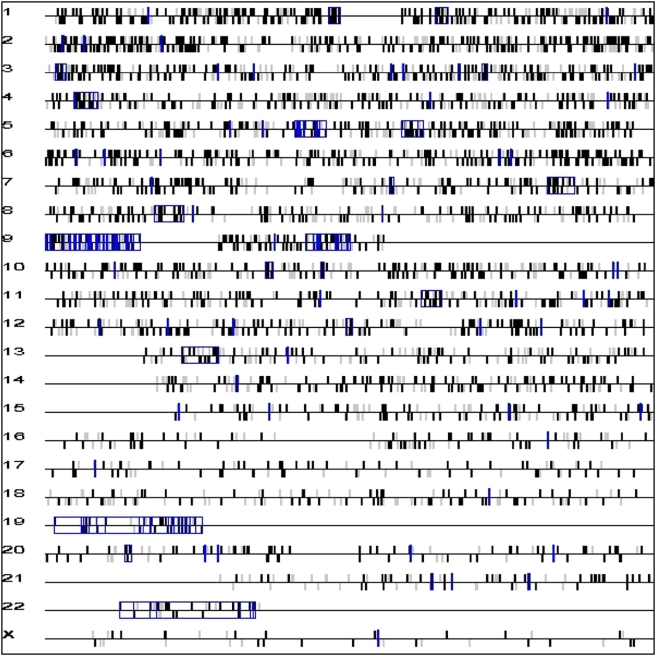
Chromosome mapping of heterozygous SNPs expressed in PBMCs. The position of each SNP on the chromosome is based on the annotation in dbSNP (version 126, May 2006). Differentially expressed SNP alleles are coloured in black. The vertical bar above the horizontal line means the SNP is on the forward strand, the one below means that it is on the reverse strand. SNP stretches with a p-value<0.00005 are highlighted in blue boxes.

We mapped the SNPs that displayed ASGE to chromosomes in order to look for regions in the human genome with a higher density of such SNPs (Table 3 and Figure 1). When the SNP distribution in each chromosome was analyzed, we found that an average of 57% of the SNPs per chromosome displayed ASGE, the same percentage as for the overall genome, and without any chromosome deviating significantly from this percentage. This is further evidence that ASGE is widespread across the human genome. Furthermore, the “SNP proximity” test (see Methods) was used to search for clusters of differentially expressed allelic SNPs. As a result, we found a total of 133 clusters dispersed throughout the genome with a median of 4 SNPs per cluster (rank 1–36). Localization, length and p-value of clusters can be found in Supplementary Table S2.

**Table 3:** Distribution of differentially expressed alleles of SNPs across chromosomes (assembly March 2006; chr. Y not included).

Chr	No. of SNPs available for analysis	Differentially expressed SNPs	% differentially expressed SNPs	No. Clusters*
1	405	250	62	4
2	459	271	59	3
3	397	231	58	9
4	339	168	50	4
5	343	198	58	12
6	362	229	63	5
7	273	154	56	3
8	257	141	55	3
9	239	138	58	36
10	284	165	58	5
11	271	149	55	5
12	253	138	55	6
13	196	90	46	2
14	196	111	57	1
15	149	85	57	4
16	117	59	50	1
17	89	58	65	1
18	126	63	50	1
19	52	34	65	15
20	112	80	71	5
21	86	47	55	3
22	38	23	61	4
X	42	24	57	1
No mapped	48	28	58	–

* See Figure 1.

The total No. of SNPs mapped was 5,085 of the 5,133 found in the screening due to the fact that there is no annotation for 48 studied SNPs.

The subset of 5,133 SNPs studied corresponded to a total of 1,632 known genes, 1,195 of which displayed allelic imbalance in at least one of their SNPs (73%). In order to assess whether ASGE is more influential in any given biological process, we assessed the distribution of genes that did or did not display differential allele expression in the Gene Ontology (GO) database (www.geneontology.org). The comparison between the distributions of genes among different biological processes (GO terms present in levels 3 to 9) did not demonstrate any significant differences (Supplementary Table S3). Thus, the genes subject to differential allelic expression appear to participate in a wide range of different biological processes.

### Allele-specific gene expression in ncRNA

We also focused our analysis on the recently described transcripts of ncRNA, previously named transcripts of unknown function, that introduce more complex strategies for transcriptional regulation than previously anticipated [18], [19]. Eukaryotic genes contain clearly identifiable open reading frames (ORFs) that direct the translation of functional proteins. However, not all RNA transcripts (other than tRNA, rRNA or snRNA) are translated into polypeptides. Many non-translatable mRNA-like RNA transcripts have been found in the cell. They are polyadenylated, spliced and are lacking long ORFs [20]. In this work, ncRNA are defined as non-coding polyadenylated RNAs that are transcribed but for which there is no functional information. Like eukaryotic messenger RNA, ncRNA contain poly-A tails and thus, they are represented among cDNAs synthesised from mature RNAs using an oligo(dT) primer (see Methods). Adopting this strategy, we measure allele specific expression only of exonic SNPs because, other RNA molecules such as immature RNA that contain introns, are not represented. Surprisingly, in the subset of the 5,133 heterozygous SNPs expressed in PBMCs, a total of 2,311 (45%) and 2,455 (48%) SNPs were intronic and intergenic respectively (Table 4). Of the intronic SNPs, 1,511 (65%) underwent differential allelic expression, as well as 1,190 (48%) of the intergenic SNPs. This result is consistent with the high levels of unannotated transcription detected [18], [19] and it also shows that like known genes, ncRNA display ASGE. We validated ASGE in intronic and intergenic SNPs by real-time quantitative PCR in 18 out of 20 SNPs (Table 2). To check that this finding was not the result of the presence of contaminants such as DNA in the RNA samples after the DNase digestion, we used RNA as template for the quantitative real-time PCR under the same conditions used in the validation experiments. All samples were checked for one intronic SNP without finding any amplification (data not shown).

**Table 4:** Mapping of SNPs.

Consequence type (Variation Feature)	No. of SNPs available for analysis	Differentially expressed SNPs	% differentially expressed SNPs
**No annotation**	57	31	54
**3Prime UTR**	43	32	74
**5Prime UTR**	5	2	40
**Downstream**	119	76	64
**Intergenic**	2.455	1.190	48
**Intronic**	2.311	1.511	65
**Non synonymous coding**	14	12	86
**Synonymous coding**	26	20	77
**Upstream**	103	60	58
**TOTAL**	5.133	2.934	57

Despite we study poliadenilated RNA, intronic and intergenic SNPs also present ASGE. Thus, ncRNA display ASGE similar to that of known genes revealing even more complexity in the system that regulates transcription.

### Allele-specific expression is dependent on regulatory effects associated to segmental duplications

A potential cause of ASGE might be the considerable variation in gene copy number in the human genome [21]. If individuals have different copies of a given duplicon in homologous chromosomes, that is, if they are heterozygous for structural variants and these structural variants contain genes, it is possible that certain alleles appear to be differentially expressed. This differential expression may result simply because they are present in different copy numbers in different chromosomes and not through any regulatory effects. To test this hypothesis, we examined whether the location of a SNP within known structural variants (SDs or CNVs) might affect the probability that it were differentially expressed. As a first test, we used the location of SDs that can be found in public databases (see Methods). Only 106 of the SNPs in our study mapped within known segmental duplication regions and a test showed that SNPs presenting ASGE are not more likely to be located inside SDs than SNPs without ASGE (Table 5), even if the lack of statistical significance may be an effect of small sample size, as we will see below. Another potential cause of ASGE is heterozygosity among cis-regulatory elements. In particular, SDs may contain cis-regulatory elements that affect the expression of nearby genes. If this were a frequent phenomenon, allelic variation in gene expression should be more frequent in single-copy regions that are located in the vicinity of SDs than in single-copy regions far away from duplicons. Using the same dataset than above, we observed that SNPs with ASGE are more frequent near SDs. This enrichment in SNPs with ASGE is especially strong in the 10 Kb windows around SD regions (Table 5). The effect decreases in more distant (non overlapping) windows.

**Table 5:** Details of the chi-square tables and P-values for the dbSD database.

dbSD	Distance of a SNP to the closest SD											
	Inside			10 Kb			100 Kb			1 Mb		
	Out	In		Out	In		Out	In		Out	In	
**No ASGE**	2160	39	2199	2103	57	2160	1667	436	2103	244	1423	1667
**ASGE**	2867	67	2934	2751	116	2867	2089	662	2751	243	1846	2089
	5027	106	5133	4854	173	5027	3756	1098	4854	487	3269	3756
**χ^2^**	1.62			7.34			7.56			1.20		
**P**	0.204			0.007			0.006			0.272		

The distances represent the different windows of distance we considered around each SNPs to test the effect of the proximity of a SD on ASGE. “In” means inside the windows of size considered.

As a second series of tests, we computed the average ratios of allele expression instead of the proportion of SNPs presenting ASGE. Results are presented in Table 6. Interestingly, although the proportion of SNP with and without ASGE located inside SDs were not significantly different, SNPs inside SDs have on average a higher ratio of allele expression. This effect, again, decreases with distance to the SD. This means that the closest a SNP is to a segmental duplication, the strongest the degree of allele specific gene expression, probably because of regulatory effects attributable to SDs. The maximum effect is registered in SNPs located within SDs, which are probably present in different copy numbers in different chromosomes.

**Table 6:** Details of the permutation tests for the mean absolute values of allelic gene expression ratios depending on their position relatively to segmental duplications.

Windows	Category	n	mean	P
**Inside**	Inside SD	106	2.1014	
	Outside	5027	1.5438	<0.001
**10 Kb**	Inside 10 Kb	173	1.6803	
	Outside	4854	1.5389	0.051
**100 Kb**	Inside 100 Kb	1098	1.5724	
	Outside	3756	1.5292	0.183
**1 Mb**	Inside 1 Mb	3269	1.5308	
	Outside	487	1.5183	0.776

Windows of sizes are the same as previously described.

We then used information about known Copy Number Variants (CNV; dbCNV database, see Methods). Similar analyses provide consistent, even if slightly different results. In particular, there are more SNPs with ASGE inside CNVs (Table 7), but these SNPs do not present a higher ratio of gene expression (Table 8). Unlike SNPs near SDs, SNPs within 10 Kb of a CNV do not present any significant effect, probably because of small sample size, since the effect is stronger in the 100 kb window.

**Table 7:** Details of the chi-square tables and P-values for the dbCNV database.

dbCNV	Distance of a SNP to the closest CNV											
	Inside			10 Kb			100 Kb			1 Mb		
	Out	In		Out	In		Out	In		Out	In	
**No ASGE**	1799	400	2199	1750	49	1799	1351	399	1750	182	1169	1351
**ASGE**	2410	524	2934	2355	55	2410	1896	459	2355	241	1655	1896
	4209	924	5133	4105	104	4209	3247	858	4105	423	2824	3247
**χ^2^**	4.60			0.833			6.65			0.403		
**P**	0.032			0.361			0.010			0.526		

The distances represent the different windows of distance we considered around each SNPs to test the effect of the proximity of a CNV on ASGE. “In” means inside the windows of size considered.

**Table 8:** Details of the permutation tests for the mean absolute values of allelic gene expression ratios depending on their position relatively to copy number variants from databases.

Windows	Category	n	mean	P
Inside	Inside SD	924	1.5934	
	Outside	4209	1.5469	0.213
10 Kb	Inside 10 Kb	104	1.5162	
	Outside	4105	1.5477	0.771
100 Kb	Inside 100 Kb	858	1.5724	
	Outside	3247	1.5292	0.080
1 Mb	Inside 1 Mb	2824	1.5726	
	Outside	423	1.4928	0.138

Windows of sizes are the same as previously described.

Because there is high inter-individual variability in CNV and SD content [22], it is possible that some genome regions that contain CNVs or SDs in public databases are in fact single-copy in the individuals included in our study. To try to overcome this problem we studied CNVs in the tested individuals. We used the Agilent 44 K array to create the indCNV dataset, where individual CNV patterns can be associated to the corresponding individual ASGE patterns (see Methods). In this analysis, we retrieve the same trend that we reported above. In table 9, we see that there are more SNPs with ASGE close to the CNVs detected in the studied individuals than far away from these CNVs. However, this effect is not significant. Since the number of CNVs detected in each individual is much lower than the total of SDs and CNVs present in databases, we suggest that, sample size, and thus statistical power, in the vicinity of CNVs is very small.

**Table 9:** Details of the chi-square tables and P-values for the indCNV database.

dbSD	Distance of a SNP to the closest indCNV											
	Inside			10 Kb			100 Kb			1 Mb		
	Out	In		Out	In		Out	In		Out	In	
**No ASGE**	9549	3	9552	9545	4	9549	9535	10	9545	9566	69	9635
**ASGE**	7522	4	7526	7518	4	7522	7503	15	7518	7370	52	7422
	17071	7	17078	17063	8	17071	17038	25	17063	16936	121	17057
**χ^2^**	NA			NA			2.58			0.014		
**P**	NA			NA			0.11			0.905		

dbSD	Distance of a SNP to the closest indCNV		
	Up to 100 Kb		
	Out	In	
**No ASGE**	9535	17	9552
**ASGE**	7503	23	7526
	17038	40	17078
**χ^2^**	2.93		
**P**	0.086		

The distances represent the different windows of distance we considered around each SNPs to test the effect of the proximity of a CNV on ASGE. “In” means inside the windows of size considered.

## Discussion

In this study, we have used high throughput screening of 11,500 SNPs to detect ASGE across the human genome. Our study indicates that allelic variation in gene expression is widespread across the human genome and in different biological processes, including systems of transcriptional regulation. We found that the 57% of human SNPs studied here undergo allelic imbalance and that these SNPs are distributed proportionally among chromosomes. Indeed, among different biological processes we did not find any difference in the distribution of genes that displayed differential allelic expression or those that did not. ASGE is also present in several tissues [21], indicating that this is a common mechanism of genomic regulation for many pathways and cell types. Indeed, ASGE is also implicated in a variety of disease states [23].

Thus, an interesting question that arises is how this ASGE is controlled. A potential cause is the variation in copy number within the human genome [21]. To test for this possibility we examined whether the probability of an SNP undergoing differential allelic expression changes if the SNP is located within SDs or CNVs described in public databases. We found that only a small percentage of the SNPs displaying differentially allele expression were included in these structural variants. However, the proportion of SNPs with differentially expressed alleles was higher in SNPs close to SDs, or within CNVs, suggesting that regulatory elements may lie within these genomic duplications. Moreover, SNPs inside SDs have higher ratio of ASGE, suggesting regulatory effects linked to SDs. Therefore, the relationship between structural variation and ASGE, detected both in variation in intensity and presence/absence of allelic expression, seems to be rather complex. We also studied the contribution of individual CNVs to allelic imbalance, but results of the analysis were not conclusive, probably due to lack of statistical power. Overall, results coming from the analysis of known SDs and CNVs are largely consistent. Still, it may be surprising to see that the association with ASGE is not identical for both databases. One must take into account that the two datasets have different properties. SDs are computationally defined from the Reference Human Genome Assembly, whereas CNVs are structural variants detected by experimental hybridizations. This means that the two datasets are similar, but most certainly not identical. In addition, it must be considered that individuals in this study are likely to present a set of SDs and CNVs that only partially overlaps with those present in public databases. Consequently, in the tests conducted with information from public databases, we may have mislabeled some SNPs, because a large number of structural variations described in dbSD or dbCNV may not be present in the 20 individuals studied here.

Finally, we completed our study by focusing on the implications of ASGE among ncRNA [18], [19]. Thus, our current understanding of the repertoire of transcripts produced from the human genome is still evolving, further demonstrating the complexity of the transcriptome. Indeed, the organization and structure of the genome has potentially important implications for the regulation of transcription and the possible interpretation of the naturally occurring genetic variation in humans [24]. We found that ncRNA display ASGE similar to that of known genes revealing even more complexity in the system that regulates transcription.

In summary, ASGE is widespread across the human genome and it participates in all biological processes, especially in the regulation of gene expression in the immune system. If ASGE has important implications in the genotype to phenotype relations and in the regulation of complex interlaced transcriptional patterns, its identification and characterization will provide a better understanding of the complexities of transcription regulation. Furthermore, such knowledge should allow us to focus on haplotypes with allelic differences in expression that may be linked to complex traits and common diseases.

## Methods

### Subjects

A total of 68 healthy Caucasian individuals were recruited to this study. All of them were of Southern-European origin, which minimize differences in population structure [25], [26]. Twenty of them, 12 male and 8 female, were used for the array screening assays and the rest for the real time quantitative PCR validation. The study was approved by the local ethical committee (IRB) and patients provided written informed consent.

### RNA and DNA purification and cDNA synthesis

Total RNA was extracted from peripheral blood mononuclear cells (PBMCs). PBMCs were isolated from heparinized blood by density gradient centrifugation using Ficoll-Paque (Pharmacia Biotech). PBMCs were immediately submerged in the RNAlater RNA Stabilization Reagent (Qiagen) to preserve their gene expression patterns and total RNA was isolated using the RNeasy Mini Kit (Qiagen). During RNA purification, DNA was removed with a DNase treatment using the RNase-Free DNase Set (Qiagen). Genomic DNA (gDNA) was isolated from granulocytes obtained after density gradient centrifugation using the QIAamp DNA Mini Kit (Qiagen). Synthesis of cDNA for the array screening assays was performed on 2 µg of total RNA using a T7-oligo dT12–18 primer (Amersham Pharmacia) and it was purified using phenol∶chloroform∶isoamyl alcohol and NH_4_Ac precipitation. The cDNA pellet was resuspended in 5 µl of reduced EDTA TE buffer (10 mM Tris HCl, pH 8.0, 0.1 mM EDTA, pH 8.0). Synthesis of cDNA for the real time quantitative PCR validation was performed using the High-Capacity cDNA Archive Kit (Applied Biosystems).

### Mapping 10 K Array experiments

Genotyping and allele specific gene expression was assessed using GeneChip Mapping 10 K Arrays (Affymetrix) according to the manufacturer's instructions using either 250 ng of gDNA or 250 ng of cDNA as the starting material. Allele calling was made by using the GeneChip DNA Analysis Software 2.0 (Affymetrix).

### Allele expression data

A computational analysis of allele specific gene expression was carried out as previously described [9]. Briefly, to be included in our analysis, each SNP had to meet the following criteria: (1) at least one out of twenty individuals must be heterozygous for the SNP; and (2) the transcript containing the SNP must be expressed in PBMCs. For the heterozygous SNPs, the intensity values for each probe were extracted from the CEL files generated. The value for each probe pair was calculated by subtracting the mismatch (MM) intensity from the perfect match (PM) intensity. A *t* test was used to calculate a p-value for the presence of signal for each allele of each SNP (intensity greater than zero = expression detected). The manufacturer defines a mini-block as a group of four probes that include a PM and a MM probe for allele 1, and a PM and a MM probe for allele 2. The Mapping 10 K Array contains ten mini-blocks, 5 of which correspond to the forward strand and the other five to the reverse strand. A signal was considered if at least one allele developed a signal (p<0.01, *t* test) in any of the strands. If a signal was only present in one strand, the allele fraction (the ratio of expression of the two alleles) was calculated only with the mini-blocks of the corresponding strand. Thus, we quantified the ratio of expression of the two alleles for the heterozygous SNPs present in transcripts expressed in PBMCs. In order to obtain a statistical measure, the 99% confidence interval for the allele ratio of gDNA (equivalent to equal expression of the two alleles) was calculated for both alleles using all the heterozygous SNPs in the 20 individuals. We obtained ranges between 0.81 and 1.37. SNPs with differential allelic gene expression were considered if the ratio of allele 1 to allele 2 fell outside of the corresponding confidence interval.

### The distribution of allele specific expression across the genome

DNA-Chip Analyzer 2004 software [27] was used to map SNPs to chromosomes and to look for clusters of differentially expressed alleles of SNPs. To assess the significance of “SNP proximity”, p-values were calculated for all the stretches containing ≤20 SNPs with differential allelic expression. Significant stretches of differentially expressed SNPs were considered when the p-value<0.00005.

[A paragraph has been removed from here]

### Data about Segmental Duplication and Copy Number Variations obtained from public databases

Copy Number Variation (CNV) and Segmental Duplication (SD) data were obtained from publicly available databases and were divided into two categories. The first one, that we called “dbCNV” was obtained at http://projects.tcag.ca/variation/, and the second one called “dbSD” was downloaded from http://eichlerlab.gs.washington.edu/database.html. For dbCNV, only the studies based on large samples obtained from general population were included, to avoid biasing our database towards rare or disease-related variants. All coordinates were from build35 (hg17). On each independent database, we first filtered duplicates out and then concatenated overlapping segments in order to form a list of unique and excluding coordinate pairs representing regions with SDs and/or CNVs. After these changes, the CNV database presented 3,272 regions, distributed all over the genome. The size of the CNV of this database ranged from 7,486,165 bp to 1,032 bp, with a mean size of 193,588 bp, and a standard deviation of 394,728 bp. The SD database after modifications showed 8,096 different duplications, with a size range from 875,877 bp to 999 bp, a mean size of 15,990 bp and a standard deviation of 44,422 bp.

### CNV detection in the samples

To detect CNVs in the samples, two technologies were used. One was the Affymetrix GeneChip® Human Mapping 10 K Array, covering 10,136 SNPs that had been used for the rest of the analysis as explained above. The other one was the Agilent G4410B array, a commercially available 60-mer oligonucleotide microarray for CGH, with probes located in coding and non-coding sequences at an average spatial resolution of 35 kb, and where 44,887 probes were analyzed. For this second array, we hybridized the samples following the manufacturer's protocol (v2), in dye-swap experiments against a reference pool from the same gender. Reference samples consist in a pool of 50 normal individuals from the same gender. In brief, 1000 ng of DNA was digested with 5 units of Alu I and Afa I (GE Healthcare) during 2 hours at 37°. After inactivating the enzymes 20 minutes at 65°C, the DNA was labeled using the Bioprime arrayCGH Labeling kit (Invitrogen). 20 µl of 2.5× Random Primer solution was added and incubated 5 min at 95° followed by 5 min in ice. Then 5 µl of 10×dNTP mix were added as well as 3 µl of 1 mM dUTP-Cy3 or dUTP-Cy5 (GE Healthcare) and 40 U of Klenow fragment (Invitrogen). The reaction was incubated 2 hours at 37° and was cleaned up using Microcons YM-30 (Millipore). 1.5 µl of the labeled DNA was used to check for the incorporation of fluorescent nucleotide incorporation using a Nanodrop instrument. Then test sample and reference were mixed together with 50 µl of 10× Blocking Agent (Agilent), 50 µg of human Cot-1 (Roche) and 250 µl of 2×Hybridization buffer (Agilent). A denaturation step was performed during 3 min at 95° followed by an incubation of 30 min at 37° before hybridization. Arrays were hybridized during 40 at 65° in a hybridization oven rotating at 10 rpm. Arrays were washed 5 min in oligo aCGH wash buffer 1 (Agilent) at RT, 1 min in oligo aCGH wash buffer 2 (Agilent) at 37°, 30 sec in actonitrile (Sigma) at RT and 30 sec in stabilizing and drying solution (Agilent) at RT to prevent ozone degradation. All washes were performed with agitation using a magnetic stir.

Arrays were scanned using an Agilent G2565BA MicroArray Scanner System (Agilent Inc., Palo Alto, Ca) and the acquired images were analyzed using GenePix Pro 6.0 software (Axon, Molecular Devices) using the irregular feature finding option. Extracted raw data was filtered and Loess normalized using Bacanal (Lozano et a., unpublished), an in house web server implementation of the Limma package developed within the Bioconductor project in the R statistical programming environment.

### CNV-detection algorithm

The data were analyzed with the R software [28]. Data obtained from both technologies (10 K and 44 K arrays) were analyzed separately. In both cases, the standard deviation of the mean log2 values for autosomes were calculated for all the individuals and the distribution of these values was plotted. Individuals for which standard deviations were below 0.18 and for which the distribution of the log2 values was symmetrical with respect to 0 were selected for a first analysis. In this first analysis, we wrote a R script that looks for two consecutive clones or 3 out of 4 consecutive clones that would have log2 ratios above a multiple of the standard deviation of the whole individual. Then, the script checks for consistency with dye-swap data, and only keeps the regions that are also called in the dye-swap experiment. This approach is very sensitive to the quality of the data and is not adapted to cases where data are dispersed or if there are local trends in the data. This is why only data of 18 individuals obtained with the 44 K array were analyzed with the first method. R functions designed to deal with these problems were applied to the part of the dataset that did not match the criteria for the first analysis. The second analysis was applied to all the individuals (including those that were analyzed in the first analysis), and was done as follows. First, the normalized log ratios from each dye-swap were averaged before the analysis. Then, a denoising step was applied using the method described in Hsu et al. [29] using the Haar wavelet family and the sure estimator for thresholding, with Jo (the level up to which the wavelet coefficients are subject to thresholding) equals to 4. The wavelet decomposition and reconstruction functions were from the WAVESLIM package. Finally, the Circular Binary Segmentation algorithm described in Olshen et al [30] was used for clone calling. For this purpose, the functions CNA, smooth. CNA and segment, available in the DNAcopy package, were used with default parameter values. Data from the two analyses were combined. The second analysis method is, overall, more conservative, but has the ability to rescue some regions that would not pass the very strict threshold based on standard deviation. Consequently, the overlap between the regions called with the two methods is large, but not complete. This is why the clones that were called by only one method were manually checked in the data file for validity of signal. At the end of the BAC call process, regions of more than 1 Mb were removed from the list. The final list was constituted by 294 calls (an average of 14.7 per individual). The data obtained through this process are individual CNV coordinates and we subsequently refer to them as “indCNV”.

### Allele-specific gene expression analysis

The most characteristic GO term for each cluster was Assigned using FatiGO [31]. The list of imprinted genes was obtained from the Genomic Imprinting Database (http://www.geneimprint.com/).

### Real-time quantitative PCR validation

Quantitative real-time PCR analysis was performed with a DNA Engine Opticon2 (MJ Research). Primer sequences and target-specific fluorescent labeled TaqMan probes used for both genotyping and allele-specific gene expression were purchased from Applied Biosystems (TaqMan SNP Genotyping Assays). PCR reactions were prepared following the manufacturer's protocol. Genotype calls were acquired with Opticon Monitor 2.01 software (MJ Research) and allele-specific gene expression was measured as described previously [9]. In short, 48 individuals were genotyped for each SNP by real time PCR. We selected eight heterozygous individuals for each SNP for allelic to validate expression. Allele-specific gene expression was measured in these 8 individuals by real time PCR. A standard curve (linear regression line) was generated for each SNP mixing gDNAs from two homozygous individuals at ratios 8∶1, 4∶1, 2∶1, 1∶1, 1∶2, 1∶4 and 1∶8, one for each genotype. To check that standard curves were generated with truly homozygous individuals four of them were sequenced for three SNPs confirming the homozygosis. Each sample was run in triplicate, and cycle threshold (c(t)) values were obtained with Opticon Monitor 2.01 software. Using this information we subtracted the baseline signal as the lowest fluorescent signal measured, and we set the c(t) line to a standard deviation of 0.1. The log of FAM mean c(t)/VIC mean c(t) values were plotted against the log of the gDNA ratio. The linear graphs obtained (correlation coefficients >0.9) were used to calculate the corresponding allele-specific gene expression. The 99% confidence interval for the allele-specific gene expression (equivalent to equal expression) was generated from heterozygous DNAs. SNPs with allele-specific gene expression outside of the corresponding confidence interval were considered significant.

### Statistical tests

On all the datasets (dbCNV, SD and indCNV), we tested the association between SNPs with significant ASGE and proximity of a CNV by the means of Chi-square tests. To this purpose, we wrote a series of PHP scripts to check whether each SNP was located within a SD/CNV, between 1 bp and 10 Kb upstream or downstream a SD/CNV, between 10 Kb and 100 Kb upstream or downstream a SD/CNV or between 100 Kb and 1 Mb upstream or downstream a SD/CNV. When a SNP could belong to two different SD/CNVs, we always exclusively considered the shortest distance. No SNP could therefore belong to two SDs or two CNVs, or could be included in two different categories of distance in the same SDs or CNVs.

For data obtained from public databases, we crossed the positions of the dbSD and dbCNVwith those of the 5,133 SNP that were heterozygous in at least one individual. For the individual data, instead of using the 5,133 SNP, we used only the ones that were heterozygous in the individual we were testing, and instead of using public database, we used the results of the 10 K and 44 K hybridization data. That is, we crossed the individual information of indCNV for a given individual and its own heterozygous SNPs. In this analysis, we therefore generated 20 tables for each of the distance windows we considered. Because very few indCNV were detected in the individual analysis, each contingency table had small sample sizes. To overcome this problem, we performed single Chi-square tests on synthetic tables built for each distance window, in which numbers in each cell represented the cumulate sample size of the 20 individuals for the corresponding category, previously obtained in the 20 individual tables. In the analysis of indCNV, in order to overcome problems of sample size, we also considered a window of distance that would include all SNP from inside up to 100 K upstream and downstream of a CNV.

In addition to Chi-square tests, we also performed permutation tests for each window of size, to assess whether the ratio of allele expression was the same inside or outside the distance considered.

## Supporting Information

Table S1 (0.79 MB TXT)

Table S2 (0.09 MB PDF)

Table S3 (0.01 MB TXT)
