# Supplementary material for: Allele-Specific Gene Expression Is Widespread Across the Genome and Biological Processes
Source: PLoS One. 2009 Jan 7;4(1):e4150. doi: 10.1371/journal.pone.0004150 (PMC2613524; doi:10.1371/journal.pone.0004150)
Supplement: Table S2 — (0.09 MB PDF) [file pone.0004150.s002.pdf]

| Gene Ontology : biological process. Level: 3 | Percentage of genes with allele-specific expression | Percentage of genes without allele-specific expression | Unadjusted pvalue | Adjusted pvalueFDR |
|---|---|---|---|---|
| neurological process | 7.17 | 12.1 | 1.73E-03 | 1 |
| tissue remodeling | 0.56 | 2.38 | 2.30E-03 | 1 |
| primary metabolic process | 54.74 | 48.16 | 1.65E-02 | 1 |
| macromolecule metabolic process | 47.57 | 41.25 | 2.19E-02 | 1 |
| response to abiotic stimulus | 0.64 | 1.94 | 2.45E-02 | 1 |
| cell communication | 32.83 | 38.66 | 2.56E-02 | 1 |
| behavior | 3.27 | 5.4 | 4.75E-02 | 1 |
| cellular metabolic process | 55.86 | 50.76 | 6.35E-02 | 1 |
| digestion | 0.32 | 1.08 | 6.52E-02 | 1 |
| circulation | 1.04 | 2.16 | 9.48E-02 | 1 |
| detection of stimulus | 0.4 | 1.08 | 1.45E-01 | 1 |
| developmental growth | 0.48 | 0 | 2.00E-01 | 1 |
| molting cycle | 0.56 | 0 | 2.00E-01 | 1 |
| endocrine process | 0 | 0.22 | 2.69E-01 | 1 |
| sleep | 0 | 0.22 | 2.69E-01 | 1 |
| multicellular organismal homeostasis | 0.16 | 0.43 | 2.95E-01 | 1 |
| organismal movement | 0.16 | 0.43 | 2.95E-01 | 1 |
| death | 6.22 | 4.97 | 3.57E-01 | 1 |
| catabolic process | 4.3 | 5.4 | 3.63E-01 | 1 |
| regulation of a molecular function | 3.19 | 4.1 | 3.71E-01 | 1 |
| coagulation | 0.96 | 0.43 | 3.76E-01 | 1 |
| aging | 0.32 | 0.65 | 3.95E-01 | 1 |
| response to stress | 7.81 | 6.48 | 4.08E-01 | 1 |
| regulation of biological quality | 2.07 | 1.3 | 4.20E-01 | 1 |
| localization of cell | 5.18 | 4.1 | 4.49E-01 | 1 |
| thermoregulation | 0.08 | 0.22 | 4.66E-01 | 1 |
| organ growth | 0.08 | 0.22 | 4.66E-01 | 1 |
| cellular developmental process | 16.57 | 15.12 | 5.07E-01 | 1 |
| autophagy | 0.24 | 0 | 5.68E-01 | 1 |
| homeostasis of number of cells | 0.24 | 0 | 5.68E-01 | 1 |
| body growth | 0.24 | 0 | 5.68E-01 | 1 |
| interspecies interaction between organisms | 0.24 | 0 | 5.68E-01 | 1 |
| cell organization and biogenesis | 18.41 | 17.06 | 5.72E-01 | 1 |
| defense response | 4.38 | 3.67 | 5.88E-01 | 1 |
| cell proliferation | 6.85 | 7.56 | 5.97E-01 | 1 |

| | | | | |
|---|---|---|---|---|
| response to external stimulus | 5.26 | 4.54 | 6.20E-01 | 1 |
| muscle contraction | 1.35 | 1.73 | 6.50E-01 | 1 |
| response to chemical stimulus | 3.27 | 3.67 | 6.54E-01 | 1 |
| regulation of biological process | 35.86 | 34.77 | 0.6912833 | 1 |
| response to endogenous stimulus | 2.31 | 1.94 | 0.7156244 | 1 |
| cell activation | 2.39 | 1.94 | 0.7157399 | 1 |
| response to biotic stimulus | 2.31 | 2.59 | 0.7234653 | 1 |
| synapse organization and biogenesis | 0.56 | 0.65 | 0.7345141 | 1 |
| multicellular organismal development | 22.47 | 21.6 | 0.7438192 | 1 |
| establishment of localization | 20.96 | 20.3 | 0.7890163 | 1 |
| developmental maturation | 1.12 | 0.86 | 0.7934371 | 1 |
| cell homeostasis | 1.99 | 1.73 | 0.8442724 | 1 |
| chemical homeostasis | 2.07 | 2.16 | 0.8518755 | 1 |
| protein localization | 6.29 | 6.05 | 0.9107316 | 1 |
| cell adhesion | 7.65 | 7.78 | 0.918937 | 1 |
| anatomical structure development | 21.35 | 21.6 | 0.9471706 | 1 |
| RNA localization | 0.32 | 0.22 | 1 | 1 |
| maintenance of localization | 0.16 | 0 | 1 | 1 |
| behavioral interaction between organisms | 0.4 | 0.22 | 1 | 1 |
| cell division | 1.67 | 1.51 | 1 | 1 |
| reproductive process | 1.67 | 1.51 | 1 | 1 |
| biosynthetic process | 9 | 8.86 | 1 | 1 |
| regulation of body fluids | 1.04 | 0.86 | 1 | 1 |
| cell recognition | 0.48 | 0.43 | 1 | 1 |
| nitrogen compound metabolic process | 2.79 | 2.59 | 1 | 1 |
| secondary metabolic process | 0.24 | 0.22 | 1 | 1 |
| respiratory gaseous exchange | 0.32 | 0.22 | 1 | 1 |
| cytokine production | 1.12 | 1.08 | 1 | 1 |
| pigmentation during development | 0.16 | 0.22 | 1 | 1 |
| cell cycle | 7.09 | 6.91 | 1 | 1 |
| membrane docking | 0.4 | 0.22 | 1 | 1 |
| sexual reproduction | 1.99 | 1.94 | 1 | 1 |
| chromosome segregation | 0.4 | 0.43 | 1 | 1 |
| extracellular matrix organization and biogenesis | 0.64 | 0.43 | 1 | 1 |
| circadian rhythm | 0.24 | 0.22 | 1 | 1 |
| excretion | 0.32 | 0.22 | 1 | 1 |

| | | | | |
|---|---|---|---|---|
| immune system process | 7.17 | 7.13 | 1 | 1 |

| Gene Ontology : biological process. Level: 4 | Percentage of genes with allele-specific expression | Percentage of genes without allele-specific expression | Unadjusted pvalue | Adjusted pvalueFDR |
|---|---|---|---|---|
| protein metabolic process | 27.54 | 20.88 | 0.005665717 | 1 |
| sensory perception | 3.85 | 6.81 | 0.01311375 | 1 |
| cellular macromolecule metabolic process | 26.15 | 20.44 | 0.01545811 | 1 |
| bone remodeling | 0.57 | 1.98 | 0.01897887 | 1 |
| biopolymer metabolic process | 38.36 | 32.53 | 0.03037133 | 1 |
| adult behavior | 0.25 | 1.1 | 0.03826845 | 1 |
| phosphorus metabolic process | 9.43 | 6.15 | 0.03852618 | 1 |
| detection of external stimulus | 0.16 | 0.88 | 0.04988037 | 1 |
| positive regulation of biological process | 11.31 | 8.13 | 0.05967504 | 1 |
| detection of abiotic stimulus | 0.08 | 0.66 | 0.06361406 | 1 |
| glucose homeostasis | 0.08 | 0.66 | 0.06361406 | 1 |
| rhythmic behavior | 0 | 0.44 | 0.07367107 | 1 |
| regulation of binding | 0 | 0.44 | 0.07367107 | 1 |
| response to radiation | 0.41 | 1.32 | 0.07998575 | 1 |
| regulation of locomotion | 0.49 | 1.32 | 0.09926941 | 1 |
| learning and/or memory | 0.82 | 1.76 | 0.111236 | 1 |
| response to organic substance | 0.16 | 0.66 | 0.1273231 | 1 |
| cell aging | 0.16 | 0.66 | 0.1273231 | 1 |
| signal transduction | 30.25 | 34.07 | 0.1388143 | 1 |
| regulation of growth | 1.64 | 0.66 | 0.1583784 | 1 |
| response to wounding | 3.93 | 2.42 | 0.1787818 | 1 |
| regulation of blood vessel size | 0.08 | 0.44 | 0.1811172 | 1 |
| smooth muscle contraction | 0.08 | 0.44 | 0.1811172 | 1 |
| drug metabolic process | 0.08 | 0.44 | 0.1811172 | 1 |
| hair cycle | 0.57 | 0 | 0.2000642 | 1 |
| molting cycle process | 0.57 | 0 | 0.2000642 | 1 |
| regulation of multicellular organismal process | 2.87 | 4.18 | 0.2123753 | 1 |
| embryonic development | 3.69 | 2.42 | 0.2238763 | 1 |
| cell-cell signaling | 7.05 | 8.79 | 0.2517451 | 1 |
| cellular catabolic process | 3.77 | 5.05 | 0.2683857 | 1 |
| response to gravity | 0 | 0.22 | 0.2716418 | 1 |
| homoiothermy | 0 | 0.22 | 0.2716418 | 1 |
| response to mechanical stimulus | 0 | 0.22 | 0.2716418 | 1 |
| chemosensory behavior | 0 | 0.22 | 0.2716418 | 1 |
| striated muscle contraction | 0.16 | 0.44 | 0.2986204 | 1 |

| | | | | |
|---|---|---|---|---|
| glycerol ether metabolic process | 0.16 | 0.44 | 0.2986204 | 1 |
| cytokinesis | 0.16 | 0.44 | 0.2986204 | 1 |
| tissue homeostasis | 0.16 | 0.44 | 0.2986204 | 1 |
| antigen processing and presentation | 0.41 | 0 | 0.3318527 | 1 |
| interleukin-6 production | 0.41 | 0 | 0.3318527 | 1 |
| response to drug | 0.25 | 0.66 | 0.3535703 | 1 |
| alkene metabolic process | 0.25 | 0.66 | 0.3535703 | 1 |
| locomotory behavior | 2.13 | 2.86 | 0.367473 | 1 |
| cell motility | 5.33 | 4.18 | 0.3797009 | 1 |
| organelle organization and biogenesis | 9.34 | 7.91 | 0.3878559 | 1 |
| macromolecule catabolic process | 2.7 | 3.52 | 0.4150318 | 1 |
| response to other organism | 1.72 | 2.42 | 0.4213928 | 1 |
| nucleobase, nucleoside, nucleotide and nucleic acid met | 25.25 | 23.3 | 0.4448913 | 1 |
| response to protein stimulus | 0.66 | 0.22 | 0.4584797 | 1 |
| cell differentiation | 17.05 | 15.38 | 0.4604103 | 1 |
| alcohol metabolic process | 2.54 | 1.76 | 0.4659934 | 1 |
| interferon-gamma production | 0.08 | 0.22 | 0.4696125 | 1 |
| detection of chemical stimulus | 0.08 | 0.22 | 0.4696125 | 1 |
| response to temperature stimulus | 0.08 | 0.22 | 0.4696125 | 1 |
| response to pain | 0.08 | 0.22 | 0.4696125 | 1 |
| musculoskeletal movement | 0.08 | 0.22 | 0.4696125 | 1 |
| sulfur metabolic process | 0.49 | 0.88 | 0.4741691 | 1 |
| anatomical structure morphogenesis | 11.48 | 10.11 | 0.4843789 | 1 |
| oxygen and reactive oxygen species metabolic process | 0.57 | 0.88 | 0.5027782 | 1 |
| reproductive developmental process | 0.57 | 0.88 | 0.5027782 | 1 |
| cofactor metabolic process | 1.64 | 1.1 | 0.5031182 | 1 |
| immune response | 4.51 | 5.27 | 0.5180336 | 1 |
| appendage development | 0.82 | 0.44 | 0.5305785 | 1 |
| pattern specification process | 1.89 | 1.32 | 0.5307622 | 1 |
| negative regulation of biological process | 11.89 | 10.77 | 0.5491735 | 1 |
| regulation of cellular process | 31.97 | 30.33 | 0.5544919 | 1 |
| regulation of coagulation | 0.25 | 0 | 0.5672571 | 1 |
| response to hormone stimulus | 0.25 | 0 | 0.5672571 | 1 |
| symbiosis, encompassing mutualism through parasitism | 0.25 | 0 | 0.5672571 | 1 |
| aldehyde metabolic process | 0.25 | 0 | 0.5672571 | 1 |
| leukocyte homeostasis | 0.25 | 0 | 0.5672571 | 1 |

| | | | | |
|---|---|---|---|---|
| regulation of metabolic process | 18.77 | 20 | 0.5765539 | 1 |
| response to extracellular stimulus | 0.33 | 0 | 0.5796803 | 1 |
| regulation of response to stimulus | 0.33 | 0 | 0.5796803 | 1 |
| epithelial cell proliferation | 0.25 | 0.44 | 0.6173481 | 1 |
| interleukin-2 production | 0.25 | 0.44 | 0.6173481 | 1 |
| fear response | 0.25 | 0.44 | 0.6173481 | 1 |
| aromatic compound metabolic process | 1.56 | 1.1 | 0.6450092 | 1 |
| regulation of catalytic activity | 3.28 | 3.74 | 0.6506001 | 1 |
| response to xenobiotic stimulus | 0.33 | 0.44 | 0.6656493 | 1 |
| organic acid metabolic process | 3.69 | 4.18 | 0.6677004 | 1 |
| gametogenesis | 1.97 | 1.54 | 0.6855553 | 1 |
| response to DNA damage stimulus | 2.13 | 1.76 | 0.7016256 | 1 |
| leukocyte activation | 2.38 | 1.98 | 0.7149152 | 1 |
| heterocycle metabolic process | 0.74 | 0.44 | 0.7371745 | 1 |
| vitamin metabolic process | 0.74 | 0.44 | 0.7371745 | 1 |
| cell cycle process | 6.31 | 6.81 | 0.7373835 | 1 |
| hormone metabolic process | 0.66 | 0.88 | 0.744756 | 1 |
| one-carbon compound metabolic process | 0.66 | 0.88 | 0.744756 | 1 |
| regulation of developmental process | 3.28 | 2.86 | 0.7547742 | 1 |
| hemostasis | 0.98 | 0.66 | 0.771524 | 1 |
| lipid metabolic process | 5.25 | 5.49 | 0.8078098 | 1 |
| cellular localization | 5.41 | 5.71 | 0.8099349 | 1 |
| tube development | 1.39 | 1.1 | 0.8106824 | 1 |
| ion homeostasis | 1.89 | 1.54 | 0.8360171 | 1 |
| transport | 20.82 | 20.22 | 0.8387658 | 1 |
| amino acid and derivative metabolic process | 2.21 | 1.98 | 0.8518027 | 1 |
| membrane organization and biogenesis | 2.54 | 2.64 | 0.8638721 | 1 |
| cell-cell adhesion | 2.95 | 3.08 | 0.8726646 | 1 |
| carbohydrate metabolic process | 3.61 | 3.3 | 0.8817467 | 1 |
| macromolecule complex assembly | 3.77 | 3.96 | 0.8862178 | 1 |
| system development | 19.1 | 18.68 | 0.8886906 | 1 |
| mitotic cell cycle | 1.89 | 1.76 | 1 | 1 |
| response to toxin | 0.08 | 0 | 1 | 1 |
| response to inorganic substance | 0.08 | 0 | 1 | 1 |
| maintenance of protein localization | 0.08 | 0 | 1 | 1 |
| cellular biosynthetic process | 8.28 | 8.35 | 1 | 1 |

| | | | | |
|---|---|---|---|---|
| feeding behavior | 0.41 | 0.44 | 1 | 1 |
| stem cell division | 0.08 | 0 | 1 | 1 |
| interleukin-8 production | 0.08 | 0 | 1 | 1 |
| interleukin-13 production | 0.08 | 0 | 1 | 1 |
| generation of precursor metabolites and energy | 3.61 | 3.52 | 1 | 1 |
| cellular structure disassembly | 0.25 | 0.22 | 1 | 1 |
| fertilization | 0.16 | 0.22 | 1 | 1 |
| cell-cell recognition | 0.08 | 0 | 1 | 1 |
| interleukin-1 production | 0.08 | 0 | 1 | 1 |
| viral reproductive process | 0.41 | 0.22 | 1 | 1 |
| reproductive process in a multicellular organism | 0.57 | 0.44 | 1 | 1 |
| chemokine production | 0.08 | 0 | 1 | 1 |
| pigment metabolic process | 0.16 | 0 | 1 | 1 |
| nitric oxide metabolic process | 0.16 | 0.22 | 1 | 1 |
| smooth muscle cell proliferation | 0.08 | 0 | 1 | 1 |
| response to tumor cell | 0.08 | 0 | 1 | 1 |
| cytoplasm organization and biogenesis | 0.16 | 0.22 | 1 | 1 |
| amine metabolic process | 2.7 | 2.64 | 1 | 1 |
| urea cycle intermediate metabolic process | 0.08 | 0 | 1 | 1 |
| secretion | 2.95 | 2.86 | 1 | 1 |
| establishment of protein localization | 6.07 | 5.93 | 1 | 1 |
| collagen fibril organization | 0.16 | 0.22 | 1 | 1 |
| cholesterol homeostasis | 0.08 | 0 | 1 | 1 |
| response to osmotic stress | 0.16 | 0 | 1 | 1 |
| blood pressure regulation | 0.25 | 0.22 | 1 | 1 |
| endothelial cell proliferation | 0.16 | 0.22 | 1 | 1 |
| keratinocyte proliferation | 0.08 | 0 | 1 | 1 |
| response to hypoxia | 0.08 | 0 | 1 | 1 |
| reproductive behavior | 0.33 | 0.22 | 1 | 1 |
| interleukin-12 production | 0.16 | 0.22 | 1 | 1 |
| cellular defense response | 0.33 | 0.22 | 1 | 1 |
| cell redox homeostasis | 0.08 | 0 | 1 | 1 |
| neuromuscular process | 0.16 | 0.22 | 1 | 1 |
| regulation of cytokine production | 0.08 | 0 | 1 | 1 |
| peptide metabolic process | 0.16 | 0.22 | 1 | 1 |
| sequestering of metal ion | 0.08 | 0 | 1 | 1 |

| | | | | |
|---|---|---|---|---|
| macroautophagy | 0.08 | 0 | 1 | 1 |
| social behavior | 0.08 | 0 | 1 | 1 |
| meiotic cell cycle | 0.57 | 0.44 | 1 | 1 |
| establishment of RNA localization | 0.33 | 0.22 | 1 | 1 |
| detection of biotic stimulus | 0.16 | 0.22 | 1 | 1 |
| ribonucleoprotein complex biogenesis and assembly | 0.82 | 0.88 | 1 | 1 |
| interleukin-4 production | 0.08 | 0 | 1 | 1 |
| osmoregulation | 0.08 | 0 | 1 | 1 |
| fibroblast proliferation | 0.16 | 0.22 | 1 | 1 |
| detection of endogenous stimulus | 0.08 | 0 | 1 | 1 |
| neuron recognition | 0.25 | 0.22 | 1 | 1 |
| granulocyte macrophage colony-stimulating factor produ | 0.16 | 0 | 1 | 1 |
| cell-substrate adhesion | 0.41 | 0.44 | 1 | 1 |
| regulation of gene expression, epigenetic | 0.33 | 0.22 | 1 | 1 |
| asymmetric cell division | 0.16 | 0 | 1 | 1 |
| tumor necrosis factor-beta production | 0.16 | 0 | 1 | 1 |
| metabolic compound salvage | 0.16 | 0 | 1 | 1 |
| entrainment of circadian clock | 0.08 | 0 | 1 | 1 |
| interleukin-3 production | 0.08 | 0 | 1 | 1 |
| grooming behavior | 0.08 | 0 | 1 | 1 |
| external encapsulating structure organization and bioger | 0.16 | 0 | 1 | 1 |
| behavioral defense response | 0.16 | 0.22 | 1 | 1 |

| Gene Ontology : biological process. Level: 5 | Percentage of genes with allele-specific expression | Percentage of genes without allele-specific expression | Unadjusted pvalue | Adjusted pvalueFDR |
|---|---|---|---|---|
| biopolymer modification | 17.88 | 11.14 | 0.000946105 | 1 |
| sensory perception of chemical stimulus | 0.94 | 3.02 | 0.004519434 | 1 |
| cell surface receptor linked signal transduction | 13.34 | 18.79 | 0.008701017 | 1 |
| cellular protein metabolic process | 26.95 | 20.88 | 0.01376415 | 1 |
| regulation of bone remodeling | 0 | 0.7 | 0.01944723 | 1 |
| phosphate metabolic process | 9.84 | 6.5 | 0.03816538 | 1 |
| sensory perception of light stimulus | 1.54 | 3.02 | 0.06615464 | 1 |
| response to amphetamine | 0 | 0.46 | 0.07243981 | 1 |
| vasoconstriction | 0 | 0.46 | 0.07243981 | 1 |
| positive regulation of cellular process | 9.32 | 6.5 | 0.08640517 | 1 |
| polysaccharide metabolic process | 0.26 | 0.93 | 0.08936988 | 1 |
| transmission of nerve impulse | 4.11 | 6.03 | 0.1083607 | 1 |
| regulation of cell adhesion | 0.68 | 0 | 0.1176141 | 1 |
| nitrogen compound biosynthetic process | 0.6 | 1.39 | 0.1246316 | 1 |
| female gamete generation | 0.17 | 0.7 | 0.1246602 | 1 |
| sulfur compound biosynthetic process | 0.17 | 0.7 | 0.1246602 | 1 |
| regulation of transferase activity | 1.97 | 3.25 | 0.1359861 | 1 |
| urogenital system development | 0.68 | 1.62 | 0.1379155 | 1 |
| response to light stimulus | 0.43 | 1.16 | 0.1450318 | 1 |
| negative regulation of multicellular organismal process | 0.09 | 0.46 | 0.178425 | 1 |
| detection of stimulus during sensory perception | 0.09 | 0.46 | 0.178425 | 1 |
| regulation of cell motility | 0.51 | 1.16 | 0.1786355 | 1 |
| response to bacterium | 0.51 | 1.16 | 0.1786355 | 1 |
| cell fate commitment | 0.86 | 1.62 | 0.1802032 | 1 |
| embryonic development (sensu Metazoa) | 1.37 | 0.46 | 0.1812811 | 1 |
| biopolymer catabolic process | 2.05 | 3.25 | 0.1938639 | 1 |
| hair cycle process | 0.6 | 0 | 0.1999694 | 1 |
| water-soluble vitamin metabolic process | 0.6 | 0 | 0.1999694 | 1 |
| nucleoside metabolic process | 0.51 | 0 | 0.2000239 | 1 |
| nuclear organization and biogenesis | 0.51 | 0 | 0.2000239 | 1 |
| intracellular signaling cascade | 15.4 | 12.76 | 0.2030225 | 1 |
| inflammatory response | 2.82 | 1.62 | 0.2079993 | 1 |
| cellular macromolecule catabolic process | 2.48 | 3.71 | 0.2314653 | 1 |
| positive regulation of metabolic process | 4.11 | 2.78 | 0.2386537 | 1 |
| ion transport | 7.44 | 9.28 | 0.2512664 | 1 |

| | | | | |
|---|---|---|---|---|
| cofactor biosynthetic process | 1.2 | 0.46 | 0.2619532 | 1 |
| fluid secretion | 0 | 0.23 | 0.269375 | 1 |
| cocaine metabolic process | 0 | 0.23 | 0.269375 | 1 |
| mechanosensory behavior | 0 | 0.23 | 0.269375 | 1 |
| response to alkaloid | 0 | 0.23 | 0.269375 | 1 |
| response to carbohydrate stimulus | 0 | 0.23 | 0.269375 | 1 |
| endothelial cell differentiation | 0 | 0.23 | 0.269375 | 1 |
| fast regulation of arterial pressure | 0 | 0.23 | 0.269375 | 1 |
| sequestering of lipid | 0 | 0.23 | 0.269375 | 1 |
| antibiotic metabolic process | 0 | 0.23 | 0.269375 | 1 |
| retinal homeostasis | 0 | 0.23 | 0.269375 | 1 |
| urothelial cell proliferation | 0 | 0.23 | 0.269375 | 1 |
| detection of gravity | 0 | 0.23 | 0.269375 | 1 |
| microtubule organizing center organization and biogenes | 0 | 0.23 | 0.269375 | 1 |
| response to ethanol | 0 | 0.23 | 0.269375 | 1 |
| dosage compensation | 0 | 0.23 | 0.269375 | 1 |
| organic acid catabolic process | 0 | 0.23 | 0.269375 | 1 |
| regulation of posture | 0 | 0.23 | 0.269375 | 1 |
| exocrine system development | 0 | 0.23 | 0.269375 | 1 |
| eating behavior | 0 | 0.23 | 0.269375 | 1 |
| parturition | 0 | 0.23 | 0.269375 | 1 |
| circadian behavior | 0 | 0.23 | 0.269375 | 1 |
| regulation of DNA binding | 0 | 0.23 | 0.269375 | 1 |
| nervous system development | 10.01 | 8.12 | 0.290489 | 1 |
| cellular lipid metabolic process | 4.45 | 5.8 | 0.291837 | 1 |
| response to fungus | 0.17 | 0.46 | 0.2946967 | 1 |
| fat-soluble vitamin metabolic process | 0.17 | 0.46 | 0.2946967 | 1 |
| mesenchymal cell differentiation | 0.17 | 0.46 | 0.2946967 | 1 |
| photoreceptor cell differentiation | 0.17 | 0.46 | 0.2946967 | 1 |
| hormone biosynthetic process | 0.17 | 0.46 | 0.2946967 | 1 |
| calcium-dependent cell-cell adhesion | 0.17 | 0.46 | 0.2946967 | 1 |
| regulation of catabolic process | 0.17 | 0.46 | 0.2946967 | 1 |
| alcohol catabolic process | 0.77 | 0.23 | 0.3044141 | 1 |
| cytoskeleton organization and biogenesis | 5.73 | 4.41 | 0.3204322 | 1 |
| regulation of heart contraction | 0.6 | 1.16 | 0.3239519 | 1 |
| regulation of cyclase activity | 0.43 | 0 | 0.3323761 | 1 |

| | | | | |
|---|---|---|---|---|
| negative regulation of growth | 0.43 | 0 | 0.3323761 | 1 |
| regulation of lyase activity | 0.43 | 0 | 0.3323761 | 1 |
| protein secretion | 0.43 | 0 | 0.3323761 | 1 |
| DNA damage response, signal transduction | 0.43 | 0 | 0.3323761 | 1 |
| mitochondrion organization and biogenesis | 0.43 | 0 | 0.3323761 | 1 |
| regulation of signal transduction | 4.96 | 3.71 | 0.3479831 | 1 |
| response to oxidative stress | 0.26 | 0.7 | 0.3524705 | 1 |
| adult locomotory behavior | 0.26 | 0.7 | 0.3524705 | 1 |
| lipid catabolic process | 0.68 | 1.16 | 0.3534935 | 1 |
| regulation of immune system process | 1.03 | 0.46 | 0.3755928 | 1 |
| reproductive structure development | 0.34 | 0.7 | 0.3951182 | 1 |
| cell morphogenesis | 6.07 | 4.87 | 0.3984851 | 1 |
| membrane invagination | 2.14 | 1.39 | 0.4167417 | 1 |
| organ development | 13.94 | 15.55 | 0.4224799 | 1 |
| regionalization | 1.28 | 0.7 | 0.4287577 | 1 |
| sensory perception of mechanical stimulus | 1.28 | 0.7 | 0.4287577 | 1 |
| cell development | 12.4 | 10.9 | 0.4363132 | 1 |
| heterophilic cell adhesion | 0.43 | 0.7 | 0.4508248 | 1 |
| amine transport | 0.68 | 0.23 | 0.4585975 | 1 |
| regulation of secretion | 0.68 | 0.23 | 0.4585975 | 1 |
| response to unfolded protein | 0.68 | 0.23 | 0.4585975 | 1 |
| endosome organization and biogenesis | 0.09 | 0.23 | 0.4663102 | 1 |
| Golgi organization and biogenesis | 0.09 | 0.23 | 0.4663102 | 1 |
| vesicle organization and biogenesis | 0.09 | 0.23 | 0.4663102 | 1 |
| superoxide metabolic process | 0.09 | 0.23 | 0.4663102 | 1 |
| aromatic compound biosynthetic process | 0.09 | 0.23 | 0.4663102 | 1 |
| bone resorption | 0.09 | 0.23 | 0.4663102 | 1 |
| digestive process | 0.09 | 0.23 | 0.4663102 | 1 |
| regulation of balance | 0.09 | 0.23 | 0.4663102 | 1 |
| detection of calcium ion | 0.09 | 0.23 | 0.4663102 | 1 |
| cell activation during immune response | 0.09 | 0.23 | 0.4663102 | 1 |
| negative regulation of locomotion | 0.09 | 0.23 | 0.4663102 | 1 |
| calcium-independent cell-cell adhesion | 0.09 | 0.23 | 0.4663102 | 1 |
| response to cold | 0.09 | 0.23 | 0.4663102 | 1 |
| regulation of oxidoreductase activity | 0.09 | 0.23 | 0.4663102 | 1 |
| glycerol ether biosynthetic process | 0.09 | 0.23 | 0.4663102 | 1 |

| | | | | |
|---|---|---|---|---|
| acid secretion | 0.09 | 0.23 | 0.4663102 | 1 |
| regulation of carbohydrate metabolic process | 0.09 | 0.23 | 0.4663102 | 1 |
| fertilization (sensu Metazoa) | 0.09 | 0.23 | 0.4663102 | 1 |
| pteridine and derivative metabolic process | 0.09 | 0.23 | 0.4663102 | 1 |
| sex determination | 0.09 | 0.23 | 0.4663102 | 1 |
| terpene metabolic process | 0.09 | 0.23 | 0.4663102 | 1 |
| sensory perception of temperature stimulus | 0.09 | 0.23 | 0.4663102 | 1 |
| localization within membrane | 0.09 | 0.23 | 0.4663102 | 1 |
| coenzyme metabolic process | 1.54 | 0.93 | 0.4705845 | 1 |
| sex differentiation | 0.6 | 0.93 | 0.5001345 | 1 |
| protein-DNA complex assembly | 0.6 | 0.93 | 0.5001345 | 1 |
| morphogenesis of a branching structure | 0.6 | 0.93 | 0.5001345 | 1 |
| learning | 0.6 | 0.93 | 0.5001345 | 1 |
| regulation of anatomical structure morphogenesis | 0.6 | 0.93 | 0.5001345 | 1 |
| lipid transport | 0.86 | 0.46 | 0.5316294 | 1 |
| appendage morphogenesis | 0.86 | 0.46 | 0.5316294 | 1 |
| embryonic pattern specification | 0.86 | 0.46 | 0.5316294 | 1 |
| nucleotide metabolic process | 2.05 | 1.39 | 0.5331728 | 1 |
| lymphocyte activation | 2.22 | 1.62 | 0.5545998 | 1 |
| negative regulation of developmental process | 0.86 | 1.16 | 0.5650768 | 1 |
| immune effector process | 0.86 | 1.16 | 0.5650768 | 1 |
| regulation of transport | 0.86 | 1.16 | 0.5650768 | 1 |
| regulation of cell activation | 0.86 | 1.16 | 0.5650768 | 1 |
| regulation of cellular metabolic process | 18.91 | 20.19 | 0.5679732 | 1 |
| regulation of body size | 0.26 | 0 | 0.5681721 | 1 |
| negative regulation of coagulation | 0.26 | 0 | 0.5681721 | 1 |
| nucleobase metabolic process | 0.26 | 0 | 0.5681721 | 1 |
| axis specification | 0.26 | 0 | 0.5681721 | 1 |
| alcohol biosynthetic process | 0.26 | 0 | 0.5681721 | 1 |
| regulation of defense response | 0.26 | 0 | 0.5681721 | 1 |
| gene silencing | 0.26 | 0 | 0.5681721 | 1 |
| interaction with host | 0.26 | 0 | 0.5681721 | 1 |
| lactation | 0.26 | 0 | 0.5681721 | 1 |
| negative regulation of metabolic process | 4.19 | 3.48 | 0.5682617 | 1 |
| cell migration | 4.28 | 3.48 | 0.5684334 | 1 |
| RNA metabolic process | 19.76 | 18.33 | 0.5685241 | 1 |

| | | | | |
|---|---|---|---|---|
| response to nutrient levels | 0.34 | 0 | 0.5792585 | 1 |
| vitamin biosynthetic process | 0.34 | 0 | 0.5792585 | 1 |
| oligosaccharide metabolic process | 0.34 | 0 | 0.5792585 | 1 |
| cell wall catabolic process | 0.34 | 0 | 0.5792585 | 1 |
| immune response-regulating signal transduction | 0.34 | 0 | 0.5792585 | 1 |
| secretory pathway | 2.31 | 2.78 | 0.5856398 | 1 |
| negative regulation of cellular process | 11.46 | 10.44 | 0.5928768 | 1 |
| chromosome organization and biogenesis | 2.74 | 2.09 | 0.5930421 | 1 |
| peptide transport | 0.26 | 0.46 | 0.6157702 | 1 |
| regulation of muscle contraction | 0.26 | 0.46 | 0.6157702 | 1 |
| vacuole organization and biogenesis | 0.26 | 0.46 | 0.6157702 | 1 |
| wound healing | 1.37 | 0.93 | 0.6163635 | 1 |
| regulation of cell proliferation | 5.22 | 5.8 | 0.6196558 | 1 |
| regulation of cell cycle | 5.39 | 6.03 | 0.6236839 | 1 |
| plasma membrane organization and biogenesis | 0.34 | 0.46 | 0.6632268 | 1 |
| xenobiotic metabolic process | 0.34 | 0.46 | 0.6632268 | 1 |
| nitrogen compound catabolic process | 0.34 | 0.46 | 0.6632268 | 1 |
| myeloid leukocyte activation | 0.34 | 0.46 | 0.6632268 | 1 |
| carboxylic acid metabolic process | 3.85 | 4.41 | 0.6661591 | 1 |
| embryonic morphogenesis | 1.8 | 1.39 | 0.667289 | 1 |
| cell ion homeostasis | 1.8 | 1.39 | 0.667289 | 1 |
| fat cell differentiation | 0.51 | 0.23 | 0.6820303 | 1 |
| determination of symmetry | 0.51 | 0.23 | 0.6820303 | 1 |
| carbohydrate transport | 0.51 | 0.23 | 0.6820303 | 1 |
| cell cycle phase | 2.4 | 1.86 | 0.7038767 | 1 |
| organic acid biosynthetic process | 0.51 | 0.7 | 0.7090337 | 1 |
| response to virus | 0.6 | 0.7 | 0.7343629 | 1 |
| regulation of neurological process | 0.6 | 0.7 | 0.7343629 | 1 |
| regulation of cell organization and biogenesis | 0.77 | 0.46 | 0.7372031 | 1 |
| innate immune response | 0.77 | 0.93 | 0.7568814 | 1 |
| negative regulation of enzyme activity | 0.94 | 0.7 | 0.7705156 | 1 |
| regulation of hydrolase activity | 0.94 | 1.16 | 0.7772335 | 1 |
| tube morphogenesis | 1.03 | 1.16 | 0.7872186 | 1 |
| macromolecule biosynthetic process | 5.39 | 4.87 | 0.800563 | 1 |
| anatomical structure formation | 1.71 | 1.39 | 0.8244722 | 1 |
| homophilic cell adhesion | 1.63 | 1.39 | 0.8244781 | 1 |

| | | | | |
|---|---|---|---|---|
| regulation of cell differentiation | 2.14 | 1.86 | 0.8441696 | 1 |
| electron transport | 2.14 | 1.86 | 0.8441696 | 1 |
| regulation of protein metabolic process | 2.22 | 2.32 | 0.8517048 | 1 |
| cellular carbohydrate metabolic process | 3.08 | 3.25 | 0.8717725 | 1 |
| establishment of cellular localization | 5.56 | 5.8 | 0.9026782 | 1 |
| protein transport | 5.9 | 6.03 | 0.9055007 | 1 |
| peroxisome organization and biogenesis | 0.17 | 0.23 | 1 | 1 |
| pregnancy | 0.34 | 0.23 | 1 | 1 |
| sulfur utilization | 0.09 | 0 | 1 | 1 |
| intercellular junction assembly and maintenance | 0.17 | 0.23 | 1 | 1 |
| humoral immune response | 1.2 | 1.16 | 1 | 1 |
| energy derivation by oxidation of organic compounds | 1.37 | 1.16 | 1 | 1 |
| muscle cell differentiation | 0.68 | 0.7 | 1 | 1 |
| DNA metabolic process | 4.96 | 4.87 | 1 | 1 |
| regulation of helicase activity | 0.09 | 0 | 1 | 1 |
| neurotransmitter transport | 0.68 | 0.46 | 1 | 1 |
| regulation of lipid metabolic process | 0.09 | 0 | 1 | 1 |
| transcription | 18.48 | 18.33 | 1 | 1 |
| pigment biosynthetic process | 0.09 | 0 | 1 | 1 |
| two-component signal transduction system (phosphorela | 0.17 | 0.23 | 1 | 1 |
| porphyrin metabolic process | 0.17 | 0.23 | 1 | 1 |
| detection of hormone stimulus | 0.09 | 0 | 1 | 1 |
| keratinocyte differentiation | 0.51 | 0.46 | 1 | 1 |
| behavioral fear response | 0.17 | 0.23 | 1 | 1 |
| polyol transport | 0.09 | 0 | 1 | 1 |
| maternal behavior | 0.17 | 0.23 | 1 | 1 |
| antigen processing and presentation via MHC class Ib | 0.09 | 0 | 1 | 1 |
| alkene biosynthetic process | 0.17 | 0.23 | 1 | 1 |
| mating behavior | 0.17 | 0 | 1 | 1 |
| mechanoreceptor differentiation | 0.26 | 0.23 | 1 | 1 |
| protein complex assembly | 2.74 | 2.55 | 1 | 1 |
| response to metal ion | 0.09 | 0 | 1 | 1 |
| imprinting | 0.09 | 0 | 1 | 1 |
| autophagic vacuole formation | 0.09 | 0 | 1 | 1 |
| male gamete generation | 1.28 | 1.16 | 1 | 1 |
| regulation of growth rate | 0.09 | 0 | 1 | 1 |

| | | | | |
|---|---|---|---|---|
| menstrual cycle | 0.17 | 0.23 | 1 | 1 |
| vesicle-mediated transport | 4.7 | 4.64 | 1 | 1 |
| membrane fusion | 0.26 | 0.23 | 1 | 1 |
| carbohydrate catabolic process | 0.77 | 0.7 | 1 | 1 |
| positive regulation of developmental process | 0.86 | 0.93 | 1 | 1 |
| protein-RNA complex assembly | 0.68 | 0.7 | 1 | 1 |
| behavioral response to pain | 0.09 | 0 | 1 | 1 |
| lymphocyte homeostasis | 0.17 | 0 | 1 | 1 |
| indole and derivative metabolic process | 0.09 | 0 | 1 | 1 |
| startle response | 0.09 | 0 | 1 | 1 |
| polyol metabolic process | 0.17 | 0.23 | 1 | 1 |
| regulation of pH | 0.26 | 0.23 | 1 | 1 |
| sperm motility | 0.17 | 0.23 | 1 | 1 |
| nucleobase, nucleoside, nucleotide and nucleic acid trar | 0.43 | 0.23 | 1 | 1 |
| sulfation | 0.17 | 0 | 1 | 1 |
| taxis | 1.28 | 1.16 | 1 | 1 |
| sensory perception of pain | 0.26 | 0.23 | 1 | 1 |
| endocrine system development | 0.09 | 0 | 1 | 1 |
| water homeostasis | 0.09 | 0 | 1 | 1 |
| viral infectious cycle | 0.43 | 0.23 | 1 | 1 |
| morphogenesis of an epithelium | 1.2 | 1.16 | 1 | 1 |
| antigen processing and presentation of exogenous antig | 0.09 | 0 | 1 | 1 |
| regulation of response to biotic stimulus | 0.09 | 0 | 1 | 1 |
| positive regulation of response to stimulus | 0.09 | 0 | 1 | 1 |
| positive regulation of gene expression, epigenetic | 0.09 | 0 | 1 | 1 |
| fluid transport | 0.09 | 0 | 1 | 1 |
| detection of tumor cell | 0.09 | 0 | 1 | 1 |
| immune response to tumor cell | 0.09 | 0 | 1 | 1 |
| nutrient import | 0.09 | 0 | 1 | 1 |
| hydrogen transport | 0.43 | 0.23 | 1 | 1 |
| positive regulation of multicellular organismal process | 1.11 | 0.93 | 1 | 1 |
| positive regulation of cytokine production | 0.09 | 0 | 1 | 1 |
| regulation of embryonic development | 0.17 | 0 | 1 | 1 |
| nucleoside salvage | 0.17 | 0 | 1 | 1 |
| vasodilation | 0.09 | 0 | 1 | 1 |
| regulation of cell redox homeostasis | 0.09 | 0 | 1 | 1 |

| | | | | |
|---|---|---|---|---|
| maintenance of cellular localization | 0.09 | 0 | 1 | 1 |
| gas transport | 0.09 | 0 | 1 | 1 |
| response to axon injury | 0.09 | 0 | 1 | 1 |
| methylation | 0.34 | 0.23 | 1 | 1 |
| regeneration | 0.09 | 0 | 1 | 1 |
| antigen processing and presentation of peptide or polysa | 0.09 | 0 | 1 | 1 |
| generation of a signal involved in cell-cell signaling | 1.11 | 1.16 | 1 | 1 |
| pigment cell differentiation | 0.17 | 0 | 1 | 1 |
| cofactor catabolic process | 0.26 | 0.23 | 1 | 1 |
| phenol metabolic process | 0.51 | 0.46 | 1 | 1 |
| myeloid cell homeostasis | 0.09 | 0 | 1 | 1 |
| organic acid transport | 0.51 | 0.46 | 1 | 1 |
| organelle localization | 0.26 | 0.23 | 1 | 1 |
| movement in environment of other organism during symb | 0.17 | 0 | 1 | 1 |
| regulation of organ size | 0.09 | 0 | 1 | 1 |
| ethanol metabolic process | 0.09 | 0 | 1 | 1 |
| vitamin transport | 0.17 | 0 | 1 | 1 |
| regulation of biosynthetic process | 1.37 | 1.39 | 1 | 1 |
| aromatic compound catabolic process | 0.17 | 0.23 | 1 | 1 |
| purine salvage | 0.17 | 0 | 1 | 1 |
| hormone catabolic process | 0.09 | 0 | 1 | 1 |
| negative regulation of gene expression, epigenetic | 0.09 | 0 | 1 | 1 |
| positive regulation of locomotion | 0.17 | 0.23 | 1 | 1 |
| quinone cofactor metabolic process | 0.09 | 0 | 1 | 1 |
| suckling behavior | 0.17 | 0 | 1 | 1 |
| cofactor transport | 0.09 | 0 | 1 | 1 |
| ciliary or flagellar motility | 0.17 | 0 | 1 | 1 |
| ribosome biogenesis and assembly | 0.17 | 0.23 | 1 | 1 |
| cell-matrix adhesion | 0.43 | 0.46 | 1 | 1 |
| amino acid derivative metabolic process | 0.77 | 0.7 | 1 | 1 |
| cell envelope organization and biogenesis | 0.17 | 0 | 1 | 1 |
| nucleotide-sugar metabolic process | 0.09 | 0 | 1 | 1 |
| memory | 0.17 | 0.23 | 1 | 1 |
| protein complex disassembly | 0.09 | 0 | 1 | 1 |
| adaptive immune response | 0.68 | 0.7 | 1 | 1 |
| modification of morphology or physiology of other organi | 0.09 | 0 | 1 | 1 |

| | | | | |
|---|---|---|---|---|
| positive regulation of enzyme activity | 2.14 | 2.09 | 1 | 1 |
| nitric oxide biosynthetic process | 0.17 | 0.23 | 1 | 1 |
| response to X-ray | 0.09 | 0 | 1 | 1 |
| cell glucose homeostasis | 0.09 | 0 | 1 | 1 |

| Gene Ontology : biological process. Level: 6 | Percentage of genes with allele-specific expression | Percentage of genes without allele-specific expression | Unadjusted pvalue | Adjusted pvalueFDR |
|---|---|---|---|---|
| protein modification | 18.93 | 11.87 | 0.001320007 | 1 |
| sensory perception of smell | 1.02 | 3.03 | 0.00867637 | 1 |
| G-protein coupled receptor protein signaling pathway | 6.93 | 10.86 | 0.01683893 | 1 |
| adult walking behavior | 0 | 0.76 | 0.01908822 | 1 |
| phosphorylation | 8.86 | 5.3 | 0.02899869 | 1 |
| embryonic development (sensu Vertebrata) | 1.11 | 0 | 0.04382327 | 1 |
| visual behavior | 0.09 | 0.76 | 0.06110558 | 1 |
| visual perception | 1.66 | 3.28 | 0.06460708 | 1 |
| anterior/posterior pattern formation | 0.92 | 0 | 0.0709744 | 1 |
| DNA strand elongation | 0 | 0.51 | 0.0715566 | 1 |
| negative regulation of bone remodeling | 0 | 0.51 | 0.0715566 | 1 |
| regulation of phospholipase A2 activity | 0 | 0.51 | 0.0715566 | 1 |
| cellular lipid catabolic process | 0.28 | 1.01 | 0.08760815 | 1 |
| cellular polysaccharide metabolic process | 0.28 | 1.01 | 0.08760815 | 1 |
| amine biosynthetic process | 0.65 | 1.52 | 0.122405 | 1 |
| smoothened signaling pathway | 0.18 | 0.76 | 0.12275 | 1 |
| energy reserve metabolic process | 0.18 | 0.76 | 0.12275 | 1 |
| regulation of kinase activity | 2.12 | 3.54 | 0.1336111 | 1 |
| protein oligomerization | 0.46 | 1.26 | 0.1436778 | 1 |
| defense response to bacterium | 0.46 | 1.26 | 0.1436778 | 1 |
| sensory organ development | 0.83 | 1.77 | 0.1531851 | 1 |
| DNA recombination | 0.83 | 1.77 | 0.1531851 | 1 |
| regulation of cell size | 1.94 | 0.76 | 0.1609293 | 1 |
| protein catabolic process | 1.94 | 3.28 | 0.1678934 | 1 |
| modification-dependent macromolecule catabolic proces | 1.39 | 2.53 | 0.1689792 | 1 |
| detection of light stimulus | 0.09 | 0.51 | 0.1764934 | 1 |
| defense response to fungus | 0.09 | 0.51 | 0.1764934 | 1 |
| neuron adhesion | 0.09 | 0.51 | 0.1764934 | 1 |
| vitamin A metabolic process | 0.09 | 0.51 | 0.1764934 | 1 |
| synaptic vesicle transport | 0.28 | 0.76 | 0.1980979 | 1 |
| positive regulation of immune system process | 1.02 | 0.25 | 0.1995551 | 1 |
| exocytosis | 1.11 | 2.02 | 0.2034832 | 1 |
| synaptic transmission | 4.16 | 5.81 | 0.2062397 | 1 |
| kidney development | 0.74 | 1.52 | 0.2214429 | 1 |
| Wnt receptor signaling pathway | 1.39 | 2.27 | 0.2470893 | 1 |

| | | | | |
|---|---|---|---|---|
| positive regulation of cell differentiation | 0.46 | 1.01 | 0.2598048 | 1 |
| Notch signaling pathway | 0.46 | 1.01 | 0.2598048 | 1 |
| cyclic nucleotide metabolic process | 0.46 | 1.01 | 0.2598048 | 1 |
| regulation of cell migration | 0.46 | 1.01 | 0.2598048 | 1 |
| glutamate signaling pathway | 0.46 | 1.01 | 0.2598048 | 1 |
| biopolymer glycosylation | 1.94 | 1.01 | 0.261897 | 1 |
| mRNA metabolic process | 2.03 | 1.01 | 0.2631478 | 1 |
| carbohydrate mediated signaling | 0 | 0.25 | 0.2677485 | 1 |
| locomotor rhythm | 0 | 0.25 | 0.2677485 | 1 |
| positive regulation of bone remodeling | 0 | 0.25 | 0.2677485 | 1 |
| S-adenosylmethionine metabolic process | 0 | 0.25 | 0.2677485 | 1 |
| protein insertion into membrane | 0 | 0.25 | 0.2677485 | 1 |
| baroreceptor feedback regulation of blood pressure | 0 | 0.25 | 0.2677485 | 1 |
| circadian sleep/wake cycle | 0 | 0.25 | 0.2677485 | 1 |
| negative regulation of carbohydrate metabolic process | 0 | 0.25 | 0.2677485 | 1 |
| regulation of nitric oxide biosynthetic process | 0 | 0.25 | 0.2677485 | 1 |
| peptide modification | 0 | 0.25 | 0.2677485 | 1 |
| positive regulation of catabolic process | 0 | 0.25 | 0.2677485 | 1 |
| polysaccharide catabolic process | 0 | 0.25 | 0.2677485 | 1 |
| response to cocaine | 0 | 0.25 | 0.2677485 | 1 |
| carboxylic acid catabolic process | 0 | 0.25 | 0.2677485 | 1 |
| regulation of blood pressure by hormones | 0 | 0.25 | 0.2677485 | 1 |
| superoxide release | 0 | 0.25 | 0.2677485 | 1 |
| behavioral response to ethanol | 0 | 0.25 | 0.2677485 | 1 |
| amino acid derivative catabolic process | 0 | 0.25 | 0.2677485 | 1 |
| sensory perception of taste | 0 | 0.25 | 0.2677485 | 1 |
| regulation of cellular catabolic process | 0 | 0.25 | 0.2677485 | 1 |
| phospholipid scrambling | 0 | 0.25 | 0.2677485 | 1 |
| response to reactive oxygen species | 0 | 0.25 | 0.2677485 | 1 |
| response to morphine | 0 | 0.25 | 0.2677485 | 1 |
| muscle cell development | 0 | 0.25 | 0.2677485 | 1 |
| centrosome organization and biogenesis | 0 | 0.25 | 0.2677485 | 1 |
| antibiotic biosynthetic process | 0 | 0.25 | 0.2677485 | 1 |
| detection of bacterium | 0 | 0.25 | 0.2677485 | 1 |
| negative regulation of catabolic process | 0 | 0.25 | 0.2677485 | 1 |
| DNA geometric change | 0 | 0.25 | 0.2677485 | 1 |

| | | | | |
|---|---|---|---|---|
| glutathione metabolic process | 0 | 0.25 | 0.2677485 | 1 |
| DNA ligation | 0 | 0.25 | 0.2677485 | 1 |
| regulation of vasoconstriction | 0 | 0.25 | 0.2677485 | 1 |
| nitric oxide mediated signal transduction | 0 | 0.25 | 0.2677485 | 1 |
| RNA processing | 2.95 | 1.77 | 0.2713158 | 1 |
| substrate-bound cell migration | 0.18 | 0.51 | 0.2918811 | 1 |
| cell fate determination | 0.18 | 0.51 | 0.2918811 | 1 |
| cell-cell signaling during cell fate commitment | 0.18 | 0.51 | 0.2918811 | 1 |
| glycerolipid metabolic process | 0.18 | 0.51 | 0.2918811 | 1 |
| mesenchymal cell development | 0.18 | 0.51 | 0.2918811 | 1 |
| biopolymer biosynthetic process | 0.18 | 0.51 | 0.2918811 | 1 |
| peptidoglycan metabolic process | 0.18 | 0.51 | 0.2918811 | 1 |
| neutral lipid metabolic process | 0.18 | 0.51 | 0.2918811 | 1 |
| female sex differentiation | 0.18 | 0.51 | 0.2918811 | 1 |
| monosaccharide metabolic process | 1.57 | 0.76 | 0.3124353 | 1 |
| skeletal development | 2.95 | 4.04 | 0.3202762 | 1 |
| central nervous system development | 3.05 | 4.04 | 0.3297796 | 1 |
| oxidoreduction coenzyme metabolic process | 0.55 | 0 | 0.351682 | 1 |
| membrane lipid metabolic process | 1.39 | 2.02 | 0.3538307 | 1 |
| anion transport | 1.39 | 2.02 | 0.3538307 | 1 |
| positive regulation of cellular metabolic process | 4.25 | 3.03 | 0.3638177 | 1 |
| regulation of immune response | 1.11 | 0.51 | 0.3761687 | 1 |
| blood coagulation | 1.11 | 0.51 | 0.3761687 | 1 |
| main pathways of carbohydrate metabolic process | 1.2 | 0.51 | 0.379194 | 1 |
| coenzyme biosynthetic process | 1.2 | 0.51 | 0.379194 | 1 |
| negative regulation of signal transduction | 1.2 | 0.51 | 0.379194 | 1 |
| aminoglycan metabolic process | 0.37 | 0.76 | 0.3932599 | 1 |
| cell death | 7.2 | 5.81 | 0.4150825 | 1 |
| glycoprotein metabolic process | 2.31 | 1.52 | 0.4168532 | 1 |
| endocytosis | 2.31 | 1.52 | 0.4168532 | 1 |
| tissue development | 3.14 | 4.04 | 0.4171678 | 1 |
| sensory perception of sound | 1.39 | 0.76 | 0.4290147 | 1 |
| monocarboxylic acid metabolic process | 2.12 | 2.78 | 0.438683 | 1 |
| negative regulation of transferase activity | 0.46 | 0.76 | 0.4480905 | 1 |
| regulation of transmission of nerve impulse | 0.46 | 0.76 | 0.4480905 | 1 |
| establishment and/or maintenance of cell polarity | 0.46 | 0.76 | 0.4480905 | 1 |

| | | | | |
|---|---|---|---|---|
| negative regulation of cellular metabolic process | 4.25 | 3.28 | 0.4556657 | 1 |
| intracellular receptor-mediated signaling pathway | 0.74 | 0.25 | 0.4586838 | 1 |
| regulation of ion transport | 0.09 | 0.25 | 0.4639404 | 1 |
| thermoception | 0.09 | 0.25 | 0.4639404 | 1 |
| pteridine and derivative biosynthetic process | 0.09 | 0.25 | 0.4639404 | 1 |
| peptide secretion | 0.09 | 0.25 | 0.4639404 | 1 |
| negative regulation of cell motility | 0.09 | 0.25 | 0.4639404 | 1 |
| regulation of smooth muscle contraction | 0.09 | 0.25 | 0.4639404 | 1 |
| positive regulation of heart contraction | 0.09 | 0.25 | 0.4639404 | 1 |
| molybdopterin cofactor metabolic process | 0.09 | 0.25 | 0.4639404 | 1 |
| positive regulation of carbohydrate metabolic process | 0.09 | 0.25 | 0.4639404 | 1 |
| ovulation | 0.09 | 0.25 | 0.4639404 | 1 |
| regulation of nitric-oxide synthase activity | 0.09 | 0.25 | 0.4639404 | 1 |
| natural killer cell activation | 0.09 | 0.25 | 0.4639404 | 1 |
| porphyrin catabolic process | 0.09 | 0.25 | 0.4639404 | 1 |
| leukocyte activation during immune response | 0.09 | 0.25 | 0.4639404 | 1 |
| arachidonic acid secretion | 0.09 | 0.25 | 0.4639404 | 1 |
| plasma membrane repair | 0.09 | 0.25 | 0.4639404 | 1 |
| oligopeptide transport | 0.09 | 0.25 | 0.4639404 | 1 |
| intestinal absorption | 0.09 | 0.25 | 0.4639404 | 1 |
| morphogenesis of an epithelial sheet | 0.09 | 0.25 | 0.4639404 | 1 |
| lipoprotein metabolic process | 0.55 | 1.01 | 0.4718883 | 1 |
| acute inflammatory response | 0.55 | 1.01 | 0.4718883 | 1 |
| positive regulation of transferase activity | 1.48 | 2.02 | 0.4873266 | 1 |
| branching morphogenesis of a tube | 0.65 | 1.01 | 0.4982371 | 1 |
| positive regulation of biosynthetic process | 0.65 | 1.01 | 0.4982371 | 1 |
| regulation of cell morphogenesis | 0.65 | 1.01 | 0.4982371 | 1 |
| neurogenesis | 5.26 | 4.29 | 0.5025364 | 1 |
| actin filament-based process | 3.42 | 2.53 | 0.5030137 | 1 |
| cell projection morphogenesis | 3.42 | 2.53 | 0.5030137 | 1 |
| limb morphogenesis | 0.92 | 0.51 | 0.5323865 | 1 |
| embryonic appendage morphogenesis | 0.92 | 0.51 | 0.5323865 | 1 |
| microtubule-based process | 2.22 | 1.52 | 0.5325448 | 1 |
| cytokine metabolic process | 0.92 | 1.26 | 0.56311 | 1 |
| regulation of fat cell differentiation | 0.28 | 0 | 0.5688295 | 1 |
| response to UV | 0.28 | 0 | 0.5688295 | 1 |

| | | | | |
|---|---|---|---|---|
| receptor metabolic process | 0.28 | 0 | 0.5688295 | 1 |
| cardiac inotropy | 0.28 | 0 | 0.5688295 | 1 |
| response to nutrient | 0.28 | 0 | 0.5688295 | 1 |
| cytokine secretion | 0.28 | 0 | 0.5688295 | 1 |
| cortical cytoskeleton organization and biogenesis | 0.28 | 0 | 0.5688295 | 1 |
| acetyl-CoA metabolic process | 0.28 | 0 | 0.5688295 | 1 |
| leukocyte adhesion | 0.28 | 0 | 0.5688295 | 1 |
| RNA modification | 0.28 | 0 | 0.5688295 | 1 |
| positive regulation of cell organization and biogenesis | 0.28 | 0 | 0.5688295 | 1 |
| nucleobase biosynthetic process | 0.28 | 0 | 0.5688295 | 1 |
| nucleoside monophosphate metabolic process | 0.28 | 0 | 0.5688295 | 1 |
| regulation of inflammatory response | 0.28 | 0 | 0.5688295 | 1 |
| germ cell migration | 0.28 | 0 | 0.5688295 | 1 |
| negative regulation of cyclase activity | 0.37 | 0 | 0.5789563 | 1 |
| negative regulation of lyase activity | 0.37 | 0 | 0.5789563 | 1 |
| immune response-regulating cell surface receptor signal | 0.37 | 0 | 0.5789563 | 1 |
| regulation of protein secretion | 0.37 | 0 | 0.5789563 | 1 |
| positive regulation of secretion | 0.37 | 0 | 0.5789563 | 1 |
| water-soluble vitamin biosynthetic process | 0.37 | 0 | 0.5789563 | 1 |
| cellular respiration | 0.37 | 0 | 0.5789563 | 1 |
| protein import | 1.11 | 1.52 | 0.5923102 | 1 |
| vasculature development | 2.86 | 2.27 | 0.592668 | 1 |
| heart development | 1.2 | 1.52 | 0.6082916 | 1 |
| cellular morphogenesis during differentiation | 3.23 | 2.53 | 0.6083547 | 1 |
| amino acid derivative biosynthetic process | 0.28 | 0.51 | 0.614638 | 1 |
| proteoglycan metabolic process | 0.28 | 0.51 | 0.614638 | 1 |
| negative regulation of cell organization and biogenesis | 0.28 | 0.51 | 0.614638 | 1 |
| regulation of epithelial cell proliferation | 0.28 | 0.51 | 0.614638 | 1 |
| lysosome organization and biogenesis | 0.28 | 0.51 | 0.614638 | 1 |
| protein depolymerization | 0.28 | 0.51 | 0.614638 | 1 |
| cytokine and chemokine mediated signaling pathway | 0.28 | 0.51 | 0.614638 | 1 |
| protein folding | 1.48 | 1.01 | 0.6165122 | 1 |
| DNA replication | 1.48 | 1.77 | 0.6420987 | 1 |
| small GTPase mediated signal transduction | 4.25 | 3.54 | 0.6554219 | 1 |
| regulation of nucleobase, nucleoside, nucleotide and nu | 19.3 | 20.45 | 0.6572536 | 1 |
| regulation of action potential | 0.37 | 0.51 | 0.661488 | 1 |

| | | | | |
|---|---|---|---|---|
| amine catabolic process | 0.37 | 0.51 | 0.661488 | 1 |
| gastrulation | 0.37 | 0.51 | 0.661488 | 1 |
| protein kinase cascade | 4.52 | 3.79 | 0.6650541 | 1 |
| M phase | 1.94 | 1.52 | 0.6676904 | 1 |
| lipid biosynthetic process | 1.85 | 2.27 | 0.6717916 | 1 |
| segmentation | 0.55 | 0.25 | 0.6822859 | 1 |
| determination of bilateral symmetry | 0.55 | 0.25 | 0.6822859 | 1 |
| positive regulation of transport | 0.55 | 0.25 | 0.6822859 | 1 |
| DNA packaging | 2.22 | 1.77 | 0.6859411 | 1 |
| germ cell development | 0.65 | 0.25 | 0.6897683 | 1 |
| group transfer coenzyme metabolic process | 0.65 | 0.25 | 0.6897683 | 1 |
| epithelial cell differentiation | 0.65 | 0.25 | 0.6897683 | 1 |
| cation transport | 5.54 | 6.06 | 0.7042588 | 1 |
| carboxylic acid biosynthetic process | 0.55 | 0.76 | 0.7079658 | 1 |
| cellular protein complex assembly | 0.55 | 0.76 | 0.7079658 | 1 |
| development of primary sexual characteristics | 0.55 | 0.76 | 0.7079658 | 1 |
| regulation of progression through cell cycle | 5.72 | 6.31 | 0.7081859 | 1 |
| intracellular transport | 5.82 | 6.31 | 0.7107259 | 1 |
| muscle development | 2.49 | 2.78 | 0.7144068 | 1 |
| positive regulation of cell proliferation | 2.49 | 2.78 | 0.7144068 | 1 |
| chromosome organization and biogenesis (sensu Eukar | 2.77 | 2.27 | 0.7152497 | 1 |
| positive regulation of hydrolase activity | 0.65 | 0.76 | 0.7327987 | 1 |
| negative regulation of protein metabolic process | 0.65 | 0.76 | 0.7327987 | 1 |
| ribonucleotide metabolic process | 0.83 | 0.51 | 0.7372247 | 1 |
| biogenic amine metabolic process | 0.83 | 0.51 | 0.7372247 | 1 |
| leukocyte mediated immunity | 0.74 | 1.01 | 0.7434302 | 1 |
| cell maturation | 0.83 | 1.01 | 0.7558127 | 1 |
| antimicrobial humoral response | 0.83 | 1.01 | 0.7558127 | 1 |
| regulation of lymphocyte activation | 0.83 | 1.01 | 0.7558127 | 1 |
| negative regulation of cell differentiation | 0.83 | 1.01 | 0.7558127 | 1 |
| carbohydrate biosynthetic process | 1.02 | 1.26 | 0.7765899 | 1 |
| regulated secretory pathway | 1.02 | 1.26 | 0.7765899 | 1 |
| steroid metabolic process | 1.29 | 1.01 | 0.7935596 | 1 |
| T cell activation | 1.57 | 1.26 | 0.8107554 | 1 |
| dephosphorylation | 1.75 | 2.02 | 0.8263799 | 1 |
| amino acid metabolic process | 1.85 | 2.02 | 0.8305251 | 1 |

| | | | | |
|---|---|---|---|---|
| organ morphogenesis | 4.34 | 4.55 | 0.8862671 | 1 |
| RNA biosynthetic process | 18.47 | 18.69 | 0.9397939 | 1 |
| synaptogenesis | 0.28 | 0.25 | 1 | 1 |
| B cell activation | 1.11 | 1.01 | 1 | 1 |
| mast cell activation | 0.18 | 0.25 | 1 | 1 |
| positive regulation of cyclase activity | 0.09 | 0 | 1 | 1 |
| benzene and derivative metabolic process | 0.09 | 0 | 1 | 1 |
| regulation of pigment cell differentiation | 0.09 | 0 | 1 | 1 |
| hemoglobin metabolic process | 0.18 | 0 | 1 | 1 |
| negative regulation of helicase activity | 0.09 | 0 | 1 | 1 |
| deoxyribonucleoside metabolic process | 0.09 | 0 | 1 | 1 |
| uroporphyrinogen III metabolic process | 0.09 | 0 | 1 | 1 |
| ameboidal cell migration | 0.18 | 0 | 1 | 1 |
| modification of host morphology or physiology | 0.09 | 0 | 1 | 1 |
| immune system development | 2.77 | 2.78 | 1 | 1 |
| positive regulation of protein metabolic process | 1.02 | 1.01 | 1 | 1 |
| oogenesis | 0.18 | 0.25 | 1 | 1 |
| amino sugar metabolic process | 0.18 | 0 | 1 | 1 |
| regulation of smooth muscle cell proliferation | 0.09 | 0 | 1 | 1 |
| negative regulation of heart contraction | 0.09 | 0 | 1 | 1 |
| morphogenesis of a polarized epithelium | 0.09 | 0 | 1 | 1 |
| spermatogenesis | 1.39 | 1.26 | 1 | 1 |
| stress-activated protein kinase signaling pathway | 0.55 | 0.51 | 1 | 1 |
| vitamin D metabolic process | 0.09 | 0 | 1 | 1 |
| purine nucleoside metabolic process | 0.18 | 0 | 1 | 1 |
| negative regulation of neurological process | 0.09 | 0 | 1 | 1 |
| DNA catabolic process | 0.18 | 0 | 1 | 1 |
| regulation of muscle cell differentiation | 0.09 | 0 | 1 | 1 |
| embryonic axis specification | 0.09 | 0 | 1 | 1 |
| DNA damage response, signal transduction by p53 class | 0.18 | 0 | 1 | 1 |
| vesicle fusion | 0.18 | 0.25 | 1 | 1 |
| cellular carbohydrate catabolic process | 0.83 | 0.76 | 1 | 1 |
| formation of immunological synapse | 0.09 | 0 | 1 | 1 |
| cell fate specification | 0.18 | 0 | 1 | 1 |
| DNA repair | 1.94 | 1.77 | 1 | 1 |
| pyrimidine base metabolic process | 0.18 | 0 | 1 | 1 |

| | | | | |
|---|---|---|---|---|
| pyrimidine nucleoside metabolic process | 0.09 | 0 | 1 | 1 |
| translation | 2.49 | 2.53 | 1 | 1 |
| movement in host environment | 0.18 | 0 | 1 | 1 |
| melanocyte differentiation | 0.18 | 0 | 1 | 1 |
| positive regulation of lipid metabolic process | 0.09 | 0 | 1 | 1 |
| nucleotide-sugar biosynthetic process | 0.09 | 0 | 1 | 1 |
| nucleic acid transport | 0.37 | 0.25 | 1 | 1 |
| negative regulation of secretion | 0.18 | 0 | 1 | 1 |
| lipid raft organization and biogenesis | 0.18 | 0 | 1 | 1 |
| dorsal/ventral pattern formation | 0.28 | 0.25 | 1 | 1 |
| mitochondrial membrane organization and biogenesis | 0.09 | 0 | 1 | 1 |
| phosphoenolpyruvate-dependent sugar phosphotransfer | 0.09 | 0 | 1 | 1 |
| MHC class II biosynthetic process | 0.18 | 0 | 1 | 1 |
| proximal/distal pattern formation | 0.09 | 0 | 1 | 1 |
| positive regulation of lyase activity | 0.09 | 0 | 1 | 1 |
| negative regulation of defense response | 0.18 | 0 | 1 | 1 |
| nucleotide-sugar transport | 0.09 | 0 | 1 | 1 |
| regulation of cellular biosynthetic process | 1.39 | 1.26 | 1 | 1 |
| viral genome replication | 0.28 | 0.25 | 1 | 1 |
| positive regulation of cell motility | 0.18 | 0.25 | 1 | 1 |
| adaptive immune response (sensu Gnathostomata) | 0.74 | 0.76 | 1 | 1 |
| disruption of cells of other organism during symbiotic inte | 0.09 | 0 | 1 | 1 |
| detection of fungus | 0.09 | 0 | 1 | 1 |
| response to mercury ion | 0.09 | 0 | 1 | 1 |
| catecholamine transport | 0.09 | 0 | 1 | 1 |
| long-term memory | 0.09 | 0 | 1 | 1 |
| nucleus localization | 0.09 | 0 | 1 | 1 |
| ER-nuclear signaling pathway | 0.28 | 0.25 | 1 | 1 |
| glycerol metabolic process | 0.18 | 0.25 | 1 | 1 |
| rRNA metabolic process | 0.28 | 0.25 | 1 | 1 |
| mitochondrial genome maintenance | 0.09 | 0 | 1 | 1 |
| endodermal cell fate commitment | 0.09 | 0 | 1 | 1 |
| morphogenesis of embryonic epithelium | 0.55 | 0.51 | 1 | 1 |
| nucleoside catabolic process | 0.09 | 0 | 1 | 1 |
| fatty acid transport | 0.09 | 0 | 1 | 1 |
| dicarboxylic acid metabolic process | 0.18 | 0 | 1 | 1 |

| | | | | |
|---|---|---|---|---|
| respiratory tube development | 0.55 | 0.51 | 1 | 1 |
| hormone secretion | 0.28 | 0.25 | 1 | 1 |
| male pronucleus formation | 0.09 | 0 | 1 | 1 |
| production of molecular mediator of immune response | 0.18 | 0.25 | 1 | 1 |
| lipid modification | 0.28 | 0.25 | 1 | 1 |
| nucleoside triphosphate metabolic process | 0.46 | 0.51 | 1 | 1 |
| neutrophil homeostasis | 0.09 | 0 | 1 | 1 |
| T cell homeostasis | 0.18 | 0 | 1 | 1 |
| defense response to virus | 0.18 | 0 | 1 | 1 |
| biopolymer methylation | 0.18 | 0 | 1 | 1 |
| purine base metabolic process | 0.09 | 0 | 1 | 1 |
| second-messenger-mediated signaling | 2.95 | 2.78 | 1 | 1 |
| posttranscriptional gene silencing | 0.09 | 0 | 1 | 1 |
| dopamine transport | 0.09 | 0 | 1 | 1 |
| establishment of organelle localization | 0.28 | 0.25 | 1 | 1 |
| maintenance of DNA repeat elements | 0.09 | 0 | 1 | 1 |
| cytoskeletal anchoring | 0.09 | 0 | 1 | 1 |
| ribonucleoside metabolic process | 0.18 | 0 | 1 | 1 |
| endothelial cell migration | 0.09 | 0 | 1 | 1 |
| thiamin and derivative metabolic process | 0.09 | 0 | 1 | 1 |
| RNA catabolic process | 0.18 | 0 | 1 | 1 |
| entry into other organism during symbiotic interaction | 0.18 | 0 | 1 | 1 |
| nucleobase catabolic process | 0.09 | 0 | 1 | 1 |
| protein polymerization | 0.55 | 0.51 | 1 | 1 |
| negative regulation of biosynthetic process | 0.46 | 0.25 | 1 | 1 |
| water transport | 0.09 | 0 | 1 | 1 |
| regulation of fibroblast proliferation | 0.18 | 0.25 | 1 | 1 |
| regulation of vasodilation | 0.09 | 0 | 1 | 1 |
| peripheral nervous system development | 0.28 | 0.25 | 1 | 1 |
| anion homeostasis | 0.09 | 0 | 1 | 1 |
| embryonic organ development | 0.28 | 0.25 | 1 | 1 |
| choline transport | 0.09 | 0 | 1 | 1 |
| lymphocyte proliferation | 0.83 | 0.76 | 1 | 1 |
| regulation of GTPase activity | 0.46 | 0.51 | 1 | 1 |
| regulation of response to tumor cell | 0.09 | 0 | 1 | 1 |
| negative regulation of transport | 0.28 | 0.25 | 1 | 1 |

| | | | | |
|---|---|---|---|---|
| chemotaxis | 1.39 | 1.26 | 1 | 1 |
| tRNA metabolic process | 0.46 | 0.25 | 1 | 1 |
| regulation of sensory perception | 0.09 | 0 | 1 | 1 |
| regulation of antiviral response | 0.09 | 0 | 1 | 1 |
| neural crest cell differentiation | 0.18 | 0 | 1 | 1 |
| hormone-mediated signaling | 0.09 | 0 | 1 | 1 |
| associative learning | 0.18 | 0.25 | 1 | 1 |
| isoprenoid metabolic process | 0.18 | 0.25 | 1 | 1 |
| neural tube development | 0.37 | 0.25 | 1 | 1 |
| regulation of protein stability | 0.18 | 0 | 1 | 1 |
| nuclear body organization and biogenesis | 0.09 | 0 | 1 | 1 |
| positive regulation of defense response | 0.09 | 0 | 1 | 1 |
| regulation of phosphorus metabolic process | 0.65 | 0.51 | 1 | 1 |
| protein maturation | 0.09 | 0 | 1 | 1 |
| initiation of viral infection | 0.18 | 0 | 1 | 1 |
| intercellular junction assembly | 0.18 | 0.25 | 1 | 1 |
| positive regulation of signal transduction | 1.39 | 1.26 | 1 | 1 |
| monoamine transport | 0.09 | 0 | 1 | 1 |
| chromosome condensation | 0.18 | 0.25 | 1 | 1 |
| pH reduction | 0.09 | 0 | 1 | 1 |
| gland development | 0.28 | 0.25 | 1 | 1 |
| male sex differentiation | 0.28 | 0.25 | 1 | 1 |
| indole derivative metabolic process | 0.09 | 0 | 1 | 1 |
| oxygen transport | 0.09 | 0 | 1 | 1 |
| sterol transport | 0.18 | 0 | 1 | 1 |
| pancreas development | 0.09 | 0 | 1 | 1 |
| negative regulation of cell proliferation | 2.86 | 2.78 | 1 | 1 |
| enzyme linked receptor protein signaling pathway | 3.69 | 3.79 | 1 | 1 |
| carboxylic acid transport | 0.55 | 0.51 | 1 | 1 |
| nucleoside diphosphate metabolic process | 0.09 | 0 | 1 | 1 |
| brown fat cell differentiation | 0.09 | 0 | 1 | 1 |
| heme metabolic process | 0.09 | 0 | 1 | 1 |
| modification of structure of other organism during symbi | 0.09 | 0 | 1 | 1 |
| smooth muscle cell differentiation | 0.18 | 0 | 1 | 1 |
| vitamin B6 metabolic process | 0.09 | 0 | 1 | 1 |
| cation homeostasis | 1.75 | 1.52 | 1 | 1 |

| | | | | |
|---|---|---|---|---|
| cobalamin transport | 0.09 | 0 | 1 | 1 |
| purine nucleotide metabolic process | 0.65 | 0.51 | 1 | 1 |
| macrophage activation | 0.28 | 0.25 | 1 | 1 |
| intermediate filament-based process | 0.09 | 0 | 1 | 1 |
| gut development | 0.28 | 0.25 | 1 | 1 |
| cellular protein complex disassembly | 0.09 | 0 | 1 | 1 |
| nucleotide catabolic process | 0.09 | 0 | 1 | 1 |
| negative regulation of organ size | 0.09 | 0 | 1 | 1 |
| sulfate assimilation | 0.09 | 0 | 1 | 1 |
| negative regulation of cell adhesion | 0.18 | 0 | 1 | 1 |
| lipid raft localization | 0.09 | 0 | 1 | 1 |
| aminophospholipid transport | 0.18 | 0 | 1 | 1 |
| leukocyte migration | 0.65 | 0.51 | 1 | 1 |
| embryo implantation | 0.09 | 0 | 1 | 1 |
| regulation of protein complex assembly | 0.09 | 0 | 1 | 1 |
| proteolysis | 6.37 | 6.31 | 1 | 1 |
| integrin-mediated signaling pathway | 0.55 | 0.51 | 1 | 1 |
| nucleotide biosynthetic process | 1.48 | 1.26 | 1 | 1 |
| nucleobase transport | 0.09 | 0 | 1 | 1 |
| porphyrin biosynthetic process | 0.09 | 0 | 1 | 1 |
| response to starvation | 0.09 | 0 | 1 | 1 |
| regulation of endothelial cell proliferation | 0.18 | 0.25 | 1 | 1 |
| regulation of immune effector process | 0.09 | 0 | 1 | 1 |
| regulation of protein transport | 0.18 | 0 | 1 | 1 |
| interphase | 0.74 | 0.51 | 1 | 1 |
| pyrimidine nucleotide metabolic process | 0.28 | 0.25 | 1 | 1 |
| vesicle localization | 0.18 | 0.25 | 1 | 1 |
| coenzyme catabolic process | 0.18 | 0 | 1 | 1 |
| vesicle docking | 0.46 | 0.25 | 1 | 1 |
| B cell homeostasis | 0.09 | 0 | 1 | 1 |
| menstrual cycle process | 0.18 | 0.25 | 1 | 1 |
| genitalia development | 0.18 | 0.25 | 1 | 1 |
| antigen processing and presentation of lipid antigen via I | 0.09 | 0 | 1 | 1 |
| focal adhesion formation | 0.18 | 0 | 1 | 1 |
| positive regulation of response to biotic stimulus | 0.09 | 0 | 1 | 1 |
| positive regulation of cell adhesion | 0.09 | 0 | 1 | 1 |

| | | | | |
|---|---|---|---|---|
| maintenance of cellular protein localization | 0.09 | 0 | 1 | 1 |
| ethanol oxidation | 0.09 | 0 | 1 | 1 |
| RNA-mediated gene silencing | 0.09 | 0 | 1 | 1 |
| monosaccharide transport | 0.28 | 0.25 | 1 | 1 |

| Gene Ontology : biological process. Level: 7 | Percentage of genes with allele-specific expression | Percentage of genes without allele-specific expression | Unadjusted pvalue | Adjusted pvalueFDR |
|---|---|---|---|---|
| protein homooligomerization | 0.11 | 1.55 | 0.005359601 | 1 |
| biomineral formation | 0.76 | 2.8 | 0.009100373 | 1 |
| double-strand break repair | 0.11 | 1.24 | 0.01759404 | 1 |
| visual learning | 0.11 | 0.93 | 0.05560903 | 1 |
| cellular protein catabolic process | 1.95 | 4.04 | 0.05866208 | 1 |
| heparan sulfate proteoglycan metabolic process | 0 | 0.62 | 0.06684507 | 1 |
| adult heart development | 0 | 0.62 | 0.06684507 | 1 |
| epithelial to mesenchymal transition | 0 | 0.62 | 0.06684507 | 1 |
| calcium ion-dependent exocytosis | 0 | 0.62 | 0.06684507 | 1 |
| recombinational repair | 0 | 0.62 | 0.06684507 | 1 |
| positive regulation of phospholipase A2 activity | 0 | 0.62 | 0.06684507 | 1 |
| protein amino acid phosphorylation | 9.87 | 6.52 | 0.07142957 | 1 |
| regulation of protein kinase activity | 2.28 | 4.35 | 0.07548919 | 1 |
| intracellular protein transport | 3.69 | 6.21 | 0.07890425 | 1 |
| membrane lipid catabolic process | 0.22 | 0.93 | 0.1126315 | 1 |
| cyclic nucleotide biosynthetic process | 0.22 | 0.93 | 0.1126315 | 1 |
| acute-phase response | 0.22 | 0.93 | 0.1126315 | 1 |
| inorganic anion transport | 1.19 | 2.48 | 0.1153819 | 1 |
| DNA-dependent DNA replication | 0.65 | 1.55 | 0.1648446 | 1 |
| muscle fiber development | 0.65 | 1.55 | 0.1648446 | 1 |
| positive regulation of epithelial cell proliferation | 0.11 | 0.62 | 0.1660901 | 1 |
| polysaccharide biosynthetic process | 0.11 | 0.62 | 0.1660901 | 1 |
| detection of visible light | 0.11 | 0.62 | 0.1660901 | 1 |
| detection of light stimulus during sensory perception | 0.11 | 0.62 | 0.1660901 | 1 |
| endoderm development | 0.11 | 0.62 | 0.1660901 | 1 |
| fatty acid metabolic process | 1.74 | 3.11 | 0.1721741 | 1 |
| positive regulation of nucleobase, nucleoside, nucleotide | 4.34 | 2.48 | 0.17787 | 1 |
| cGMP metabolic process | 0.33 | 0.93 | 0.1832001 | 1 |
| glycolipid metabolic process | 0.33 | 0.93 | 0.1832001 | 1 |
| embryonic development (sensu Mammalia) | 0.76 | 0 | 0.2005548 | 1 |
| amino acid biosynthetic process | 0.43 | 1.24 | 0.2160468 | 1 |
| ear development | 0.43 | 1.24 | 0.2160468 | 1 |
| cell growth | 2.17 | 0.93 | 0.22816 | 1 |
| mRNA processing | 2.17 | 0.93 | 0.22816 | 1 |
| peptidoglycan catabolic process | 0 | 0.31 | 0.2588424 | 1 |

| | | | | |
|---|---|---|---|---|
| apical junction assembly | 0 | 0.31 | 0.2588424 | 1 |
| germinal center formation | 0 | 0.31 | 0.2588424 | 1 |
| aspartate family amino acid metabolic process | 0 | 0.31 | 0.2588424 | 1 |
| negative regulation of ion transport | 0 | 0.31 | 0.2588424 | 1 |
| photoreceptor cell maintenance | 0 | 0.31 | 0.2588424 | 1 |
| behavioral response to cocaine | 0 | 0.31 | 0.2588424 | 1 |
| lipid phosphorylation | 0 | 0.31 | 0.2588424 | 1 |
| baroreceptor response to lowering of blood pressure | 0 | 0.31 | 0.2588424 | 1 |
| bile acid metabolic process | 0 | 0.31 | 0.2588424 | 1 |
| peroxisomal transport | 0 | 0.31 | 0.2588424 | 1 |
| negative regulation of cellular catabolic process | 0 | 0.31 | 0.2588424 | 1 |
| biogenic amine catabolic process | 0 | 0.31 | 0.2588424 | 1 |
| cellular extravasation | 0 | 0.31 | 0.2588424 | 1 |
| actin filament severing | 0 | 0.31 | 0.2588424 | 1 |
| positive regulation of vasoconstriction | 0 | 0.31 | 0.2588424 | 1 |
| positive regulation of cellular catabolic process | 0 | 0.31 | 0.2588424 | 1 |
| defense response to Gram-positive bacterium | 0 | 0.31 | 0.2588424 | 1 |
| receptor guanylyl cyclase signaling pathway | 0 | 0.31 | 0.2588424 | 1 |
| prostate gland development | 0 | 0.31 | 0.2588424 | 1 |
| cellular polysaccharide catabolic process | 0 | 0.31 | 0.2588424 | 1 |
| regulation of Notch signaling pathway | 0 | 0.31 | 0.2588424 | 1 |
| peptide amidation | 0 | 0.31 | 0.2588424 | 1 |
| salivary gland development | 0 | 0.31 | 0.2588424 | 1 |
| centrosome cycle | 0 | 0.31 | 0.2588424 | 1 |
| antibacterial humoral response | 0 | 0.31 | 0.2588424 | 1 |
| leading edge cell differentiation | 0 | 0.31 | 0.2588424 | 1 |
| metabotropic glutamate receptor signaling pathway | 0 | 0.31 | 0.2588424 | 1 |
| DNA duplex unwinding | 0 | 0.31 | 0.2588424 | 1 |
| antifungal humoral response | 0 | 0.31 | 0.2588424 | 1 |
| mitotic recombination | 0 | 0.31 | 0.2588424 | 1 |
| bilirubin conjugation | 0 | 0.31 | 0.2588424 | 1 |
| non-recombinational repair | 0 | 0.31 | 0.2588424 | 1 |
| positive regulation of nitric oxide biosynthetic process | 0 | 0.31 | 0.2588424 | 1 |
| response to hydrogen peroxide | 0 | 0.31 | 0.2588424 | 1 |
| norepinephrine-epinephrine regulation of blood pressure | 0 | 0.31 | 0.2588424 | 1 |
| ER overload response | 0 | 0.31 | 0.2588424 | 1 |

| | | | | |
|---|---|---|---|---|
| extracellular polysaccharide metabolic process | 0 | 0.31 | 0.2588424 | 1 |
| regulation of urothelial cell proliferation | 0 | 0.31 | 0.2588424 | 1 |
| regulation of vesicle fusion | 0 | 0.31 | 0.2588424 | 1 |
| glycoprotein biosynthetic process | 2.39 | 1.24 | 0.2636428 | 1 |
| dicarboxylic acid transport | 0.22 | 0.62 | 0.2765711 | 1 |
| transmembrane receptor protein tyrosine phosphatase s | 0.22 | 0.62 | 0.2765711 | 1 |
| glucan metabolic process | 0.22 | 0.62 | 0.2765711 | 1 |
| development of primary female sexual characteristics | 0.22 | 0.62 | 0.2765711 | 1 |
| induction | 0.22 | 0.62 | 0.2765711 | 1 |
| glycoprotein catabolic process | 0.22 | 0.62 | 0.2765711 | 1 |
| acylglycerol metabolic process | 0.22 | 0.62 | 0.2765711 | 1 |
| frizzled signaling pathway | 0.22 | 0.62 | 0.2765711 | 1 |
| photoreceptor cell development | 0.22 | 0.62 | 0.2765711 | 1 |
| glutamine family amino acid metabolic process | 0.22 | 0.62 | 0.2765711 | 1 |
| proteoglycan biosynthetic process | 0.22 | 0.62 | 0.2765711 | 1 |
| aminoglycan biosynthetic process | 0.22 | 0.62 | 0.2765711 | 1 |
| negative regulation of progression through cell cycle | 3.04 | 4.35 | 0.2825478 | 1 |
| brain development | 2.06 | 3.11 | 0.2871908 | 1 |
| ectoderm development | 1.84 | 0.93 | 0.315395 | 1 |
| hexose metabolic process | 1.84 | 0.93 | 0.315395 | 1 |
| microtubule cytoskeleton organization and biogenesis | 1.19 | 0.31 | 0.3167569 | 1 |
| positive regulation of immune response | 1.19 | 0.31 | 0.3167569 | 1 |
| metal ion transport | 5.31 | 6.83 | 0.3291208 | 1 |
| ubiquitin cycle | 4.45 | 3.11 | 0.3317314 | 1 |
| dopamine receptor signaling pathway | 0.54 | 0 | 0.3356444 | 1 |
| pyridine nucleotide metabolic process | 0.54 | 0 | 0.3356444 | 1 |
| programmed cell death | 8.35 | 6.52 | 0.3371278 | 1 |
| phospholipid metabolic process | 0.87 | 1.55 | 0.3393898 | 1 |
| glycosaminoglycan metabolic process | 0.43 | 0.93 | 0.383902 | 1 |
| gonad development | 0.43 | 0.93 | 0.383902 | 1 |
| gliogenesis | 0.43 | 0.93 | 0.383902 | 1 |
| di-, tri-valent inorganic cation transport | 2.17 | 3.11 | 0.3977976 | 1 |
| negative regulation of nucleobase, nucleoside, nucleotid | 4.56 | 3.42 | 0.4273414 | 1 |
| eye development | 0.54 | 0.93 | 0.4337513 | 1 |
| calcium-mediated signaling | 0.54 | 0.93 | 0.4337513 | 1 |
| regulation of synaptic transmission | 0.54 | 0.93 | 0.4337513 | 1 |

| | | | | |
|---|---|---|---|---|
| lipoprotein biosynthetic process | 0.54 | 0.93 | 0.4337513 | 1 |
| regulation of protein catabolic process | 0.11 | 0.31 | 0.4508398 | 1 |
| T-helper 1 type immune response | 0.11 | 0.31 | 0.4508398 | 1 |
| steroid catabolic process | 0.11 | 0.31 | 0.4508398 | 1 |
| S phase | 0.11 | 0.31 | 0.4508398 | 1 |
| regulation of smoothened signaling pathway | 0.11 | 0.31 | 0.4508398 | 1 |
| cytolysis | 0.11 | 0.31 | 0.4508398 | 1 |
| polyisoprenoid metabolic process | 0.11 | 0.31 | 0.4508398 | 1 |
| lymphocyte activation during immune response | 0.11 | 0.31 | 0.4508398 | 1 |
| myeloid leukocyte mediated immunity | 0.11 | 0.31 | 0.4508398 | 1 |
| regulation of macrophage activation | 0.11 | 0.31 | 0.4508398 | 1 |
| glycerolipid biosynthetic process | 0.11 | 0.31 | 0.4508398 | 1 |
| neutral lipid biosynthetic process | 0.11 | 0.31 | 0.4508398 | 1 |
| Mo-molybdopterin cofactor metabolic process | 0.11 | 0.31 | 0.4508398 | 1 |
| blastoderm segmentation | 0.11 | 0.31 | 0.4508398 | 1 |
| leukocyte degranulation | 0.11 | 0.31 | 0.4508398 | 1 |
| formation of primary germ layer | 0.11 | 0.31 | 0.4508398 | 1 |
| positive regulation of fibroblast proliferation | 0.11 | 0.31 | 0.4508398 | 1 |
| cardiac cell differentiation | 0.11 | 0.31 | 0.4508398 | 1 |
| sulfur amino acid metabolic process | 0.11 | 0.31 | 0.4508398 | 1 |
| molybdopterin cofactor biosynthetic process | 0.11 | 0.31 | 0.4508398 | 1 |
| neuromuscular synaptic transmission | 0.11 | 0.31 | 0.4508398 | 1 |
| positive regulation of cell migration | 0.11 | 0.31 | 0.4508398 | 1 |
| regulation of RNA metabolic process | 0.11 | 0.31 | 0.4508398 | 1 |
| lipoprotein catabolic process | 0.11 | 0.31 | 0.4508398 | 1 |
| retinoid metabolic process | 0.11 | 0.31 | 0.4508398 | 1 |
| retinoic acid metabolic process | 0.11 | 0.31 | 0.4508398 | 1 |
| lipopolysaccharide metabolic process | 0.11 | 0.31 | 0.4508398 | 1 |
| peptide hormone secretion | 0.11 | 0.31 | 0.4508398 | 1 |
| nuclear transport | 1.52 | 2.17 | 0.4528719 | 1 |
| steroid hormone receptor signaling pathway | 0.87 | 0.31 | 0.4605045 | 1 |
| monosaccharide catabolic process | 0.98 | 0.31 | 0.4680323 | 1 |
| nucleocytoplasmic transport | 1.63 | 2.17 | 0.4732204 | 1 |
| generation of neurons | 5.97 | 4.66 | 0.482285 | 1 |
| regulation of transcription | 22.23 | 24.22 | 0.4872453 | 1 |
| regulation of cell shape | 0.76 | 1.24 | 0.4890871 | 1 |

| | | | | |
|---|---|---|---|---|
| regulation of T cell activation | 0.76 | 1.24 | 0.4890871 | 1 |
| metanephros development | 0.76 | 1.24 | 0.4890871 | 1 |
| phosphoinositide-mediated signaling | 1.08 | 1.55 | 0.5539047 | 1 |
| Ras protein signal transduction | 2.93 | 2.17 | 0.5560774 | 1 |
| regulation of epithelial cell differentiation | 0.33 | 0 | 0.5728754 | 1 |
| aerobic respiration | 0.33 | 0 | 0.5728754 | 1 |
| positive regulation of protein secretion | 0.33 | 0 | 0.5728754 | 1 |
| regulation of chemotaxis | 0.33 | 0 | 0.5728754 | 1 |
| positive chemotaxis | 0.33 | 0 | 0.5728754 | 1 |
| organic anion transport | 0.33 | 0 | 0.5728754 | 1 |
| pinocytosis | 0.33 | 0 | 0.5728754 | 1 |
| positive regulation of progression through cell cycle | 0.33 | 0 | 0.5728754 | 1 |
| serine family amino acid metabolic process | 0.33 | 0 | 0.5728754 | 1 |
| pyruvate metabolic process | 0.33 | 0 | 0.5728754 | 1 |
| unfolded protein response | 0.33 | 0 | 0.5728754 | 1 |
| folic acid and derivative metabolic process | 0.33 | 0 | 0.5728754 | 1 |
| monovalent inorganic cation homeostasis | 0.33 | 0 | 0.5728754 | 1 |
| somitogenesis | 0.33 | 0 | 0.5728754 | 1 |
| monosaccharide biosynthetic process | 0.33 | 0 | 0.5728754 | 1 |
| peptide cross-linking | 0.33 | 0 | 0.5728754 | 1 |
| regulation of cytokine secretion | 0.33 | 0 | 0.5728754 | 1 |
| oligosaccharide biosynthetic process | 0.33 | 0 | 0.5728754 | 1 |
| amino acid activation | 0.33 | 0 | 0.5728754 | 1 |
| gastrulation (sensu Deuterostomia) | 0.33 | 0 | 0.5728754 | 1 |
| oxidative phosphorylation | 0.33 | 0 | 0.5728754 | 1 |
| protein tetramerization | 0.33 | 0 | 0.5728754 | 1 |
| ribonucleoside monophosphate metabolic process | 0.33 | 0 | 0.5728754 | 1 |
| nucleoside monophosphate biosynthetic process | 0.33 | 0 | 0.5728754 | 1 |
| mitochondrial transport | 0.43 | 0 | 0.5778086 | 1 |
| regulation of Wnt receptor signaling pathway | 0.43 | 0 | 0.5778086 | 1 |
| negative regulation of cell size | 0.43 | 0 | 0.5778086 | 1 |
| base-excision repair | 0.43 | 0 | 0.5778086 | 1 |
| protein amino acid ADP-ribosylation | 0.43 | 0 | 0.5778086 | 1 |
| nucleotide-excision repair | 0.43 | 0 | 0.5778086 | 1 |
| biogenic amine biosynthetic process | 0.33 | 0.62 | 0.6088771 | 1 |
| ensheathment of neurons | 0.33 | 0.62 | 0.6088771 | 1 |

| | | | | |
|---|---|---|---|---|
| negative regulation of lymphocyte activation | 0.33 | 0.62 | 0.6088771 | 1 |
| regulation of endocytosis | 0.33 | 0.62 | 0.6088771 | 1 |
| gamma-aminobutyric acid signaling pathway | 0.33 | 0.62 | 0.6088771 | 1 |
| cell projection organization and biogenesis | 4.01 | 3.11 | 0.610386 | 1 |
| RNA splicing | 1.95 | 1.24 | 0.6229159 | 1 |
| protein amino acid dephosphorylation | 1.95 | 2.48 | 0.6505829 | 1 |
| translational initiation | 0.43 | 0.62 | 0.6522511 | 1 |
| amino acid catabolic process | 0.43 | 0.62 | 0.6522511 | 1 |
| mesoderm development | 0.43 | 0.62 | 0.6522511 | 1 |
| steroid biosynthetic process | 0.43 | 0.62 | 0.6522511 | 1 |
| cell cycle checkpoint | 0.65 | 0.31 | 0.6844136 | 1 |
| determination of left/right symmetry | 0.65 | 0.31 | 0.6844136 | 1 |
| cartilage development | 0.65 | 0.31 | 0.6844136 | 1 |
| regulation of DNA metabolic process | 0.65 | 0.31 | 0.6844136 | 1 |
| leukocyte chemotaxis | 0.76 | 0.31 | 0.6880704 | 1 |
| hemopoietic or lymphoid organ development | 3.04 | 3.42 | 0.7128128 | 1 |
| blood vessel development | 3.36 | 2.8 | 0.7159592 | 1 |
| membrane lipid biosynthetic process | 0.76 | 0.93 | 0.7247405 | 1 |
| positive regulation of cellular biosynthetic process | 0.76 | 0.93 | 0.7247405 | 1 |
| actin cytoskeleton organization and biogenesis | 3.8 | 3.11 | 0.7288196 | 1 |
| ribonucleotide biosynthetic process | 0.98 | 0.62 | 0.738273 | 1 |
| transmembrane receptor protein serine/threonine kinase | 1.08 | 0.62 | 0.7413155 | 1 |
| embryonic limb morphogenesis | 1.08 | 0.62 | 0.7413155 | 1 |
| antimicrobial humoral response (sensu Vertebrata) | 0.98 | 1.24 | 0.7507852 | 1 |
| transcription, DNA-dependent | 21.69 | 22.67 | 0.7544534 | 1 |
| cytokine biosynthetic process | 1.08 | 1.24 | 0.7650119 | 1 |
| neuropeptide signaling pathway | 1.3 | 1.55 | 0.7811147 | 1 |
| striated muscle development | 1.63 | 1.86 | 0.8024785 | 1 |
| di-, tri-valent inorganic cation homeostasis | 1.74 | 1.86 | 0.8106393 | 1 |
| cyclic-nucleotide-mediated signaling | 1.95 | 1.55 | 0.8116982 | 1 |
| establishment and/or maintenance of chromatin architec | 2.6 | 2.17 | 0.8360549 | 1 |
| transmembrane receptor protein tyrosine kinase signalin | 2.93 | 3.11 | 0.85016 | 1 |
| tRNA processing | 0.33 | 0.31 | 1 | 1 |
| synaptic vesicle endocytosis | 0.11 | 0 | 1 | 1 |
| JAK-STAT cascade | 0.43 | 0.31 | 1 | 1 |
| pyrimidine ribonucleotide metabolic process | 0.22 | 0.31 | 1 | 1 |

| | | | | |
|---|---|---|---|---|
| tissue regeneration | 0.11 | 0 | 1 | 1 |
| negative regulation of endothelial cell proliferation | 0.22 | 0.31 | 1 | 1 |
| long-chain fatty acid transport | 0.11 | 0 | 1 | 1 |
| ribonucleoside triphosphate metabolic process | 0.54 | 0.62 | 1 | 1 |
| regulation of melanocyte differentiation | 0.11 | 0 | 1 | 1 |
| mRNA catabolic process | 0.11 | 0 | 1 | 1 |
| monovalent inorganic cation transport | 3.69 | 3.73 | 1 | 1 |
| posttranslational protein folding | 0.22 | 0 | 1 | 1 |
| pyridoxine metabolic process | 0.11 | 0 | 1 | 1 |
| mRNA modification | 0.11 | 0 | 1 | 1 |
| lipid raft distribution | 0.11 | 0 | 1 | 1 |
| mesonephros development | 0.11 | 0 | 1 | 1 |
| pyrimidine base biosynthetic process | 0.22 | 0 | 1 | 1 |
| MAPKKK cascade | 1.84 | 1.86 | 1 | 1 |
| heme oxidation | 0.11 | 0 | 1 | 1 |
| mammary gland development | 0.22 | 0 | 1 | 1 |
| hexose transport | 0.33 | 0.31 | 1 | 1 |
| pyrimidine nucleotide biosynthetic process | 0.33 | 0.31 | 1 | 1 |
| chemokine metabolic process | 0.11 | 0 | 1 | 1 |
| thiamin metabolic process | 0.11 | 0 | 1 | 1 |
| regulation of G-protein coupled receptor protein signaling | 0.54 | 0.31 | 1 | 1 |
| regulation of translation | 1.52 | 1.55 | 1 | 1 |
| positive regulation of smooth muscle cell proliferation | 0.11 | 0 | 1 | 1 |
| Golgi vesicle transport | 0.87 | 0.62 | 1 | 1 |
| negative regulation of cellular biosynthetic process | 0.43 | 0.31 | 1 | 1 |
| ubiquinone metabolic process | 0.11 | 0 | 1 | 1 |
| odontogenesis | 0.33 | 0.31 | 1 | 1 |
| M phase of meiotic cell cycle | 0.76 | 0.62 | 1 | 1 |
| negative regulation of fibroblast proliferation | 0.11 | 0 | 1 | 1 |
| interphase of mitotic cell cycle | 0.87 | 0.62 | 1 | 1 |
| glycerol-3-phosphate metabolic process | 0.11 | 0 | 1 | 1 |
| G2 phase | 0.22 | 0 | 1 | 1 |
| entry into host | 0.22 | 0 | 1 | 1 |
| negative regulation of epithelial cell proliferation | 0.22 | 0 | 1 | 1 |
| regulation of sensory perception of pain | 0.11 | 0 | 1 | 1 |
| I-kappaB kinase/NF-kappaB cascade | 1.84 | 1.86 | 1 | 1 |

| | | | | |
|---|---|---|---|---|
| cytoskeleton-dependent intracellular transport | 1.41 | 1.24 | 1 | 1 |
| androgen metabolic process | 0.22 | 0.31 | 1 | 1 |
| regulation of progression through mitotic cell cycle | 0.22 | 0.31 | 1 | 1 |
| pyrimidine nucleoside triphosphate metabolic process | 0.22 | 0.31 | 1 | 1 |
| embryonic epithelial tube formation | 0.43 | 0.31 | 1 | 1 |
| muscle maintenance | 0.22 | 0 | 1 | 1 |
| embryonic skeletal development | 0.11 | 0 | 1 | 1 |
| single strand break repair | 0.22 | 0 | 1 | 1 |
| regulation of antiviral response by host | 0.11 | 0 | 1 | 1 |
| protein amino acid alkylation | 0.22 | 0 | 1 | 1 |
| protein processing | 1.08 | 0.93 | 1 | 1 |
| regulation of neurotransmitter levels | 1.74 | 1.55 | 1 | 1 |
| protein amino acid deacetylation | 0.33 | 0.31 | 1 | 1 |
| pyrimidine transport | 0.11 | 0 | 1 | 1 |
| positive regulation of protein complex assembly | 0.11 | 0 | 1 | 1 |
| nucleoside triphosphate biosynthetic process | 0.54 | 0.62 | 1 | 1 |
| thyroid hormone metabolic process | 0.11 | 0 | 1 | 1 |
| regulation of innate immune response | 0.11 | 0 | 1 | 1 |
| regulation of protein modification | 0.11 | 0 | 1 | 1 |
| ribonucleoside diphosphate metabolic process | 0.11 | 0 | 1 | 1 |
| pyrimidine nucleoside catabolic process | 0.11 | 0 | 1 | 1 |
| cell killing | 0.33 | 0.31 | 1 | 1 |
| mitochondrial DNA metabolic process | 0.11 | 0 | 1 | 1 |
| ethanolamine and derivative metabolic process | 0.22 | 0 | 1 | 1 |
| C21-steroid hormone metabolic process | 0.11 | 0 | 1 | 1 |
| alpha-beta T cell activation | 0.43 | 0.31 | 1 | 1 |
| negative regulation of fat cell differentiation | 0.11 | 0 | 1 | 1 |
| entry into cell of other organism during symbiotic interac | 0.22 | 0 | 1 | 1 |
| leg morphogenesis | 0.11 | 0 | 1 | 1 |
| hemoglobin biosynthetic process | 0.22 | 0 | 1 | 1 |
| RNA-mediated posttranscriptional gene silencing | 0.11 | 0 | 1 | 1 |
| activation of plasma proteins during acute inflammatory | 0.43 | 0.31 | 1 | 1 |
| metal ion homeostasis | 1.84 | 1.86 | 1 | 1 |
| B cell proliferation | 0.33 | 0.31 | 1 | 1 |
| anterior/posterior axis specification | 0.11 | 0 | 1 | 1 |
| neurotransmitter receptor metabolic process | 0.11 | 0 | 1 | 1 |

| | | | | |
|---|---|---|---|---|
| neural tube patterning | 0.11 | 0 | 1 | 1 |
| regulation of leukocyte mediated immunity | 0.11 | 0 | 1 | 1 |
| regulation of amino acid metabolic process | 0.54 | 0.62 | 1 | 1 |
| vesicle docking during exocytosis | 0.43 | 0.31 | 1 | 1 |
| adrenal gland development | 0.11 | 0 | 1 | 1 |
| hindlimb morphogenesis | 0.22 | 0 | 1 | 1 |
| regulation of exocytosis | 0.33 | 0.31 | 1 | 1 |
| positive regulation of fat cell differentiation | 0.11 | 0 | 1 | 1 |
| serotonin receptor signaling pathway | 0.11 | 0 | 1 | 1 |
| purine nucleoside diphosphate metabolic process | 0.11 | 0 | 1 | 1 |
| receptor-mediated endocytosis | 0.87 | 0.62 | 1 | 1 |
| positive regulation of immune effector process | 0.11 | 0 | 1 | 1 |
| pentose metabolic process | 0.11 | 0 | 1 | 1 |
| protein amino acid acylation | 0.22 | 0 | 1 | 1 |
| regulation of nucleotide metabolic process | 0.22 | 0.31 | 1 | 1 |
| skeletal morphogenesis | 0.11 | 0 | 1 | 1 |
| antigen processing and presentation, exogenous lipid ar | 0.11 | 0 | 1 | 1 |
| T-helper 2 type immune response | 0.11 | 0 | 1 | 1 |
| embryonic gut development | 0.22 | 0.31 | 1 | 1 |
| glucosamine metabolic process | 0.22 | 0 | 1 | 1 |
| ectodermal gut development | 0.22 | 0 | 1 | 1 |
| digestive tract morphogenesis | 0.22 | 0.31 | 1 | 1 |
| rRNA processing | 0.22 | 0.31 | 1 | 1 |
| menstrual cycle phase | 0.22 | 0.31 | 1 | 1 |
| estrogen metabolic process | 0.22 | 0.31 | 1 | 1 |
| oocyte differentiation | 0.22 | 0 | 1 | 1 |
| negative regulation of protein transport | 0.22 | 0 | 1 | 1 |
| somatic cell DNA recombination | 0.22 | 0 | 1 | 1 |
| sphingolipid metabolic process | 0.76 | 0.62 | 1 | 1 |
| indole derivative biosynthetic process | 0.11 | 0 | 1 | 1 |
| establishment of nucleus localization | 0.11 | 0 | 1 | 1 |
| forelimb morphogenesis | 0.22 | 0 | 1 | 1 |
| phospholipid dephosphorylation | 0.22 | 0 | 1 | 1 |
| tubulin folding | 0.22 | 0 | 1 | 1 |
| lipid glycosylation | 0.11 | 0 | 1 | 1 |
| acetyl-CoA catabolic process | 0.22 | 0 | 1 | 1 |

| | | | | |
|---|---|---|---|---|
| synaptic transmission, cholinergic | 0.11 | 0 | 1 | 1 |
| vacuolar transport | 0.33 | 0.31 | 1 | 1 |
| embryonic organ morphogenesis | 0.33 | 0.31 | 1 | 1 |
| ATP-dependent proteolysis | 0.11 | 0 | 1 | 1 |
| sterol metabolic process | 0.54 | 0.62 | 1 | 1 |
| positive regulation of protein transport | 0.11 | 0 | 1 | 1 |
| cholesterol transport | 0.22 | 0 | 1 | 1 |
| establishment of vesicle localization | 0.22 | 0.31 | 1 | 1 |
| germ-line stem cell division | 0.11 | 0 | 1 | 1 |
| endosome transport | 0.54 | 0.31 | 1 | 1 |
| glycolate metabolic process | 0.11 | 0 | 1 | 1 |
| telomere organization and biogenesis | 0.11 | 0 | 1 | 1 |
| dorsal/ventral axis specification | 0.11 | 0 | 1 | 1 |
| amino acid transport | 0.33 | 0.31 | 1 | 1 |
| regulation of lipid kinase activity | 0.22 | 0 | 1 | 1 |
| gonadotropin secretion | 0.11 | 0 | 1 | 1 |
| purine nucleotide biosynthetic process | 0.76 | 0.62 | 1 | 1 |
| anaphase | 0.11 | 0 | 1 | 1 |
| rRNA modification | 0.11 | 0 | 1 | 1 |
| regulation of synapse structure and activity | 0.22 | 0 | 1 | 1 |
| negative regulation of protein secretion | 0.11 | 0 | 1 | 1 |
| purine base biosynthetic process | 0.11 | 0 | 1 | 1 |
| peptidyl-amino acid modification | 1.52 | 1.55 | 1 | 1 |
| positive regulation of inflammatory response | 0.11 | 0 | 1 | 1 |
| coenzyme A metabolic process | 0.11 | 0 | 1 | 1 |
| oogenesis (sensu Mammalia) | 0.11 | 0 | 1 | 1 |
| interleukin-1 secretion | 0.22 | 0 | 1 | 1 |
| protein kinase B signaling cascade | 0.11 | 0 | 1 | 1 |
| nucleoside phosphate metabolic process | 0.33 | 0.31 | 1 | 1 |
| frizzled-2 signaling pathway | 0.11 | 0 | 1 | 1 |
| protein maturation via proteolysis | 0.11 | 0 | 1 | 1 |
| nucleoside diphosphate biosynthetic process | 0.11 | 0 | 1 | 1 |
| sister chromatid segregation | 0.22 | 0.31 | 1 | 1 |
| disruption by symbiont of host cells | 0.11 | 0 | 1 | 1 |
| gamma-delta T cell activation | 0.11 | 0 | 1 | 1 |
| catecholamine metabolic process | 0.54 | 0.62 | 1 | 1 |

| | | | | |
|---|---|---|---|---|
| RNA 3'-end processing | 0.22 | 0 | 1 | 1 |
| regulation of small GTPase mediated signal transductior | 2.17 | 2.17 | 1 | 1 |
| uracil metabolic process | 0.11 | 0 | 1 | 1 |
| translational elongation | 0.11 | 0 | 1 | 1 |
| negative regulation of inflammatory response | 0.22 | 0 | 1 | 1 |
| acetylcholine receptor signaling, muscarinic pathway | 0.11 | 0 | 1 | 1 |
| cAMP metabolic process | 0.22 | 0.31 | 1 | 1 |
| isoprenoid biosynthetic process | 0.11 | 0 | 1 | 1 |
| regulation of cytokine and chemokine mediated signaling | 0.11 | 0 | 1 | 1 |
| indolalkylamine metabolic process | 0.11 | 0 | 1 | 1 |
| positive regulation of GTPase activity | 0.11 | 0 | 1 | 1 |
| positive regulation of response to tumor cell | 0.11 | 0 | 1 | 1 |
| polarized epithelial cell differentiation | 0.11 | 0 | 1 | 1 |
| di-, tri-valent inorganic anion homeostasis | 0.11 | 0 | 1 | 1 |
| M phase of mitotic cell cycle | 1.63 | 1.55 | 1 | 1 |
| spermatid differentiation | 0.11 | 0 | 1 | 1 |
| protein modification by small protein conjugation | 0.65 | 0.62 | 1 | 1 |
| re-entry into mitotic cell cycle | 0.22 | 0 | 1 | 1 |
| regulation of lymphocyte proliferation | 0.65 | 0.62 | 1 | 1 |
| thiamin diphosphate metabolic process | 0.11 | 0 | 1 | 1 |
| thiamin and derivative biosynthetic process | 0.11 | 0 | 1 | 1 |
| RNA transport | 0.43 | 0.31 | 1 | 1 |
| deoxyribonucleoside catabolic process | 0.11 | 0 | 1 | 1 |
| development of primary male sexual characteristics | 0.33 | 0.31 | 1 | 1 |
| somatic diversification of immune receptors | 0.22 | 0.31 | 1 | 1 |
| neural plate morphogenesis | 0.33 | 0.31 | 1 | 1 |
| tricarboxylic acid cycle intermediate metabolic process | 0.43 | 0.31 | 1 | 1 |
| purine transport | 0.11 | 0 | 1 | 1 |
| chondroitin sulfate proteoglycan metabolic process | 0.11 | 0 | 1 | 1 |
| metaphase | 0.11 | 0 | 1 | 1 |
| organic cation transport | 0.22 | 0 | 1 | 1 |
| neural crest cell development | 0.22 | 0 | 1 | 1 |
| regulation of blood coagulation | 0.22 | 0 | 1 | 1 |
| regulation of smooth muscle cell differentiation | 0.11 | 0 | 1 | 1 |
| oogenesis stage | 0.11 | 0 | 1 | 1 |
| purine ribonucleotide metabolic process | 0.65 | 0.62 | 1 | 1 |

| | | | | |
|---|---|---|---|---|
| butyrate metabolic process | 0.11 | 0 | 1 | 1 |
| lymphocyte mediated immunity | 0.76 | 0.62 | 1 | 1 |
| PML body organization and biogenesis | 0.11 | 0 | 1 | 1 |
| positive regulation of lymphocyte activation | 0.65 | 0.62 | 1 | 1 |
| regulation of hormone secretion | 0.11 | 0 | 1 | 1 |
| positive regulation of vasodilation | 0.11 | 0 | 1 | 1 |
| protein amino acid sulfation | 0.22 | 0 | 1 | 1 |
| uroporphyrinogen III biosynthetic process | 0.11 | 0 | 1 | 1 |
| protein stabilization | 0.22 | 0 | 1 | 1 |
| platelet activation | 0.11 | 0 | 1 | 1 |
| negative regulation of muscle cell differentiation | 0.11 | 0 | 1 | 1 |
| regulation of B cell activation | 0.54 | 0.31 | 1 | 1 |
| tissue morphogenesis | 0.76 | 0.62 | 1 | 1 |
| positive regulation of ion transport | 0.11 | 0 | 1 | 1 |
| lung development | 0.54 | 0.62 | 1 | 1 |
| histidine family amino acid metabolic process | 0.22 | 0.31 | 1 | 1 |
| protein heterooligomerization | 0.11 | 0 | 1 | 1 |
| establishment of cell polarity | 0.22 | 0 | 1 | 1 |
| midgut development | 0.11 | 0 | 1 | 1 |
| pyrimidine nucleotide-sugar transport | 0.11 | 0 | 1 | 1 |
| membrane protein ectodomain proteolysis | 0.11 | 0 | 1 | 1 |
| modification of cellular component in other organism dur | 0.11 | 0 | 1 | 1 |
| pyrimidine base catabolic process | 0.11 | 0 | 1 | 1 |
| sister chromatid cohesion | 0.22 | 0 | 1 | 1 |
| aromatic amino acid family metabolic process | 0.22 | 0.31 | 1 | 1 |
| RNA editing | 0.22 | 0 | 1 | 1 |
| benzoate metabolic process | 0.11 | 0 | 1 | 1 |
| phagocytosis | 0.33 | 0.31 | 1 | 1 |
| specification of organ identity | 0.22 | 0.31 | 1 | 1 |
| T cell proliferation | 0.98 | 0.93 | 1 | 1 |
| modification by symbiont of host structure | 0.11 | 0 | 1 | 1 |
| vitamin B6 biosynthetic process | 0.11 | 0 | 1 | 1 |
| formate metabolic process | 0.11 | 0 | 1 | 1 |
| tricarboxylic acid transport | 0.11 | 0 | 1 | 1 |
| branched chain family amino acid metabolic process | 0.22 | 0 | 1 | 1 |
| conditioned taste aversion | 0.11 | 0 | 1 | 1 |

| | | | | |
|---|---|---|---|---|
| beta-amyloid metabolic process | 0.11 | 0 | 1 | 1 |
| cyclic nucleotide catabolic process | 0.11 | 0 | 1 | 1 |
| regulation of carbohydrate biosynthetic process | 0.11 | 0 | 1 | 1 |
| nerve-nerve synaptic transmission | 0.33 | 0.31 | 1 | 1 |
| immunoglobulin production | 0.22 | 0.31 | 1 | 1 |
| purine ribonucleoside metabolic process | 0.22 | 0 | 1 | 1 |
| pyrimidine deoxyribonucleoside metabolic process | 0.11 | 0 | 1 | 1 |
| segment specification | 0.22 | 0 | 1 | 1 |
| regulation of phosphate metabolic process | 0.76 | 0.62 | 1 | 1 |
| monocarboxylic acid transport | 0.11 | 0 | 1 | 1 |
| establishment and/or maintenance of apical/basal cell po | 0.11 | 0 | 1 | 1 |
| quinone cofactor biosynthetic process | 0.11 | 0 | 1 | 1 |
| regulation of immune response to tumor cell | 0.11 | 0 | 1 | 1 |
| purine nucleoside triphosphate metabolic process | 0.54 | 0.62 | 1 | 1 |

| Gene Ontology : biological process. Level: 8 | Percentage of genes with allele-specific expression | Percentage of genes without allele-specific expression | Unadjusted pvalue | Adjusted pvalueFDR |
|---|---|---|---|---|
| ossification | 0.97 | 3.45 | 0.01768197 | 1 |
| proteolysis involved in cellular protein catabolic process | 2.36 | 4.98 | 0.0559508 | 1 |
| activation of phospholipase A2 | 0 | 0.77 | 0.07058603 | 1 |
| double-strand break repair via homologous recombinatic | 0 | 0.77 | 0.07058603 | 1 |
| heparan sulfate proteoglycan biosynthetic process | 0 | 0.77 | 0.07058603 | 1 |
| DNA strand elongation during DNA replication | 0 | 0.77 | 0.07058603 | 1 |
| protein targeting | 2.92 | 5.36 | 0.07934332 | 1 |
| positive regulation of transcription | 5.42 | 2.68 | 0.08691233 | 1 |
| skeletal muscle development | 0.83 | 2.3 | 0.09400779 | 1 |
| regulation of programmed cell death | 7.78 | 4.98 | 0.1574026 | 1 |
| cell cycle arrest | 1.39 | 2.68 | 0.1738405 | 1 |
| BMP signaling pathway | 0.14 | 0.77 | 0.1744102 | 1 |
| detection of light stimulus during visual perception | 0.14 | 0.77 | 0.1744102 | 1 |
| activated T cell proliferation | 0.14 | 0.77 | 0.1744102 | 1 |
| catecholamine biosynthetic process | 0.14 | 0.77 | 0.1744102 | 1 |
| diacylglycerol metabolic process | 0.14 | 0.77 | 0.1744102 | 1 |
| glycolipid catabolic process | 0.14 | 0.77 | 0.1744102 | 1 |
| positive regulation of small GTPase mediated signal trar | 0.14 | 0.77 | 0.1744102 | 1 |
| cGMP biosynthetic process | 0.14 | 0.77 | 0.1744102 | 1 |
| epidermis development | 2.22 | 0.77 | 0.1800566 | 1 |
| regulation of cyclin-dependent protein kinase activity | 0.42 | 1.15 | 0.195049 | 1 |
| regulation of synaptic plasticity | 0.42 | 1.15 | 0.195049 | 1 |
| glial cell differentiation | 0.42 | 1.15 | 0.195049 | 1 |
| intracellular protein transport across a membrane | 0.42 | 1.15 | 0.195049 | 1 |
| sodium ion transport | 1.53 | 0.38 | 0.1993123 | 1 |
| inner ear development | 0.56 | 1.53 | 0.219653 | 1 |
| apoptosis | 10.56 | 7.66 | 0.2235061 | 1 |
| neuron differentiation | 6.81 | 4.6 | 0.2334345 | 1 |
| phosphate transport | 1.25 | 2.3 | 0.2451004 | 1 |
| protein amino acid glycosylation | 2.92 | 1.53 | 0.2607733 | 1 |
| regulation of cell growth | 2.08 | 0.77 | 0.2657904 | 1 |
| positive regulation of Notch signaling pathway | 0 | 0.38 | 0.266055 | 1 |
| asparagine metabolic process | 0 | 0.38 | 0.266055 | 1 |
| regulation of DNA ligation | 0 | 0.38 | 0.266055 | 1 |
| phagocytosis, recognition | 0 | 0.38 | 0.266055 | 1 |

| | | | | |
|---|---|---|---|---|
| vasoconstriction of artery during baroreceptor response | 0 | 0.38 | 0.266055 | 1 |
| inositol phosphate-mediated signaling | 0 | 0.38 | 0.266055 | 1 |
| low-density lipoprotein catabolic process | 0 | 0.38 | 0.266055 | 1 |
| bile acid biosynthetic process | 0 | 0.38 | 0.266055 | 1 |
| mesoderm morphogenesis | 0 | 0.38 | 0.266055 | 1 |
| fatty acid desaturation | 0 | 0.38 | 0.266055 | 1 |
| actomyosin structure organization and biogenesis | 0 | 0.38 | 0.266055 | 1 |
| bile acid catabolic process | 0 | 0.38 | 0.266055 | 1 |
| transcription from RNA polymerase I promoter | 0 | 0.38 | 0.266055 | 1 |
| regulation of rhodopsin gene activity | 0 | 0.38 | 0.266055 | 1 |
| catecholamine catabolic process | 0 | 0.38 | 0.266055 | 1 |
| platelet activating factor metabolic process | 0 | 0.38 | 0.266055 | 1 |
| DNA replication initiation | 0 | 0.38 | 0.266055 | 1 |
| aspartate family amino acid biosynthetic process | 0 | 0.38 | 0.266055 | 1 |
| phospholipid catabolic process | 0 | 0.38 | 0.266055 | 1 |
| phospholipid transfer to membrane | 0 | 0.38 | 0.266055 | 1 |
| antibacterial humoral response (sensu Vertebrata) | 0 | 0.38 | 0.266055 | 1 |
| centrosome separation | 0 | 0.38 | 0.266055 | 1 |
| protein deglycosylation | 0 | 0.38 | 0.266055 | 1 |
| glucan catabolic process | 0 | 0.38 | 0.266055 | 1 |
| ventricular system development | 0 | 0.38 | 0.266055 | 1 |
| increased strength of heart contraction by epinephrine-n | 0 | 0.38 | 0.266055 | 1 |
| inductive cell-cell signaling | 0 | 0.38 | 0.266055 | 1 |
| double-strand break repair via nonhomologous end joini | 0 | 0.38 | 0.266055 | 1 |
| DNA unwinding during replication | 0 | 0.38 | 0.266055 | 1 |
| positive regulation of vesicle fusion | 0 | 0.38 | 0.266055 | 1 |
| periodic partitioning | 0 | 0.38 | 0.266055 | 1 |
| positive regulation of urothelial cell proliferation | 0 | 0.38 | 0.266055 | 1 |
| transcription from mitochondrial promoter | 0 | 0.38 | 0.266055 | 1 |
| antifungal humoral response (sensu Vertebrata) | 0 | 0.38 | 0.266055 | 1 |
| positive regulation of heart contraction rate by epinephri | 0 | 0.38 | 0.266055 | 1 |
| DNA damage induced protein phosphorylation | 0 | 0.38 | 0.266055 | 1 |
| striated muscle cell differentiation | 0 | 0.38 | 0.266055 | 1 |
| protein deubiquitination | 0 | 0.38 | 0.266055 | 1 |
| galactolipid metabolic process | 0 | 0.38 | 0.266055 | 1 |
| sterol catabolic process | 0 | 0.38 | 0.266055 | 1 |

| | | | | |
|---|---|---|---|---|
| extracellular polysaccharide biosynthetic process | 0 | 0.38 | 0.266055 | 1 |
| lipopolysaccharide biosynthetic process | 0 | 0.38 | 0.266055 | 1 |
| negative regulation of macrophage activation | 0 | 0.38 | 0.266055 | 1 |
| transcription from RNA polymerase II promoter | 10.28 | 7.66 | 0.2689963 | 1 |
| sphingolipid catabolic process | 0.28 | 0.77 | 0.2889035 | 1 |
| glycosaminoglycan biosynthetic process | 0.28 | 0.77 | 0.2889035 | 1 |
| negative regulation of T cell activation | 0.28 | 0.77 | 0.2889035 | 1 |
| chloride transport | 0.28 | 0.77 | 0.2889035 | 1 |
| regulation of transcription factor activity | 0.28 | 0.77 | 0.2889035 | 1 |
| glycerophospholipid metabolic process | 0.28 | 0.77 | 0.2889035 | 1 |
| nuclear export | 0.28 | 0.77 | 0.2889035 | 1 |
| glutamine metabolic process | 0.28 | 0.77 | 0.2889035 | 1 |
| glycogen metabolic process | 0.28 | 0.77 | 0.2889035 | 1 |
| female gonad development | 0.28 | 0.77 | 0.2889035 | 1 |
| post-Golgi vesicle-mediated transport | 0.28 | 0.77 | 0.2889035 | 1 |
| eye development (sensu Vertebrata) | 0.28 | 0.77 | 0.2889035 | 1 |
| hexose catabolic process | 1.25 | 0.38 | 0.3055197 | 1 |
| chromatin assembly or disassembly | 0.97 | 1.92 | 0.3204053 | 1 |
| G-protein signaling, coupled to IP3 second messenger ( | 0.97 | 1.92 | 0.3204053 | 1 |
| interleukin-6 biosynthetic process | 0.69 | 0 | 0.3328018 | 1 |
| transforming growth factor beta receptor signaling pathw | 0.69 | 0 | 0.3328018 | 1 |
| transcription initiation | 0.69 | 0 | 0.3328018 | 1 |
| positive regulation of protein kinase activity | 2.08 | 3.07 | 0.3491708 | 1 |
| actin filament organization | 0.83 | 0 | 0.3504891 | 1 |
| calcium ion transport | 2.22 | 3.45 | 0.3576195 | 1 |
| protein autoprocessing | 0.56 | 1.15 | 0.3909792 | 1 |
| icosanoid metabolic process | 0.56 | 1.15 | 0.3909792 | 1 |
| ear morphogenesis | 0.56 | 1.15 | 0.3909792 | 1 |
| negative regulation of protein kinase activity | 0.69 | 1.15 | 0.4449706 | 1 |
| protein amino acid lipidation | 0.69 | 1.15 | 0.4449706 | 1 |
| activation of immune response | 1.11 | 0.38 | 0.4582135 | 1 |
| glutamine family amino acid biosynthetic process | 0.14 | 0.38 | 0.4615241 | 1 |
| male gonad development | 0.14 | 0.38 | 0.4615241 | 1 |
| S phase of mitotic cell cycle | 0.14 | 0.38 | 0.4615241 | 1 |
| negative regulation of progression through mitotic cell cy | 0.14 | 0.38 | 0.4615241 | 1 |
| mast cell mediated immunity | 0.14 | 0.38 | 0.4615241 | 1 |

| | | | | |
|---|---|---|---|---|
| regulation of receptor mediated endocytosis | 0.14 | 0.38 | 0.4615241 | 1 |
| interferon-gamma biosynthetic process | 0.14 | 0.38 | 0.4615241 | 1 |
| regulation of gliogenesis | 0.14 | 0.38 | 0.4615241 | 1 |
| terpenoid metabolic process | 0.14 | 0.38 | 0.4615241 | 1 |
| insulin secretion | 0.14 | 0.38 | 0.4615241 | 1 |
| cAMP biosynthetic process | 0.14 | 0.38 | 0.4615241 | 1 |
| ARF protein signal transduction | 0.14 | 0.38 | 0.4615241 | 1 |
| intra-Golgi vesicle-mediated transport | 0.14 | 0.38 | 0.4615241 | 1 |
| insulin-like growth factor receptor signaling pathway | 0.14 | 0.38 | 0.4615241 | 1 |
| androgen biosynthetic process | 0.14 | 0.38 | 0.4615241 | 1 |
| Mo-molybdopterin cofactor biosynthetic process | 0.14 | 0.38 | 0.4615241 | 1 |
| regulation of JAK-STAT cascade | 0.14 | 0.38 | 0.4615241 | 1 |
| sulfur amino acid biosynthetic process | 0.14 | 0.38 | 0.4615241 | 1 |
| synaptic transmission, dopaminergic | 0.14 | 0.38 | 0.4615241 | 1 |
| T cell activation during immune response | 0.14 | 0.38 | 0.4615241 | 1 |
| Rac protein signal transduction | 0.14 | 0.38 | 0.4615241 | 1 |
| regulation of RNA stability | 0.14 | 0.38 | 0.4615241 | 1 |
| natural killer cell mediated immunity | 0.14 | 0.38 | 0.4615241 | 1 |
| aromatic amino acid family catabolic process | 0.14 | 0.38 | 0.4615241 | 1 |
| RNA splicing, via endonucleolytic cleavage and ligation | 0.14 | 0.38 | 0.4615241 | 1 |
| transcription from RNA polymerase III promoter | 0.14 | 0.38 | 0.4615241 | 1 |
| acidic amino acid transport | 0.14 | 0.38 | 0.4615241 | 1 |
| triacylglycerol metabolic process | 0.14 | 0.38 | 0.4615241 | 1 |
| L-phenylalanine metabolic process | 0.14 | 0.38 | 0.4615241 | 1 |
| acylglycerol biosynthetic process | 0.14 | 0.38 | 0.4615241 | 1 |
| G-protein signaling, coupled to cyclic nucleotide second | 2.5 | 1.53 | 0.4691396 | 1 |
| chromatin modification | 2.5 | 1.53 | 0.4691396 | 1 |
| ureteric bud development | 0.83 | 1.53 | 0.4703257 | 1 |
| negative regulation of transcription | 5.56 | 4.21 | 0.515103 | 1 |
| peptidyl-tyrosine modification | 1.11 | 1.53 | 0.5303746 | 1 |
| potassium ion transport | 3.06 | 3.83 | 0.544697 | 1 |
| tRNA aminoacylation | 0.42 | 0 | 0.5693293 | 1 |
| serine family amino acid biosynthetic process | 0.42 | 0 | 0.5693293 | 1 |
| ribonucleoside monophosphate biosynthetic process | 0.42 | 0 | 0.5693293 | 1 |
| gastrulation (sensu Vertebrata) | 0.42 | 0 | 0.5693293 | 1 |
| neurotransmitter uptake | 0.42 | 0 | 0.5693293 | 1 |

| | | | | |
|---|---|---|---|---|
| cortical actin cytoskeleton organization and biogenesis | 0.42 | 0 | 0.5693293 | 1 |
| positive regulation of cytokine secretion | 0.42 | 0 | 0.5693293 | 1 |
| regulation of positive chemotaxis | 0.42 | 0 | 0.5693293 | 1 |
| negative regulation of B cell activation | 0.42 | 0 | 0.5693293 | 1 |
| hexose biosynthetic process | 0.42 | 0 | 0.5693293 | 1 |
| maintenance of fidelity during DNA-dependent DNA repl | 0.42 | 0 | 0.5693293 | 1 |
| folic acid and derivative biosynthetic process | 0.42 | 0 | 0.5693293 | 1 |
| embryonic digit morphogenesis | 0.42 | 0 | 0.5693293 | 1 |
| positive regulation of chemotaxis | 0.42 | 0 | 0.5693293 | 1 |
| actin filament-based movement | 0.42 | 0 | 0.5693293 | 1 |
| pyridine nucleotide biosynthetic process | 0.42 | 0 | 0.5693293 | 1 |
| ER to Golgi vesicle-mediated transport | 0.56 | 0 | 0.57843 | 1 |
| DNA integrity checkpoint | 0.56 | 0 | 0.57843 | 1 |
| fucose metabolic process | 0.56 | 0 | 0.57843 | 1 |
| transition metal ion homeostasis | 0.56 | 0 | 0.57843 | 1 |
| nicotinamide metabolic process | 0.56 | 0 | 0.57843 | 1 |
| regulation of striated muscle development | 0.56 | 0 | 0.57843 | 1 |
| spindle organization and biogenesis | 0.56 | 0 | 0.57843 | 1 |
| cAMP-mediated signaling | 1.81 | 1.15 | 0.5801394 | 1 |
| nuclear import | 1.67 | 2.3 | 0.5901221 | 1 |
| calcium ion homeostasis | 1.81 | 2.3 | 0.6053717 | 1 |
| sphingoid metabolic process | 0.42 | 0.77 | 0.6132732 | 1 |
| positive regulation of phosphate metabolic process | 0.42 | 0.77 | 0.6132732 | 1 |
| interleukin-2 biosynthetic process | 0.42 | 0.77 | 0.6132732 | 1 |
| axon ensheathment | 0.42 | 0.77 | 0.6132732 | 1 |
| insulin receptor signaling pathway | 0.56 | 0.77 | 0.6595536 | 1 |
| cell projection biogenesis | 0.56 | 0.77 | 0.6595536 | 1 |
| protein ubiquitination | 0.56 | 0.77 | 0.6595536 | 1 |
| positive regulation of lymphocyte proliferation | 0.56 | 0.77 | 0.6595536 | 1 |
| androgen receptor signaling pathway | 0.83 | 0.38 | 0.68225 | 1 |
| regulation of neurogenesis | 0.83 | 0.38 | 0.68225 | 1 |
| regulation of MAPK activity | 0.83 | 1.15 | 0.7065722 | 1 |
| hemopoiesis | 3.75 | 4.21 | 0.7110969 | 1 |
| positive regulation of protein biosynthetic process | 0.97 | 1.15 | 0.7309491 | 1 |
| regulation of cytokine biosynthetic process | 1.25 | 1.53 | 0.7543467 | 1 |
| glucose metabolic process | 1.67 | 1.15 | 0.7705619 | 1 |

| | | | | |
|---|---|---|---|---|
| regulation of I-kappaB kinase/NF-kappaB cascade | 1.67 | 1.15 | 0.7705619 | 1 |
| microtubule-based movement | 1.39 | 1.53 | 0.7706805 | 1 |
| regulation of Ras protein signal transduction | 2.36 | 1.92 | 0.8102265 | 1 |
| Rho protein signal transduction | 2.5 | 1.92 | 0.8115185 | 1 |
| blood vessel morphogenesis | 3.47 | 3.07 | 0.8435508 | 1 |
| regulation of transcription, DNA-dependent | 26.94 | 27.59 | 0.8709582 | 1 |
| chondroitin sulfate metabolic process | 0.14 | 0 | 1 | 1 |
| eye development (sensu Endopterygota) | 0.14 | 0 | 1 | 1 |
| lymphocyte costimulation | 0.14 | 0 | 1 | 1 |
| positive regulation of B cell activation | 0.28 | 0.38 | 1 | 1 |
| interleukin-1 biosynthetic process | 0.14 | 0 | 1 | 1 |
| regulation of DNA recombination | 0.14 | 0 | 1 | 1 |
| acyl-CoA metabolic process | 0.14 | 0 | 1 | 1 |
| neurotransmitter metabolic process | 0.69 | 0.77 | 1 | 1 |
| negative regulation of smoothened signaling pathway | 0.14 | 0 | 1 | 1 |
| somatic diversification of immunoglobulins | 0.28 | 0.38 | 1 | 1 |
| neurite regeneration | 0.14 | 0 | 1 | 1 |
| lysosomal transport | 0.28 | 0.38 | 1 | 1 |
| ammonium transport | 0.14 | 0 | 1 | 1 |
| protein depalmitoylation | 0.14 | 0 | 1 | 1 |
| purine ribonucleoside diphosphate metabolic process | 0.14 | 0 | 1 | 1 |
| negative regulation of protein biosynthetic process | 0.56 | 0.38 | 1 | 1 |
| synaptic transmission, glutamatergic | 0.28 | 0 | 1 | 1 |
| cysteine metabolic process | 0.14 | 0 | 1 | 1 |
| 'de novo' pyrimidine base biosynthetic process | 0.28 | 0 | 1 | 1 |
| cholesterol metabolic process | 0.69 | 0.77 | 1 | 1 |
| estrogen catabolic process | 0.14 | 0 | 1 | 1 |
| positive regulation of epithelial cell differentiation | 0.14 | 0 | 1 | 1 |
| purine nucleoside diphosphate biosynthetic process | 0.14 | 0 | 1 | 1 |
| regulation of leukocyte degranulation | 0.14 | 0 | 1 | 1 |
| positive regulation of T cell activation | 0.69 | 0.77 | 1 | 1 |
| regulation of DNA replication | 0.28 | 0 | 1 | 1 |
| receptor internalization | 0.14 | 0 | 1 | 1 |
| meiosis | 0.97 | 0.77 | 1 | 1 |
| mitosis | 2.08 | 1.92 | 1 | 1 |
| chondroitin sulfate proteoglycan biosynthetic process | 0.14 | 0 | 1 | 1 |

| | | | | |
|---|---|---|---|---|
| sperm chromatin decondensation | 0.14 | 0 | 1 | 1 |
| RNA interference | 0.14 | 0 | 1 | 1 |
| interleukin-8 biosynthetic process | 0.14 | 0 | 1 | 1 |
| embryonic forelimb morphogenesis | 0.14 | 0 | 1 | 1 |
| peroxisomal long-chain fatty acid import | 0.14 | 0 | 1 | 1 |
| pyrimidine deoxyribonucleoside catabolic process | 0.14 | 0 | 1 | 1 |
| fatty acid biosynthetic process | 0.83 | 0.77 | 1 | 1 |
| regulation of lymphocyte mediated immunity | 0.14 | 0 | 1 | 1 |
| positive regulation of protein modification | 0.14 | 0 | 1 | 1 |
| male genitalia development | 0.28 | 0.38 | 1 | 1 |
| regulation of cyclic nucleotide metabolic process | 0.28 | 0.38 | 1 | 1 |
| glial cell migration | 0.14 | 0 | 1 | 1 |
| epidermal growth factor receptor signaling pathway | 0.56 | 0.38 | 1 | 1 |
| mRNA transcription | 0.14 | 0 | 1 | 1 |
| embryonic ectodermal gut development | 0.28 | 0 | 1 | 1 |
| estrogen biosynthetic process | 0.14 | 0 | 1 | 1 |
| sodium ion homeostasis | 0.14 | 0 | 1 | 1 |
| macrophage chemotaxis | 0.28 | 0 | 1 | 1 |
| protein amino acid methylation | 0.28 | 0 | 1 | 1 |
| induction of an organ | 0.28 | 0.38 | 1 | 1 |
| regulation of cell killing | 0.28 | 0.38 | 1 | 1 |
| UDP-N-acetylglucosamine transport | 0.14 | 0 | 1 | 1 |
| killing of cells of another organism | 0.14 | 0 | 1 | 1 |
| purine ribonucleotide biosynthetic process | 0.83 | 0.77 | 1 | 1 |
| patched ligand processing | 0.14 | 0 | 1 | 1 |
| regulation of fatty acid metabolic process | 0.14 | 0 | 1 | 1 |
| negative regulation of lymphocyte proliferation | 0.28 | 0 | 1 | 1 |
| neuroblast proliferation | 0.14 | 0 | 1 | 1 |
| midbrain development | 0.28 | 0 | 1 | 1 |
| arginine metabolic process | 0.14 | 0 | 1 | 1 |
| interleukin-13 biosynthetic process | 0.14 | 0 | 1 | 1 |
| neuron migration | 1.67 | 1.53 | 1 | 1 |
| fatty acid oxidation | 0.28 | 0.38 | 1 | 1 |
| modification by symbiont of host cellular component | 0.14 | 0 | 1 | 1 |
| establishment of apical/basal cell polarity | 0.14 | 0 | 1 | 1 |
| beta-tubulin folding | 0.14 | 0 | 1 | 1 |

| | | | | |
|---|---|---|---|---|
| telomere maintenance | 0.14 | 0 | 1 | 1 |
| hydrogen ion homeostasis | 0.28 | 0 | 1 | 1 |
| positive regulation of endocytosis | 0.28 | 0.38 | 1 | 1 |
| chaperone cofactor-dependent protein folding | 0.14 | 0 | 1 | 1 |
| purine nucleoside triphosphate biosynthetic process | 0.69 | 0.77 | 1 | 1 |
| valine metabolic process | 0.14 | 0 | 1 | 1 |
| transcription-coupled nucleotide-excision repair | 0.14 | 0 | 1 | 1 |
| peptidyl-threonine modification | 0.14 | 0 | 1 | 1 |
| UDP-xylose transport | 0.14 | 0 | 1 | 1 |
| chondrocyte differentiation | 0.14 | 0 | 1 | 1 |
| succinyl-CoA metabolic process | 0.28 | 0.38 | 1 | 1 |
| amyloid precursor protein metabolic process | 0.14 | 0 | 1 | 1 |
| histidine family amino acid catabolic process | 0.28 | 0.38 | 1 | 1 |
| tripartite regional subdivision | 0.14 | 0 | 1 | 1 |
| oocyte development | 0.28 | 0 | 1 | 1 |
| regulation of gamma-delta T cell activation | 0.14 | 0 | 1 | 1 |
| pyrimidine ribonucleotide biosynthetic process | 0.28 | 0.38 | 1 | 1 |
| interleukin-12 biosynthetic process | 0.28 | 0.38 | 1 | 1 |
| N-acetylglucosamine metabolic process | 0.28 | 0 | 1 | 1 |
| ribonucleoside diphosphate biosynthetic process | 0.14 | 0 | 1 | 1 |
| phospholipid biosynthetic process | 0.69 | 0.77 | 1 | 1 |
| L-amino acid transport | 0.14 | 0 | 1 | 1 |
| negative regulation of smooth muscle cell differentiation | 0.14 | 0 | 1 | 1 |
| tricarboxylic acid cycle | 0.28 | 0 | 1 | 1 |
| negative regulation of nucleotide metabolic process | 0.14 | 0 | 1 | 1 |
| regulation of B cell proliferation | 0.28 | 0.38 | 1 | 1 |
| negative regulation of amino acid metabolic process | 0.14 | 0 | 1 | 1 |
| negative regulation of hormone secretion | 0.14 | 0 | 1 | 1 |
| neural crest cell migration | 0.28 | 0 | 1 | 1 |
| establishment of lymphocyte polarity | 0.14 | 0 | 1 | 1 |
| interleukin-1 beta secretion | 0.28 | 0 | 1 | 1 |
| ribonucleoside triphosphate biosynthetic process | 0.69 | 0.77 | 1 | 1 |
| regulation of translational initiation | 0.28 | 0.38 | 1 | 1 |
| mRNA transport | 0.56 | 0.38 | 1 | 1 |
| protein sumoylation | 0.28 | 0 | 1 | 1 |
| mitochondrial DNA replication | 0.14 | 0 | 1 | 1 |

| | | | | |
|---|---|---|---|---|
| spindle checkpoint | 0.14 | 0 | 1 | 1 |
| hindbrain development | 0.28 | 0 | 1 | 1 |
| positive regulation of progression through mitotic cell cyc | 0.14 | 0 | 1 | 1 |
| choline metabolic process | 0.14 | 0 | 1 | 1 |
| embryonic digestive tract morphogenesis | 0.28 | 0.38 | 1 | 1 |
| purine ribonucleoside triphosphate metabolic process | 0.69 | 0.77 | 1 | 1 |
| long-chain fatty acid metabolic process | 0.14 | 0 | 1 | 1 |
| gut morphogenesis | 0.28 | 0.38 | 1 | 1 |
| uracil catabolic process | 0.14 | 0 | 1 | 1 |
| protein amino acid acetylation | 0.14 | 0 | 1 | 1 |
| chaperonin-mediated tubulin folding | 0.14 | 0 | 1 | 1 |
| positive regulation of DNA metabolic process | 0.28 | 0.38 | 1 | 1 |
| regulation of phosphorylation | 0.97 | 0.77 | 1 | 1 |
| bile acid transport | 0.14 | 0 | 1 | 1 |
| chemokine biosynthetic process | 0.14 | 0 | 1 | 1 |
| negative regulation of phosphate metabolic process | 0.14 | 0 | 1 | 1 |
| endoderm formation | 0.14 | 0 | 1 | 1 |
| vesicle targeting | 0.28 | 0.38 | 1 | 1 |
| negative regulation of small GTPase mediated signal tra | 0.14 | 0 | 1 | 1 |
| pyrimidine ribonucleoside triphosphate metabolic proces | 0.28 | 0.38 | 1 | 1 |
| peptidyl-asparagine modification | 0.42 | 0.38 | 1 | 1 |
| hyaluronan metabolic process | 0.14 | 0 | 1 | 1 |
| tyrosine metabolic process | 0.14 | 0 | 1 | 1 |
| neutral amino acid transport | 0.14 | 0 | 1 | 1 |
| microtubule bundle formation | 0.28 | 0 | 1 | 1 |
| signal peptide processing | 0.28 | 0 | 1 | 1 |
| somatic diversification of immune receptors via germline | 0.28 | 0 | 1 | 1 |
| nerve growth factor receptor signaling pathway | 0.14 | 0 | 1 | 1 |
| leukocyte mediated cytotoxicity | 0.28 | 0.38 | 1 | 1 |
| retrograde transport, endosome to Golgi | 0.14 | 0 | 1 | 1 |
| regulation of mitochondrial membrane permeability | 0.14 | 0 | 1 | 1 |
| odontogenesis (sensu Vertebrata) | 0.42 | 0.38 | 1 | 1 |
| transition metal ion transport | 0.56 | 0.38 | 1 | 1 |
| regulation of phosphoinositide 3-kinase activity | 0.14 | 0 | 1 | 1 |
| cartilage condensation | 0.28 | 0 | 1 | 1 |
| regulation of alpha-beta T cell activation | 0.14 | 0 | 1 | 1 |

| | | | | |
|---|---|---|---|---|
| thymidine metabolic process | 0.14 | 0 | 1 | 1 |
| peptide hormone processing | 0.14 | 0 | 1 | 1 |
| blood vessel maturation | 0.14 | 0 | 1 | 1 |
| spermatid development | 0.14 | 0 | 1 | 1 |
| fibroblast growth factor receptor signaling pathway | 0.28 | 0.38 | 1 | 1 |
| phagocytosis, engulfment | 0.14 | 0 | 1 | 1 |
| signal complex formation | 0.28 | 0 | 1 | 1 |
| phosphate ion homeostasis | 0.14 | 0 | 1 | 1 |
| tumor necrosis factor-beta biosynthetic process | 0.28 | 0 | 1 | 1 |
| peptidyl-histidine modification | 0.14 | 0 | 1 | 1 |
| primary follicle stage, oogenesis (sensu Mammalia) | 0.14 | 0 | 1 | 1 |
| regulation of nucleotide biosynthetic process | 0.14 | 0 | 1 | 1 |
| regulation of MAPKKK cascade | 0.28 | 0 | 1 | 1 |
| neurotransmitter secretion | 1.53 | 1.53 | 1 | 1 |
| positive regulation of exocytosis | 0.28 | 0 | 1 | 1 |
| estrogen receptor signaling pathway | 0.14 | 0 | 1 | 1 |
| regulation of MHC class II biosynthetic process | 0.28 | 0 | 1 | 1 |
| RNA polyadenylation | 0.28 | 0 | 1 | 1 |
| positive regulation of immune response to tumor cell | 0.14 | 0 | 1 | 1 |
| regulation of pinocytosis | 0.28 | 0 | 1 | 1 |
| actin cytoskeleton reorganization | 0.14 | 0 | 1 | 1 |
| Notch receptor processing | 0.14 | 0 | 1 | 1 |
| ephrin receptor signaling pathway | 0.14 | 0 | 1 | 1 |
| mannose metabolic process | 0.28 | 0 | 1 | 1 |
| malate metabolic process | 0.28 | 0 | 1 | 1 |
| positive regulation of leukocyte mediated immunity | 0.14 | 0 | 1 | 1 |
| lymph node development | 0.14 | 0 | 1 | 1 |
| rostrocaudal neural tube patterning | 0.14 | 0 | 1 | 1 |
| negative regulation of blood coagulation | 0.28 | 0 | 1 | 1 |
| cGMP catabolic process | 0.14 | 0 | 1 | 1 |
| lymphocyte chemotaxis | 0.28 | 0 | 1 | 1 |
| L-serine metabolic process | 0.28 | 0 | 1 | 1 |
| succinate transport | 0.14 | 0 | 1 | 1 |
| regulation of gonadotropin secretion | 0.14 | 0 | 1 | 1 |
| actin polymerization and/or depolymerization | 1.11 | 1.15 | 1 | 1 |
| alpha-beta T cell proliferation | 0.28 | 0 | 1 | 1 |

| | | | | |
|---|---|---|---|---|
| positive regulation of amino acid metabolic process | 0.42 | 0.38 | 1 | 1 |
| alveolus development | 0.14 | 0 | 1 | 1 |
| lipid raft polarization | 0.14 | 0 | 1 | 1 |
| branched chain family amino acid catabolic process | 0.14 | 0 | 1 | 1 |
| microtubule polymerization or depolymerization | 0.14 | 0 | 1 | 1 |
| base conversion or substitution editing | 0.14 | 0 | 1 | 1 |
| base-excision repair, gap-filling | 0.14 | 0 | 1 | 1 |
| G2 phase of mitotic cell cycle | 0.28 | 0 | 1 | 1 |
| eye morphogenesis | 0.42 | 0.38 | 1 | 1 |
| proton transport | 0.69 | 0.38 | 1 | 1 |
| regulation of T cell proliferation | 0.83 | 0.77 | 1 | 1 |
| posterior midgut development | 0.14 | 0 | 1 | 1 |
| glucose transport | 0.42 | 0.38 | 1 | 1 |
| B cell mediated immunity | 0.69 | 0.38 | 1 | 1 |
| intermediate mesoderm development | 0.14 | 0 | 1 | 1 |
| negative regulation of cytokine and chemokine mediatec | 0.14 | 0 | 1 | 1 |
| thiamin diphosphate biosynthetic process | 0.14 | 0 | 1 | 1 |
| glucan biosynthetic process | 0.14 | 0 | 1 | 1 |
| citrate transport | 0.14 | 0 | 1 | 1 |
| RNA splicing, via transesterification reactions | 0.69 | 0.77 | 1 | 1 |
| regulation of catecholamine metabolic process | 0.14 | 0 | 1 | 1 |
| JNK cascade | 0.83 | 0.77 | 1 | 1 |
| regulation of calcium-mediated signaling | 0.14 | 0 | 1 | 1 |
| glycosphingolipid metabolic process | 0.42 | 0.38 | 1 | 1 |
| fructose metabolic process | 0.14 | 0 | 1 | 1 |
| follicle-stimulating hormone secretion | 0.14 | 0 | 1 | 1 |
| mRNA editing | 0.14 | 0 | 1 | 1 |
| histidine metabolic process | 0.28 | 0.38 | 1 | 1 |
| embryonic skeletal morphogenesis | 0.14 | 0 | 1 | 1 |
| G2/M transition of mitotic cell cycle | 0.14 | 0 | 1 | 1 |
| negative regulation of Wnt receptor signaling pathway | 0.28 | 0 | 1 | 1 |
| indolalkylamine biosynthetic process | 0.14 | 0 | 1 | 1 |
| establishment and/or maintenance of epithelial cell polar | 0.14 | 0 | 1 | 1 |
| interleukin-4 biosynthetic process | 0.14 | 0 | 1 | 1 |
| modification by organism of cell membrane in other orga | 0.14 | 0 | 1 | 1 |
| embryonic hindlimb morphogenesis | 0.28 | 0 | 1 | 1 |

| | | | | |
|---|---|---|---|---|
| pyridoxine biosynthetic process | 0.14 | 0 | 1 | 1 |
| granulocyte macrophage colony-stimulating factor biosyı | 0.28 | 0 | 1 | 1 |
| forebrain development | 1.11 | 1.15 | 1 | 1 |
| purine ribonucleoside salvage | 0.28 | 0 | 1 | 1 |
| ubiquinone biosynthetic process | 0.14 | 0 | 1 | 1 |
| positive regulation of hormone secretion | 0.14 | 0 | 1 | 1 |
| T cell mediated immunity | 0.14 | 0 | 1 | 1 |
| vascular endothelial growth factor receptor signaling pat | 0.14 | 0 | 1 | 1 |
| regulation of actin filament length | 0.69 | 0.77 | 1 | 1 |
| negative regulation of DNA metabolic process | 0.14 | 0 | 1 | 1 |
| entry into host cell | 0.28 | 0 | 1 | 1 |
| opsonization | 0.14 | 0 | 1 | 1 |
| coenzyme A biosynthetic process | 0.14 | 0 | 1 | 1 |
| glycolipid biosynthetic process | 0.28 | 0.38 | 1 | 1 |
| protein heterotetramerization | 0.14 | 0 | 1 | 1 |
| sphingolipid biosynthetic process | 0.28 | 0 | 1 | 1 |
| arm morphogenesis | 0.14 | 0 | 1 | 1 |
| mRNA 3'-end processing | 0.28 | 0 | 1 | 1 |
| regulation of nucleocytoplasmic transport | 0.28 | 0 | 1 | 1 |
| G1/S transition of mitotic cell cycle | 0.56 | 0.38 | 1 | 1 |
| fused antrum stage, oogenesis (sensu Mammalia) | 0.14 | 0 | 1 | 1 |
| I-kappaB phosphorylation | 0.28 | 0 | 1 | 1 |
| neural tube formation | 0.42 | 0.38 | 1 | 1 |
| eosinophil chemotaxis | 0.14 | 0 | 1 | 1 |
| regulation of interleukin-1 secretion | 0.28 | 0 | 1 | 1 |
| male germ-line stem cell division | 0.14 | 0 | 1 | 1 |
| neutrophil chemotaxis | 0.56 | 0.38 | 1 | 1 |
| C21-steroid hormone biosynthetic process | 0.14 | 0 | 1 | 1 |
| tryptophan metabolic process | 0.14 | 0 | 1 | 1 |
| interleukin-3 biosynthetic process | 0.14 | 0 | 1 | 1 |
| cytoplasmic sequestering of protein | 0.14 | 0 | 1 | 1 |
| positive regulation of innate immune response | 0.14 | 0 | 1 | 1 |

| Gene Ontology : biological process. Level: 9 | Percentage of genes with allele-specific expression | Percentage of genes without allele-specific expression | Unadjusted pvalue | Adjusted pvalueFDR |
|---|---|---|---|---|
| regulation of ossification | 0 | 2 | 0.01934914 | 1 |
| bone mineralization | 0 | 2 | 0.01934914 | 1 |
| positive regulation of transcription, DNA-dependent | 8.87 | 3.33 | 0.02764696 | 1 |
| positive regulation of programmed cell death | 6.9 | 2.67 | 0.0648839 | 1 |
| phototransduction | 0 | 1.33 | 0.07242854 | 1 |
| calcium-dependent phospholipase A2 activation | 0 | 1.33 | 0.07242854 | 1 |
| blood vessel remodeling | 0 | 1.33 | 0.07242854 | 1 |
| chromatin assembly | 0.74 | 2.67 | 0.08893205 | 1 |
| collagen catabolic process | 0.49 | 2 | 0.1245022 | 1 |
| regulation of apoptosis | 13.55 | 8.67 | 0.1445579 | 1 |
| regulation of transcription from RNA polymerase II pron | 14.29 | 9.33 | 0.1541752 | 1 |
| modification-dependent protein catabolic process | 3.69 | 6.67 | 0.1647821 | 1 |
| skeletal muscle fiber development | 1.48 | 3.33 | 0.1770124 | 1 |
| neuron fate commitment | 0.25 | 1.33 | 0.1785873 | 1 |
| regulation of neuronal synaptic plasticity | 0.25 | 1.33 | 0.1785873 | 1 |
| protein amino acid O-linked glycosylation | 0.25 | 1.33 | 0.1785873 | 1 |
| synaptic vesicle exocytosis | 0.25 | 1.33 | 0.1785873 | 1 |
| protein export from nucleus | 0.25 | 1.33 | 0.1785873 | 1 |
| phosphoinositide metabolic process | 0.25 | 1.33 | 0.1785873 | 1 |
| hair follicle development | 1.72 | 0 | 0.1980866 | 1 |
| regulation of mitosis | 1.48 | 0 | 0.1981695 | 1 |
| triacylglycerol biosynthetic process | 0 | 0.67 | 0.2697842 | 1 |
| striated muscle cell development | 0 | 0.67 | 0.2697842 | 1 |
| regulation of transcription from RNA polymerase I prom | 0 | 0.67 | 0.2697842 | 1 |
| S-adenosylmethionine biosynthetic process | 0 | 0.67 | 0.2697842 | 1 |
| neurotransmitter catabolic process | 0 | 0.67 | 0.2697842 | 1 |
| mesoderm formation | 0 | 0.67 | 0.2697842 | 1 |
| sphingoid catabolic process | 0 | 0.67 | 0.2697842 | 1 |
| Schwann cell differentiation | 0 | 0.67 | 0.2697842 | 1 |
| positive regulation of B cell proliferation | 0 | 0.67 | 0.2697842 | 1 |
| protein targeting to peroxisome | 0 | 0.67 | 0.2697842 | 1 |
| telencephalon development | 0 | 0.67 | 0.2697842 | 1 |
| positive regulation of DNA ligation | 0 | 0.67 | 0.2697842 | 1 |
| endochondral ossification | 0 | 0.67 | 0.2697842 | 1 |
| protein autoubiquitination | 0 | 0.67 | 0.2697842 | 1 |

| | | | | |
|---|---|---|---|---|
| retrograde transport, vesicle recycling within Golgi | 0 | 0.67 | 0.2697842 | 1 |
| platelet activating factor biosynthetic process | 0 | 0.67 | 0.2697842 | 1 |
| positive regulation of gliogenesis | 0 | 0.67 | 0.2697842 | 1 |
| cholesterol catabolic process | 0 | 0.67 | 0.2697842 | 1 |
| ovulation (sensu Mammalia) | 0 | 0.67 | 0.2697842 | 1 |
| asparagine biosynthetic process | 0 | 0.67 | 0.2697842 | 1 |
| oxidized low-density lipoprotein catabolic process | 0 | 0.67 | 0.2697842 | 1 |
| segment polarity determination | 0 | 0.67 | 0.2697842 | 1 |
| mitotic centrosome separation | 0 | 0.67 | 0.2697842 | 1 |
| negative regulation of transcription factor activity | 0 | 0.67 | 0.2697842 | 1 |
| galactolipid catabolic process | 0 | 0.67 | 0.2697842 | 1 |
| glycogen catabolic process | 0 | 0.67 | 0.2697842 | 1 |
| fatty acid beta-oxidation | 0 | 0.67 | 0.2697842 | 1 |
| glutamine biosynthetic process | 0 | 0.67 | 0.2697842 | 1 |
| regulation of cGMP metabolic process | 0 | 0.67 | 0.2697842 | 1 |
| lagging strand elongation | 0 | 0.67 | 0.2697842 | 1 |
| Golgi to plasma membrane transport | 0 | 0.67 | 0.2697842 | 1 |
| leukotriene metabolic process | 0.49 | 1.33 | 0.2952085 | 1 |
| protein prenylation | 0.49 | 1.33 | 0.2952085 | 1 |
| eye development (sensu Mammalia) | 0.49 | 1.33 | 0.2952085 | 1 |
| ureteric bud branching | 0.49 | 1.33 | 0.2952085 | 1 |
| neurotransmitter biosynthetic process | 0.49 | 1.33 | 0.2952085 | 1 |
| actin filament depolymerization | 0.49 | 1.33 | 0.2952085 | 1 |
| positive regulation of neurogenesis | 1.23 | 0 | 0.3307322 | 1 |
| activation of protein kinase activity | 1.97 | 3.33 | 0.3510684 | 1 |
| G-protein signaling, coupled to cAMP nucleotide secon | 3.2 | 1.33 | 0.3755438 | 1 |
| inner ear morphogenesis | 0.99 | 2 | 0.3940697 | 1 |
| protein amino acid autophosphorylation | 0.99 | 2 | 0.3940697 | 1 |
| neuron development | 9.36 | 6.67 | 0.3955965 | 1 |
| negative regulation of programmed cell death | 6.4 | 4 | 0.4111837 | 1 |
| cytosolic calcium ion homeostasis | 1.23 | 2 | 0.4503502 | 1 |
| regulation of activated T cell proliferation | 0.25 | 0.67 | 0.4671398 | 1 |
| astrocyte differentiation | 0.25 | 0.67 | 0.4671398 | 1 |
| L-phenylalanine catabolic process | 0.25 | 0.67 | 0.4671398 | 1 |
| mast cell degranulation | 0.25 | 0.67 | 0.4671398 | 1 |
| norepinephrine metabolic process | 0.25 | 0.67 | 0.4671398 | 1 |

| | | | | |
|---|---|---|---|---|
| regulation of S phase of mitotic cell cycle | 0.25 | 0.67 | 0.4671398 | 1 |
| protein targeting to lysosome | 0.25 | 0.67 | 0.4671398 | 1 |
| regulation of ARF protein signal transduction | 0.25 | 0.67 | 0.4671398 | 1 |
| phospholipase C activation | 0.25 | 0.67 | 0.4671398 | 1 |
| substrate-bound cell migration, cell extension | 0.25 | 0.67 | 0.4671398 | 1 |
| positive regulation of JAK-STAT cascade | 0.25 | 0.67 | 0.4671398 | 1 |
| glutamate transport | 0.25 | 0.67 | 0.4671398 | 1 |
| regulation of glial cell differentiation | 0.25 | 0.67 | 0.4671398 | 1 |
| apocarotenoid metabolic process | 0.25 | 0.67 | 0.4671398 | 1 |
| negative regulation of neurogenesis | 0.25 | 0.67 | 0.4671398 | 1 |
| positive regulation of receptor mediated endocytosis | 0.25 | 0.67 | 0.4671398 | 1 |
| negative regulation of cyclin-dependent protein kinase a | 0.25 | 0.67 | 0.4671398 | 1 |
| tRNA splicing | 0.25 | 0.67 | 0.4671398 | 1 |
| regulation of interferon-gamma biosynthetic process | 0.25 | 0.67 | 0.4671398 | 1 |
| regulation of BMP signaling pathway | 0.25 | 0.67 | 0.4671398 | 1 |
| regulation of calcium ion transport | 0.25 | 0.67 | 0.4671398 | 1 |
| regulation of mRNA stability | 0.25 | 0.67 | 0.4671398 | 1 |
| diacylglycerol biosynthetic process | 0.25 | 0.67 | 0.4671398 | 1 |
| glycosphingolipid catabolic process | 0.25 | 0.67 | 0.4671398 | 1 |
| axon cargo transport | 0.25 | 0.67 | 0.4671398 | 1 |
| angiogenesis | 4.93 | 3.33 | 0.4971736 | 1 |
| peptidyl-tyrosine phosphorylation | 1.72 | 2.67 | 0.4983115 | 1 |
| L-fucose metabolic process | 0.74 | 0 | 0.5671713 | 1 |
| positive regulation of positive chemotaxis | 0.74 | 0 | 0.5671713 | 1 |
| mismatch repair | 0.74 | 0 | 0.5671713 | 1 |
| tRNA aminoacylation for protein translation | 0.74 | 0 | 0.5671713 | 1 |
| energy coupled proton transport, down electrochemical | 0.74 | 0 | 0.5671713 | 1 |
| actin filament bundle formation | 0.74 | 0 | 0.5671713 | 1 |
| immune response-activating signal transduction | 0.99 | 0 | 0.5783899 | 1 |
| DNA damage checkpoint | 0.99 | 0 | 0.5783899 | 1 |
| transcription initiation from RNA polymerase II promote | 0.99 | 0 | 0.5783899 | 1 |
| negative regulation of cell growth | 0.99 | 0 | 0.5783899 | 1 |
| regulation of interleukin-6 biosynthetic process | 0.99 | 0 | 0.5783899 | 1 |
| chromatin remodeling | 0.99 | 0 | 0.5783899 | 1 |
| protein import into nucleus | 2.96 | 4 | 0.5901606 | 1 |
| meiosis I | 0.74 | 1.33 | 0.6152426 | 1 |

| | | | | |
|---|---|---|---|---|
| regulation of interleukin-2 biosynthetic process | 0.74 | 1.33 | 0.6152426 | 1 |
| ceramide metabolic process | 0.74 | 1.33 | 0.6152426 | 1 |
| positive regulation of MAPK activity | 0.74 | 1.33 | 0.6152426 | 1 |
| positive regulation of phosphorylation | 0.74 | 1.33 | 0.6152426 | 1 |
| myelination | 0.74 | 1.33 | 0.6152426 | 1 |
| dopamine metabolic process | 0.74 | 1.33 | 0.6152426 | 1 |
| actin filament polymerization | 0.99 | 1.33 | 0.6631452 | 1 |
| positive regulation of T cell proliferation | 0.99 | 1.33 | 0.6631452 | 1 |
| regulation of actin polymerization and/or depolymerizati | 0.99 | 1.33 | 0.6631452 | 1 |
| epidermis morphogenesis | 1.72 | 0.67 | 0.6889394 | 1 |
| protein amino acid N-linked glycosylation | 1.72 | 0.67 | 0.6889394 | 1 |
| glucose catabolic process | 1.72 | 0.67 | 0.6889394 | 1 |
| positive regulation of cytokine biosynthetic process | 1.48 | 2 | 0.7080766 | 1 |
| positive regulation of I-kappaB kinase/NF-kappaB casc | 2.71 | 2 | 0.7684468 | 1 |
| regulation of Rho protein signal transduction | 3.45 | 2.67 | 0.7910422 | 1 |
| leukocyte differentiation | 4.19 | 4.67 | 0.8155387 | 1 |
| negative regulation of transcription, DNA-dependent | 6.9 | 6 | 0.8485424 | 1 |
| neutrophil apoptosis | 0.25 | 0 | 1 | 1 |
| regulation of granulocyte macrophage colony-stimulatin | 0.49 | 0 | 1 | 1 |
| regulation of microtubule polymerization or depolymeriz | 0.25 | 0 | 1 | 1 |
| positive regulation of translational initiation | 0.25 | 0 | 1 | 1 |
| ectodermal gut morphogenesis | 0.49 | 0 | 1 | 1 |
| peptidyl-tyrosine sulfation | 0.25 | 0 | 1 | 1 |
| RNA export from nucleus | 0.25 | 0 | 1 | 1 |
| endoplasmic reticulum calcium ion homeostasis | 0.25 | 0 | 1 | 1 |
| primary neural tube formation | 0.49 | 0.67 | 1 | 1 |
| Cdc42 protein signal transduction | 0.25 | 0 | 1 | 1 |
| catecholamine uptake during transmission of nerve imp | 0.25 | 0 | 1 | 1 |
| NADP metabolic process | 0.49 | 0 | 1 | 1 |
| amyloid precursor protein catabolic process | 0.25 | 0 | 1 | 1 |
| entry of virus into host cell | 0.49 | 0 | 1 | 1 |
| copper ion homeostasis | 0.49 | 0 | 1 | 1 |
| regulation of interleukin-13 biosynthetic process | 0.25 | 0 | 1 | 1 |
| glucose import | 0.25 | 0 | 1 | 1 |
| GTP metabolic process | 0.49 | 0.67 | 1 | 1 |
| thymidine catabolic process | 0.25 | 0 | 1 | 1 |

| | | | | |
|---|---|---|---|---|
| transcription initiation from RNA polymerase III promote | 0.25 | 0 | 1 | 1 |
| zinc ion transport | 0.25 | 0 | 1 | 1 |
| energy coupled proton transport, against electrochemic | 0.49 | 0 | 1 | 1 |
| regulation of transforming growth factor beta receptor s | 0.49 | 0 | 1 | 1 |
| regulation of JNK activity | 0.25 | 0 | 1 | 1 |
| glutamate secretion | 0.25 | 0 | 1 | 1 |
| mitotic spindle organization and biogenesis | 0.25 | 0 | 1 | 1 |
| skin development | 0.25 | 0 | 1 | 1 |
| vasculogenesis | 0.74 | 0.67 | 1 | 1 |
| misfolded or incompletely synthesized protein catabolic | 0.25 | 0 | 1 | 1 |
| regulation of leukocyte mediated cytotoxicity | 0.49 | 0.67 | 1 | 1 |
| L-serine biosynthetic process | 0.49 | 0 | 1 | 1 |
| regulation of protein amino acid phosphorylation | 1.23 | 1.33 | 1 | 1 |
| gastrulation (sensu Mammalia) | 0.49 | 0 | 1 | 1 |
| microtubule polymerization | 0.25 | 0 | 1 | 1 |
| spermatid nuclear differentiation | 0.25 | 0 | 1 | 1 |
| cytolysis of cells of another organism | 0.25 | 0 | 1 | 1 |
| lamellipodium biogenesis | 0.49 | 0 | 1 | 1 |
| mitotic anaphase | 0.25 | 0 | 1 | 1 |
| positive regulation of lymphocyte mediated immunity | 0.25 | 0 | 1 | 1 |
| killing of cells in other organism during symbiotic intera | 0.25 | 0 | 1 | 1 |
| positive regulation of pinocytosis | 0.49 | 0 | 1 | 1 |
| microtubule depolymerization | 0.25 | 0 | 1 | 1 |
| fucose biosynthetic process | 0.25 | 0 | 1 | 1 |
| natural killer cell mediated immune response to tumor c | 0.25 | 0 | 1 | 1 |
| regulation of chromatin assembly or disassembly | 0.25 | 0 | 1 | 1 |
| regulation of cyclic nucleotide biosynthetic process | 0.25 | 0 | 1 | 1 |
| activated T cell apoptosis | 0.25 | 0 | 1 | 1 |
| regulation of Ras GTPase activity | 0.25 | 0 | 1 | 1 |
| chondroitin sulfate biosynthetic process | 0.25 | 0 | 1 | 1 |
| regulation of interleukin-3 biosynthetic process | 0.25 | 0 | 1 | 1 |
| phosphatidylcholine metabolic process | 0.25 | 0 | 1 | 1 |
| regulation of interleukin-1 beta secretion | 0.49 | 0 | 1 | 1 |
| positive regulation of alpha-beta T cell activation | 0.25 | 0 | 1 | 1 |
| negative regulation of striated muscle development | 0.49 | 0 | 1 | 1 |
| positive regulation of nucleocytoplasmic transport | 0.25 | 0 | 1 | 1 |

| | | | | |
|---|---|---|---|---|
| regulation of neuron differentiation | 1.72 | 1.33 | 1 | 1 |
| positive regulation of gamma-delta T cell activation | 0.25 | 0 | 1 | 1 |
| covalent chromatin modification | 1.23 | 0.67 | 1 | 1 |
| positive regulation of MHC class II biosynthetic process | 0.25 | 0 | 1 | 1 |
| apoptotic program | 1.97 | 1.33 | 1 | 1 |
| negative regulation of cytokine biosynthetic process | 0.49 | 0.67 | 1 | 1 |
| protein targeting to vacuole | 0.25 | 0 | 1 | 1 |
| regulation of natural killer cell mediated immunity | 0.25 | 0 | 1 | 1 |
| prostanoid metabolic process | 0.49 | 0 | 1 | 1 |
| insulin processing | 0.25 | 0 | 1 | 1 |
| embryonic eye morphogenesis | 0.25 | 0 | 1 | 1 |
| fucose catabolic process | 0.49 | 0 | 1 | 1 |
| regulation of epidermal growth factor receptor activity | 0.49 | 0 | 1 | 1 |
| neuron apoptosis | 0.49 | 0 | 1 | 1 |
| negative regulation of gonadotropin secretion | 0.25 | 0 | 1 | 1 |
| glycerophospholipid biosynthetic process | 0.49 | 0.67 | 1 | 1 |
| T cell costimulation | 0.25 | 0 | 1 | 1 |
| regulation of synaptic transmission, glutamatergic | 0.25 | 0 | 1 | 1 |
| determination of anterior/posterior axis, embryo | 0.25 | 0 | 1 | 1 |
| positive regulation of interleukin-1 secretion | 0.49 | 0 | 1 | 1 |
| neuroblast proliferation (sensu Vertebrata) | 0.25 | 0 | 1 | 1 |
| GDP-mannose metabolic process | 0.25 | 0 | 1 | 1 |
| oocyte maturation | 0.49 | 0 | 1 | 1 |
| protein palmitoylation | 0.25 | 0 | 1 | 1 |
| regulation of interleukin-8 biosynthetic process | 0.25 | 0 | 1 | 1 |
| protein targeting to membrane | 0.25 | 0 | 1 | 1 |
| triacylglycerol mobilization | 0.25 | 0 | 1 | 1 |
| positive regulation of Ras protein signal transduction | 0.25 | 0 | 1 | 1 |
| serotonin secretion | 0.25 | 0 | 1 | 1 |
| spindle assembly | 0.25 | 0 | 1 | 1 |
| arginine biosynthetic process | 0.25 | 0 | 1 | 1 |
| chromatin disassembly | 0.25 | 0 | 1 | 1 |
| endosome to lysosome transport | 0.25 | 0 | 1 | 1 |
| regulation of chemokine biosynthetic process | 0.25 | 0 | 1 | 1 |
| positive regulation of gonadotropin secretion | 0.25 | 0 | 1 | 1 |
| iron ion homeostasis | 0.49 | 0 | 1 | 1 |

| | | | | |
|---|---|---|---|---|
| negative regulation of cyclic nucleotide metabolic proce | 0.25 | 0 | 1 | 1 |
| inflammatory cell apoptosis | 0.49 | 0 | 1 | 1 |
| gluconeogenesis | 0.49 | 0 | 1 | 1 |
| embryonic gut morphogenesis | 0.49 | 0.67 | 1 | 1 |
| catecholamine secretion | 0.25 | 0 | 1 | 1 |
| sequestering of calcium ion | 0.25 | 0 | 1 | 1 |
| somatic recombination of immunoglobulin gene segmer | 0.49 | 0 | 1 | 1 |
| purine ribonucleoside triphosphate biosynthetic process | 1.23 | 1.33 | 1 | 1 |
| positive regulation of DNA replication | 0.25 | 0 | 1 | 1 |
| regulation of neurotransmitter secretion | 0.74 | 0.67 | 1 | 1 |
| tyrosine catabolic process | 0.25 | 0 | 1 | 1 |
| negative regulation of I-kappaB kinase/NF-kappaB casc | 0.25 | 0 | 1 | 1 |
| cysteine biosynthetic process | 0.25 | 0 | 1 | 1 |
| regulation of cellular pH | 0.25 | 0 | 1 | 1 |
| regulation of follicle-stimulating hormone secretion | 0.25 | 0 | 1 | 1 |
| negative regulation of gliogenesis | 0.25 | 0 | 1 | 1 |
| modification by symbiont of host cell membrane | 0.25 | 0 | 1 | 1 |
| acetylcholine metabolic process | 0.25 | 0 | 1 | 1 |
| mRNA polyadenylation | 0.49 | 0 | 1 | 1 |
| succinyl-CoA pathway | 0.25 | 0 | 1 | 1 |
| immunoglobulin mediated immune response | 1.23 | 0.67 | 1 | 1 |
| icosanoid biosynthetic process | 0.74 | 0.67 | 1 | 1 |
| positive regulation of calcium-mediated signaling | 0.25 | 0 | 1 | 1 |
| negative regulation of MHC class II biosynthetic proces | 0.25 | 0 | 1 | 1 |
| ovarian follicle development | 0.49 | 0 | 1 | 1 |
| regulation of JNK cascade | 0.49 | 0 | 1 | 1 |
| CTP metabolic process | 0.49 | 0.67 | 1 | 1 |
| negative regulation of B cell proliferation | 0.49 | 0 | 1 | 1 |
| protein targeting to mitochondrion | 0.25 | 0 | 1 | 1 |
| transmembrane receptor protein tyrosine kinase activat | 0.25 | 0 | 1 | 1 |
| NAD metabolic process | 0.49 | 0 | 1 | 1 |
| negative regulation of T cell proliferation | 0.49 | 0 | 1 | 1 |
| mitotic sister chromatid segregation | 0.49 | 0.67 | 1 | 1 |
| fibrinolysis | 0.49 | 0 | 1 | 1 |
| hemocyte differentiation (sensu Arthropoda) | 0.25 | 0 | 1 | 1 |
| negative regulation of DNA replication | 0.25 | 0 | 1 | 1 |

| | | | | |
|---|---|---|---|---|
| regulation of odontogenesis (sensu Vertebrata) | 0.25 | 0 | 1 | 1 |
| negative regulation of nucleocytoplasmic transport | 0.49 | 0 | 1 | 1 |
| positive regulation of phosphoinositide 3-kinase activity | 0.25 | 0 | 1 | 1 |
| positive regulation of striated muscle development | 0.25 | 0 | 1 | 1 |
| negative regulation of phosphorylation | 0.25 | 0 | 1 | 1 |
| negative regulation of MAPK activity | 0.74 | 0.67 | 1 | 1 |
| positive regulation of transcription factor activity | 0.49 | 0.67 | 1 | 1 |
| glycosphingolipid biosynthetic process | 0.49 | 0 | 1 | 1 |
| regulation of cAMP metabolic process | 0.49 | 0 | 1 | 1 |
| regulation of fatty acid oxidation | 0.25 | 0 | 1 | 1 |
| peptidyl-threonine phosphorylation | 0.25 | 0 | 1 | 1 |
| iron ion transport | 0.74 | 0.67 | 1 | 1 |
| copper ion transport | 0.25 | 0 | 1 | 1 |
| myeloid cell differentiation | 3.2 | 3.33 | 1 | 1 |
| regulation of interleukin-12 biosynthetic process | 0.49 | 0.67 | 1 | 1 |
| eye morphogenesis (sensu Vertebrata) | 0.25 | 0 | 1 | 1 |
| embryonic arm morphogenesis | 0.25 | 0 | 1 | 1 |
| Golgi to endosome transport | 0.25 | 0 | 1 | 1 |
| positive regulation of leukocyte degranulation | 0.25 | 0 | 1 | 1 |
| negative regulation of nucleotide biosynthetic process | 0.25 | 0 | 1 | 1 |
| male meiosis | 0.25 | 0 | 1 | 1 |
| peptidyl-diphthamide metabolic process | 0.25 | 0 | 1 | 1 |
| ATP metabolic process | 0.74 | 0.67 | 1 | 1 |
| RNA splicing, via transesterification reactions with bulg | 1.23 | 1.33 | 1 | 1 |
| regulation of insulin receptor signaling pathway | 0.25 | 0 | 1 | 1 |
| establishment of T cell polarity | 0.25 | 0 | 1 | 1 |
| establishment of epithelial cell polarity | 0.25 | 0 | 1 | 1 |
| regulation of alpha-beta T cell proliferation | 0.25 | 0 | 1 | 1 |
| cilium biogenesis | 0.25 | 0 | 1 | 1 |
| regulation of tumor necrosis factor-beta biosynthetic pro | 0.25 | 0 | 1 | 1 |
| complement activation | 0.99 | 0.67 | 1 | 1 |
| transformed cell apoptosis | 0.49 | 0 | 1 | 1 |
| regulation of interleukin-1 biosynthetic process | 0.25 | 0 | 1 | 1 |
| mitotic metaphase/anaphase transition | 0.25 | 0 | 1 | 1 |
| mitotic metaphase | 0.25 | 0 | 1 | 1 |
| histidine catabolic process | 0.49 | 0.67 | 1 | 1 |

| | | | | |
|---|---|---|---|---|
| glycogen biosynthetic process | 0.25 | 0 | 1 | 1 |
| long-chain fatty acid biosynthetic process | 0.25 | 0 | 1 | 1 |
| midbrain-hindbrain boundary development | 0.25 | 0 | 1 | 1 |
| UTP metabolic process | 0.49 | 0.67 | 1 | 1 |
| pyrimidine ribonucleoside triphosphate biosynthetic pro | 0.49 | 0.67 | 1 | 1 |
| regulation of interleukin-4 biosynthetic process | 0.25 | 0 | 1 | 1 |
| female meiosis | 0.49 | 0 | 1 | 1 |
| positive regulation of fatty acid metabolic process | 0.25 | 0 | 1 | 1 |
| osteoblast differentiation | 0.74 | 0.67 | 1 | 1 |
| purine ribonucleoside diphosphate biosynthetic process | 0.25 | 0 | 1 | 1 |
| serotonin metabolic process | 0.25 | 0 | 1 | 1 |
| eye photoreceptor cell differentiation | 0.49 | 0.67 | 1 | 1 |
| regulation of fibroblast growth factor receptor signaling | 0.25 | 0 | 1 | 1 |
| negative regulation of Ras protein signal transduction | 0.25 | 0 | 1 | 1 |
